# Supplementary material for: Rationally designed azobenzene photoswitches for efficient two-photon neuronal excitation
Source: Nat Commun. 2019 Feb 22;10:907. doi: 10.1038/s41467-019-08796-9 (PMC6385291; doi:10.1038/s41467-019-08796-9)
Supplement: Supplementary file 1 — Supplementary Information [file 41467_2019_8796_MOESM1_ESM.pdf]

Supplementary Information for:

Rationally designed azobenzene photoswitches for efficient two-photon neuronal excitation

Cabré *et al.*

Supplementary Figures

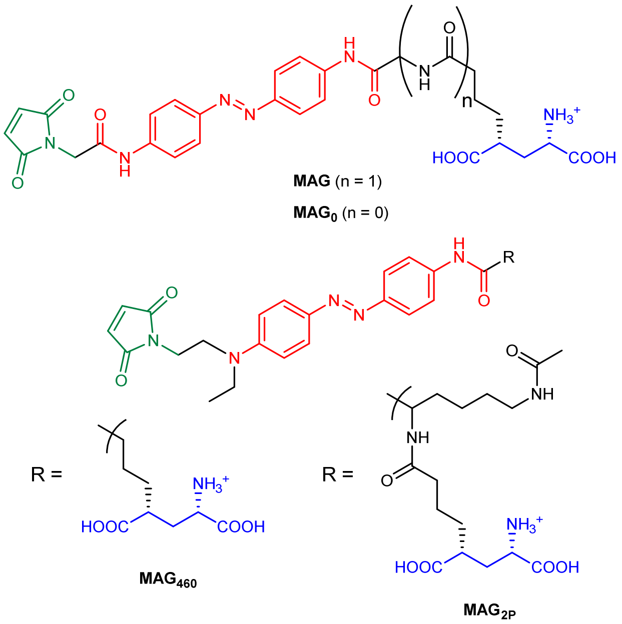

Supplementary Figure 1 Structures of MAG, MAG_0, MAG_{2P} and MAG_{460}.

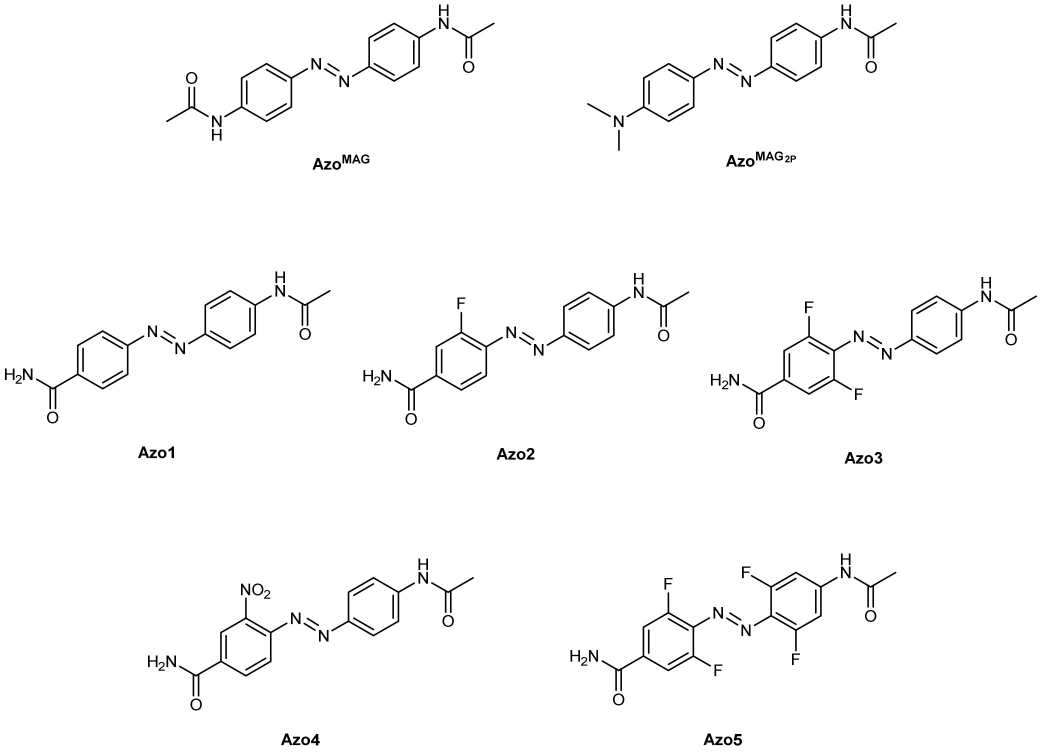

Supplementary Figure2 Structures of the model azo compounds.

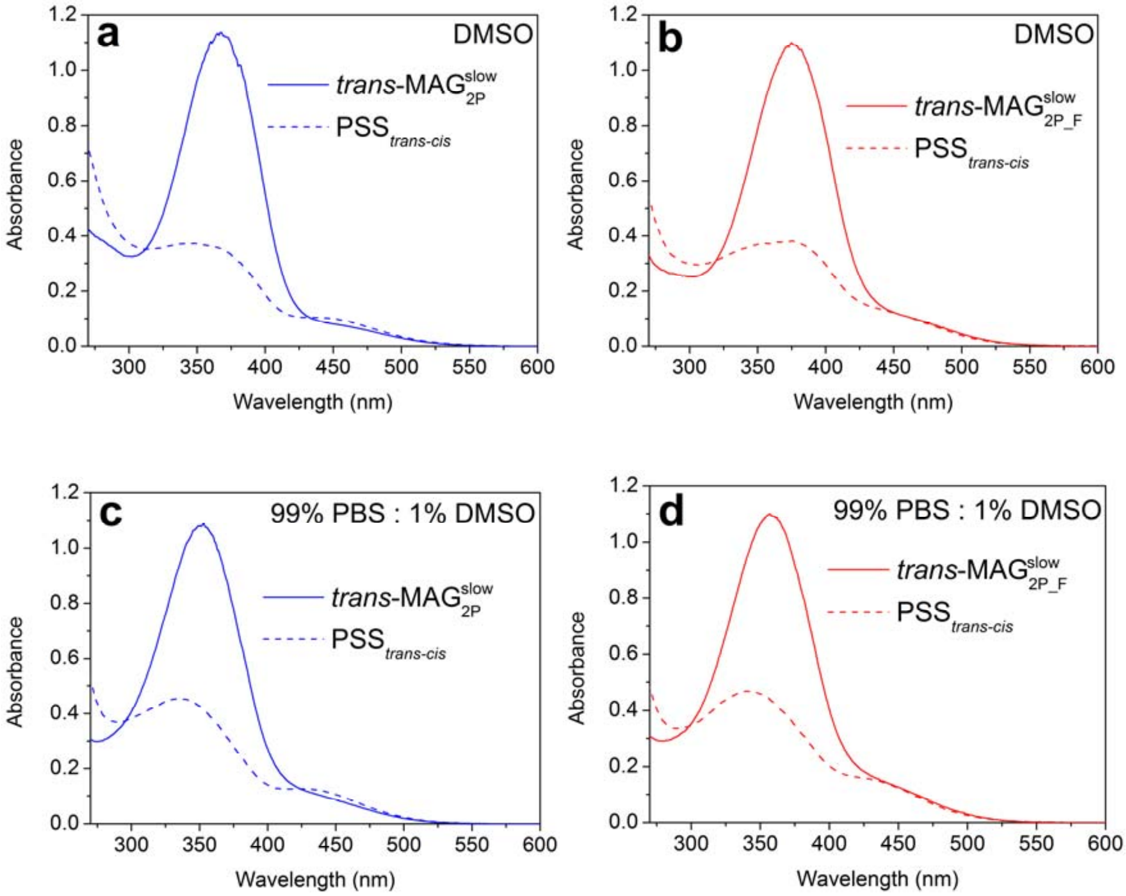

Supplementary Figure 3 *Trans→cis* photoisomerization of $\mathbf{MAG_{2P}^{slow}}$ and $\mathbf{MAG_{2P\_F}^{slow}}$. (**a, c**) Absorption spectra of *trans*-$\mathbf{MAG_{2P}^{slow}}$ and the photostationary state mixture obtained upon irradiation at λ_{exc} = 366 nm to induce *trans→cis* photoisomerization ($PSS_{\textit{trans-cis}}$) in (**a**) DMSO and (**c**) 99% PBS:1% DMSO. (**b, d**) Absorption spectra of *trans*-$\mathbf{MAG_{2P\_F}^{slow}}$ and the $PSS_{\textit{trans-cis}}$ mixture obtained upon irradiation at λ_{exc} = 366 nm in (b) DMSO and (**d**) 99% PBS:1% DMSO.

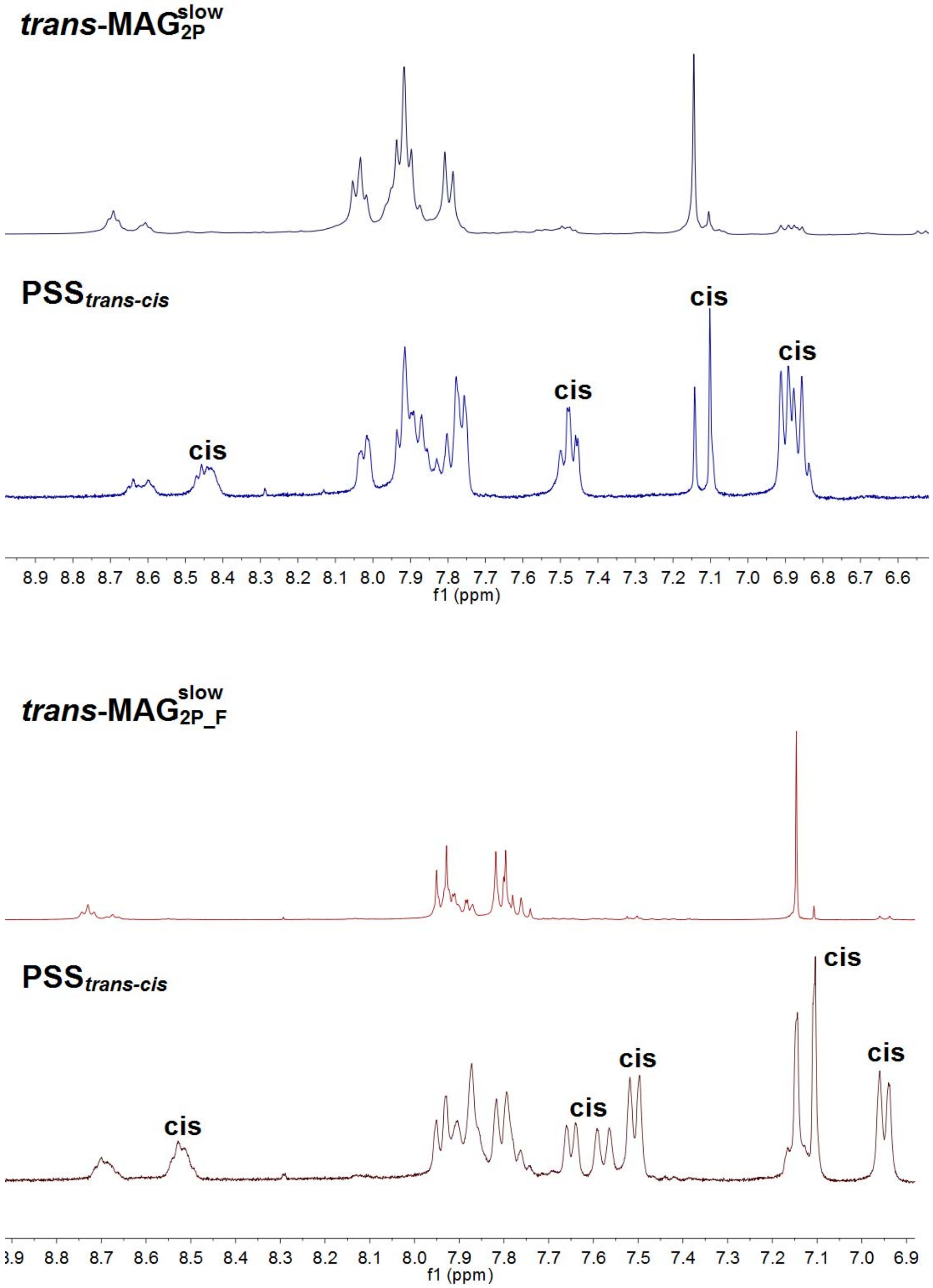

Supplementary Figure 4 *Trans→cis* photoisomerization **of MAG$^{slow}_{2P}$** and **MAG$^{slow}_{2P_F}$**. ^{1}H NMR spectra (400 MHz, DMSO-d$_6$) of *trans*-**MAG$^{slow}_{2P}$** and *trans*-**MAG$^{slow}_{2P_F}$** and the corresponding *trans-cis* photostationary state mixtures prepared by irradiation at λ_{exc} = 366 nm.

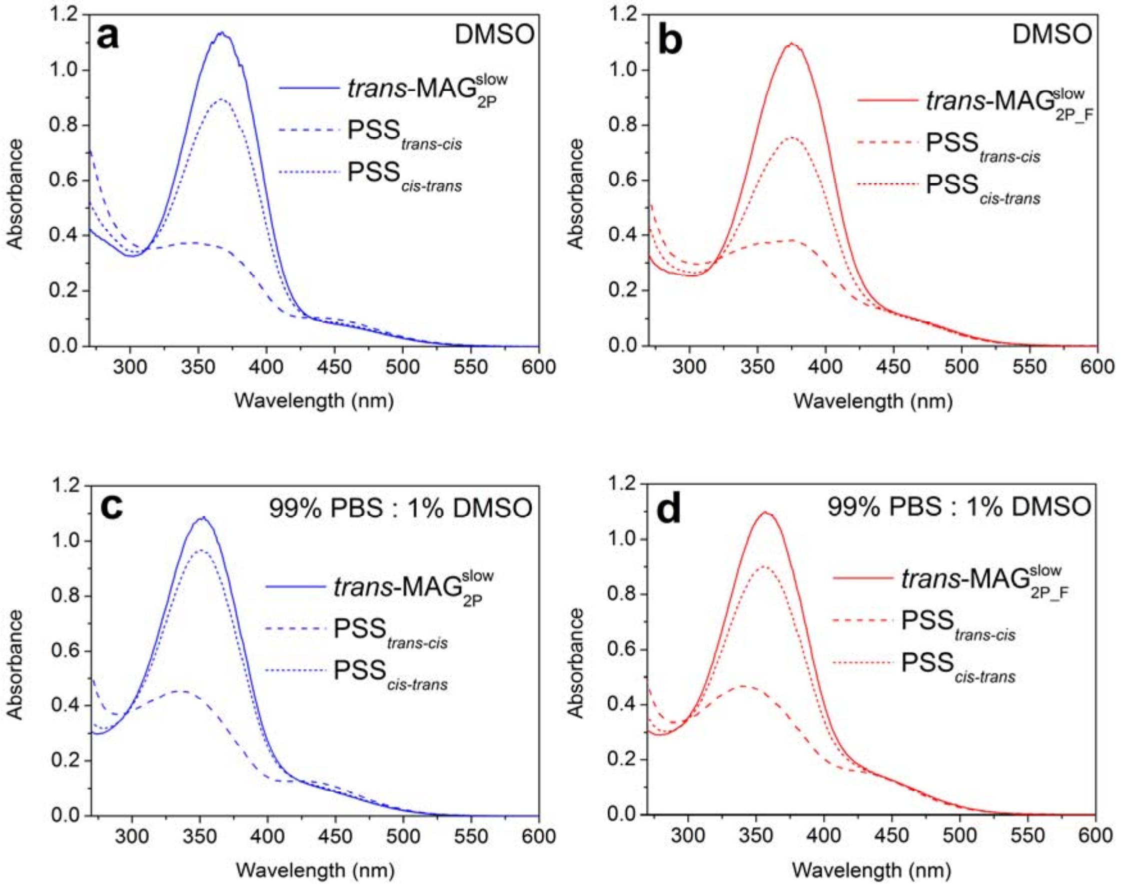

Supplementary Figure 5 *Cis→trans* photoisomerization of $\mathbf{MAG_{2P}^{slow}}$ and $\mathbf{MAG_{2P\_F}^{slow}}$. (**a, c**) Absorption spectra of *trans*-$\mathbf{MAG_{2P}^{slow}}$, the photostationary state mixture obtained upon irradiation at λ_{exc} = 366 nm to induce *trans→cis* photoisomerization ($PSS_{trans\text{-}cis}$), and the photostationary state mixture obtained upon irradiation at λ_{exc} = 473 nm to induce *cis→trans* photoisomerization ($PSS_{cis\text{-}trans}$) in (**a**) DMSO and (c) 99% PBS:1% DMSO. (**b, d**) Absorption spectra of *trans*-$\mathbf{MAG_{2P\_F}^{slow}}$, the $PSS_{trans\text{-}cis}$ mixture obtained upon irradiation at λ_{exc} = 366 nm, and the $PSS_{cis\text{-}trans}$ mixture obtained upon irradiation at λ_{exc} = 473 nm in (b) DMSO and (**d**) 99% PBS:1% DMSO.

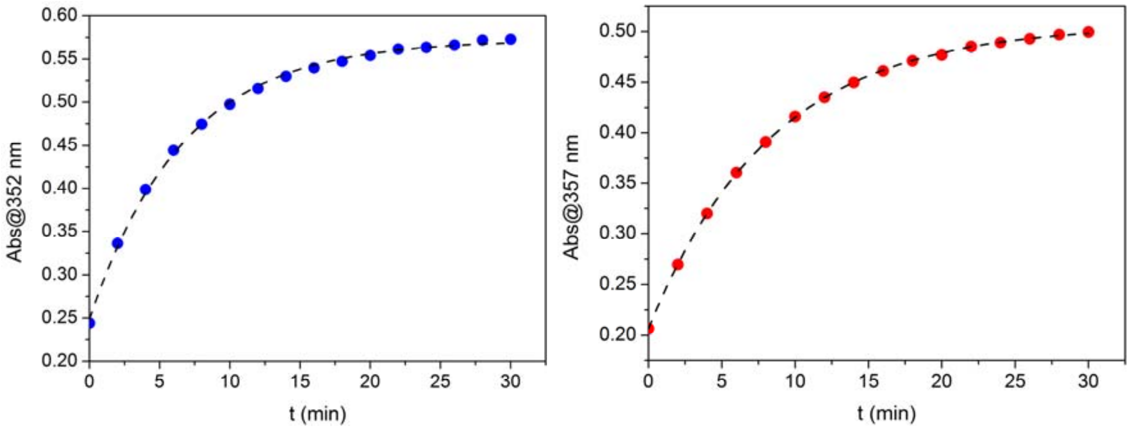

Supplementary Figure 6 *Cis-trans* thermal back-isomerization of $\mathbf{MAG_{2P}^{slow}}$ **and** $\mathbf{MAG_{2P\_F}^{slow}}$. Variation of the absorption of the *trans-cis* photostationary state mixture of $\mathbf{MAG_{2P}^{slow}}$ (blue) and $\mathbf{MAG_{2P\_F}^{slow}}$ (red) in the dark at 25 ºC in 99% PBS:1% DMSO. At these conditions, thermal *cis-trans* back-isomerization takes place, thus restoring the inital concentration of the *trans* state of the ligands, which presents a larger extinction coefficient at λ_{abs} = 352 nm and λ_{abs} = 357 nm. Points correspond to the experimental data, while lines were obtained from monoexponential fits.

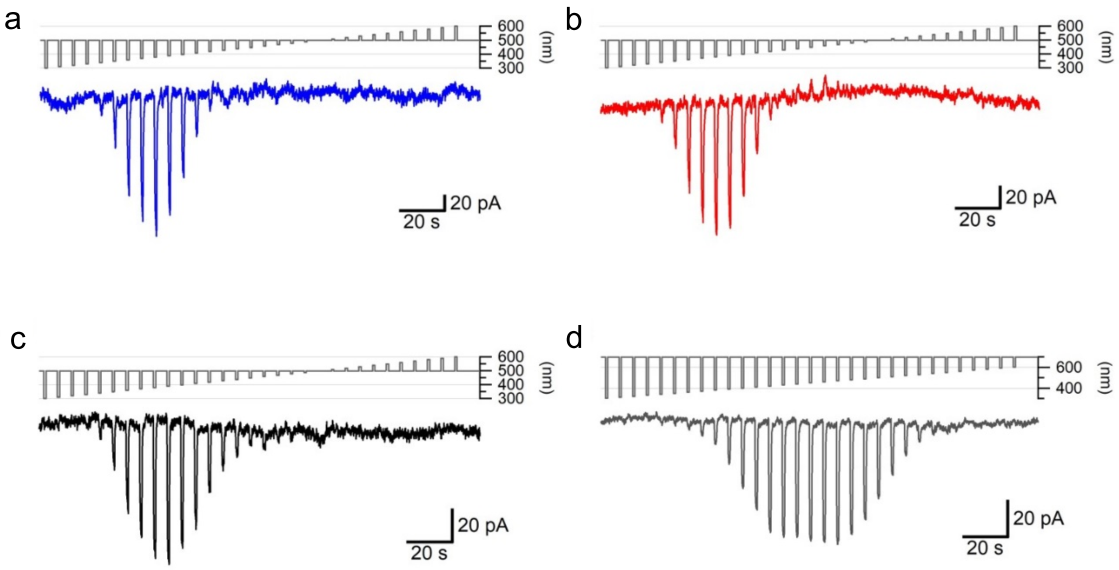

Supplementary Figure 7 1P patch-clamp traces of LiGluR-expressing HEK293 cells**.** Wavelength-dependent whole-cell voltage-clamp currents recorded in HEK293 cells expressing GluK2-L439C after conjugation to (**a**) $\mathbf{MAG_{2P}^{slow}}$, (**b**) $\mathbf{MAG_{2P\_F}^{slow}}$, (**c**) **MAG**, and (**d**) $\mathbf{MAG_{2P}}$. Excitation light pulses to induce channel opening ranged from 300 to 600 nm, while channel closure was accomplished by excitation at 500 nm in (**a-c**) and thermally in (**d**).

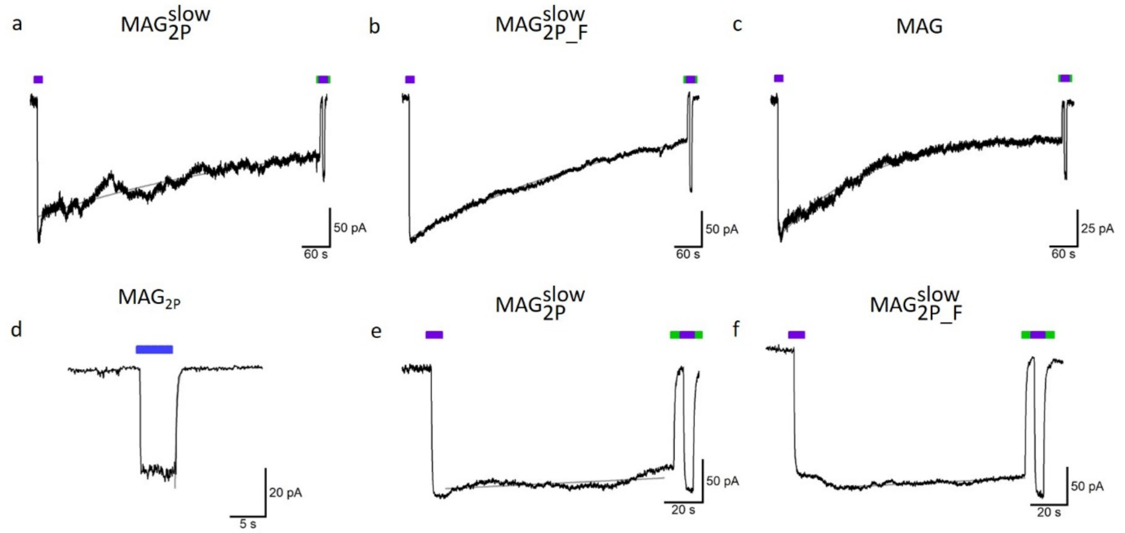

Supplementary Figure 8 1P thermal relaxation of in LiGluR-expressing HEK293 cells**.** Electrophysiological recordings and quantification of thermal relaxation lifetimes at room temperature of $\mathbf{MAG_{2P}^{slow}}$, $\mathbf{MAG_{2P\_F}^{slow}}$, **MAG,** and $\mathbf{MAG_{2P}}$ photoswitches after conjugation to GluK2-L439C. Time course of photocurrent relaxation in the dark after initial 380 nm light pulse (whole cell patch clamp in HEK293 cells). Exponential fits are indicated by grey curves. The slow thermal relaxation of *cis* isomers of (**a**) $\mathbf{MAG_{2P}^{slow}}$, (**b**) $\mathbf{MAG_{2P\_F}^{slow}}$, and (**c**) **MAG**, confirms slow deactivation in the dark (τ = 570 ± 14 s, 534 ± 3 s, and 900 ± 54 s respectively), in agreement with measurements in cuvette (Supplementary Figure 6). A second light pulse at the end of the traces shows that partial current run-down occurs in these long-lasting current recordings. This is usually due to cell dialysis in whole cell patch clamp configuration, but in our case it does not cause overestimation of lifetimes. In contrast to these long lifetimes, $\mathbf{MAG_{2P}}$ photoswitch (**d**) relaxes much faster in the dark and yields an average lifetime of 0.25 ± 0.013 s. Current rundown and stability of $\mathbf{MAG_{2P}^{slow}}$ and $\mathbf{MAG_{2P\_F}^{slow}}$ can be improved in shorter relaxation experiments (2 min in the dark, (**e**) and (**f**) respectively) but do not allow determining reliable lifetime values. However, they show that in contrast to fast relaxing photoswitches, $\mathbf{MAG_{2P}^{slow}}$ and $\mathbf{MAG_{2P\_F}^{slow}}$ induce stable channel activation with short light pulses, as intended to achieve higher 2P excitation efficacy.

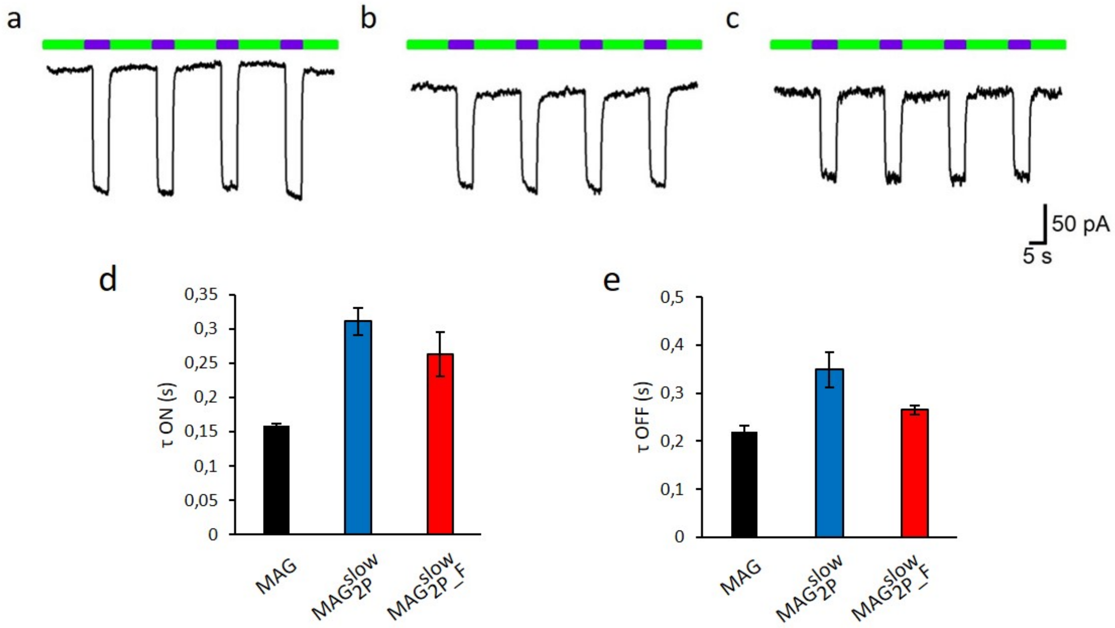

Supplementary Figure 9 Activation and deactivation kinetics in LiGluR-expressing HEK293 cells. Whole cell patch-clamp current traces from HEK293 cells expressing GluK2-L439C after conjugation to (**a**) **MAG**, (**b**) $\mathbf{MAG_{2P}^{slow}}$ and, (**c**) $\mathbf{MAG_{2P\_F}^{slow}}$. Cells were treated with 0.3 mg/mL Concanavalin A to block receptor desensitization and obtain steady currents. The photocurrent onset and offset were fitted to an exponential function to calculate τ_{ON} and τ_{OFF}, respectively. Light stimulation wavelengths were 405 nm (purple bars, 38 μW mm^{-2}) and 500 nm (green bars, 48 μW mm^{-2}). (**d, e**) Quantification of τ_{ON} and τ_{OFF} for **MAG** (black bars, *n* = 4 biologically independent cells), $\mathbf{MAG_{2P}^{slow}}$ (blue bars, *n* = 5 biologically independent cells), and $\mathbf{MAG_{2P\_F}^{slow}}$ (red bars, *n* = 3 biologically independent cells). Error bars are SEM. Source data for (**d, e**) are provided as a soured Data file.

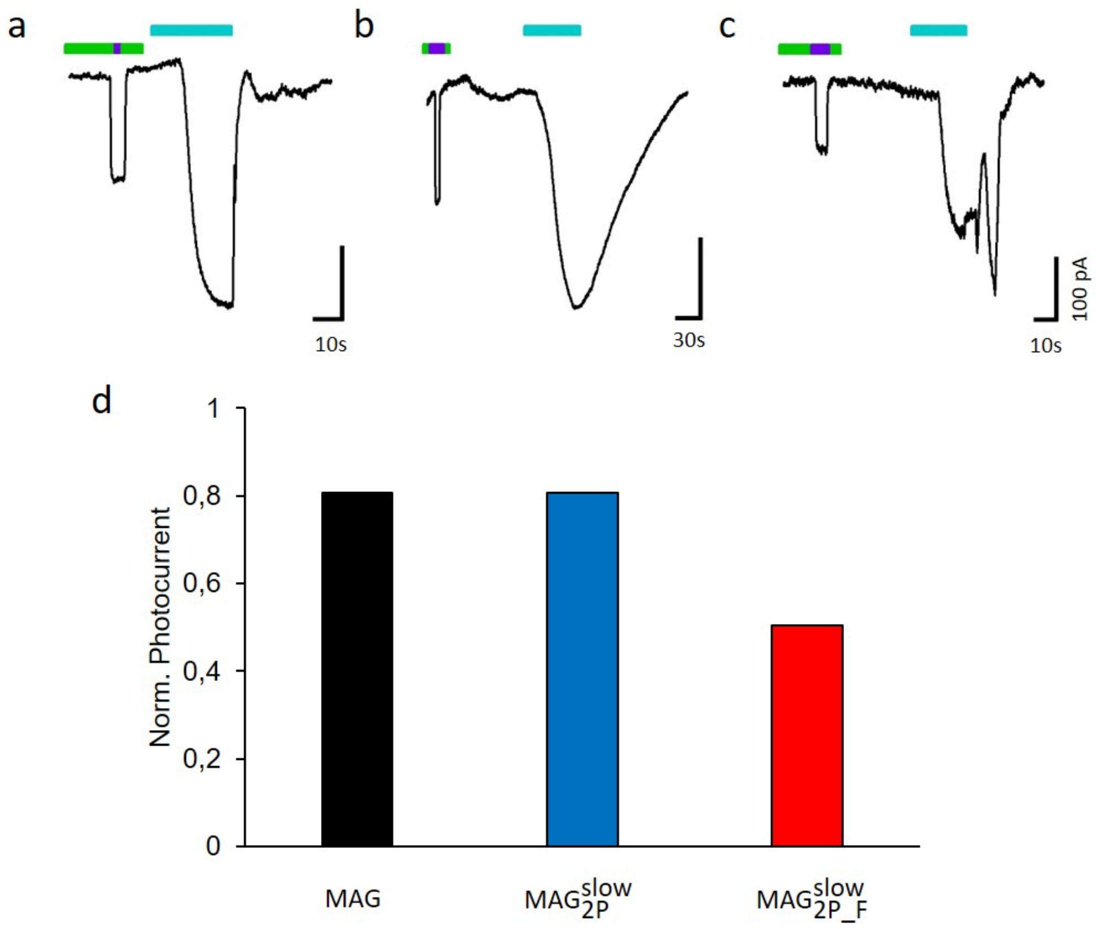

Supplementary Figure 10 1P patch clamp traces of LiGluR-expressing HEK293 cells**.** Whole cell patch-clamp current traces from HEK293 cells expressing GluK2-L439C after conjugation to (**a**) **MAG**, (**b**) **MAG$^{slow}_{2P}$**, (**c**) **MAG$^{slow}_{2P_F}$** (incubation with 50 µM solutions of each photoswitch). Cells were treated with 0.3 mg/mL Concanavalin A to block receptor desensitization and obtain steady currents. Light stimulation wavelengths were 405 nm (purple bars, 37.7 µW mm^{-2}) and 500 nm (green bars, 47.4 µW mm^{-2}). After photoinduced opening-closing, LiGluR activation was achieved by addition of 300 µM free glutamate. (**d**) Quantification of photocurrents normalized by the response to free glutamate (300 µM). Thus, photoswitch conjugation allows the receptor to respond to illumination, while preserving its physiological responses to free glutamate, as reported for other **MAG** analogs.[1,2]

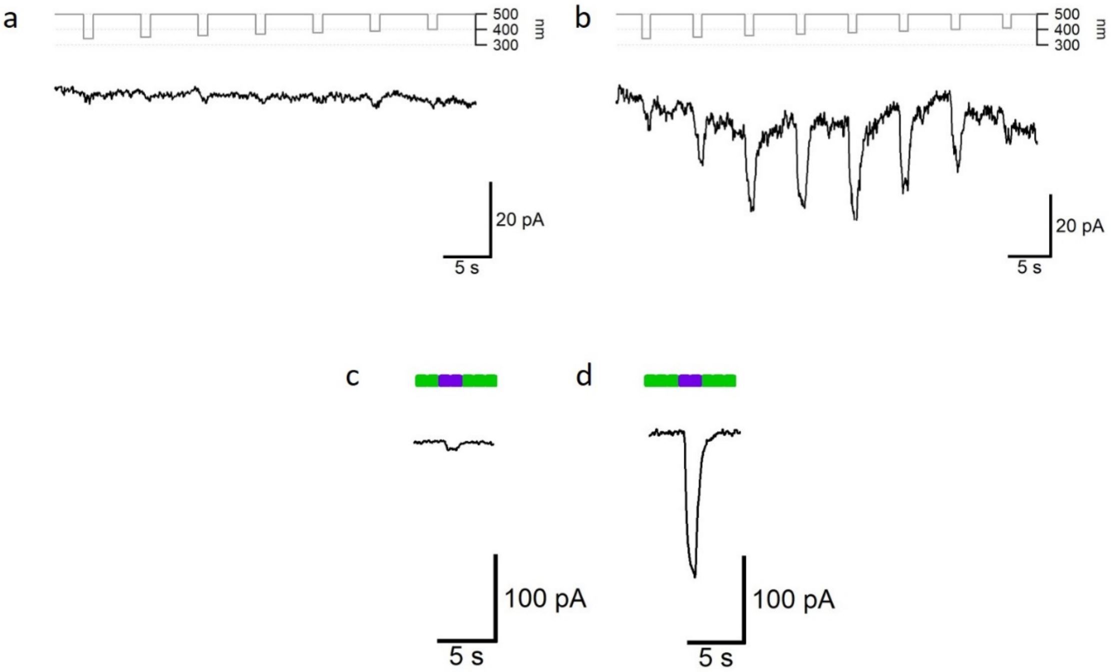

Supplementary Figure 11 Desensitization of LiGluR after conjugation to $\mathbf{MAG_{2P}^{slow}}$. Example of wavelength-dependent photoresponses of $\mathbf{MAG_{2P}^{slow}}$ conjugated to GluK2-L439C in HEK293 cells and measured using whole cell patch-clamp. Action spectra with averaged photoresponses are shown in Figure 3. Prior to incubation in Concanavalin A, rapid receptor desensitization precludes detection of the fast transient responses (**a, c**), which are readily measurable as steady photocurrents upon blocking desensitization with Concanavalin A (**b**, **d**). In (**c**) and (**d**) light stimulation wavelengths were 405 nm (purple bars, 38 μW mm^{-2}) and 500 nm (green bars, 48 μW mm^{-2}). Conjugation of $\mathbf{MAG_{2P}^{slow}}$ to GluK2-L439C does not slow down desensitization, in agreement to a previous report using MAG.[3]

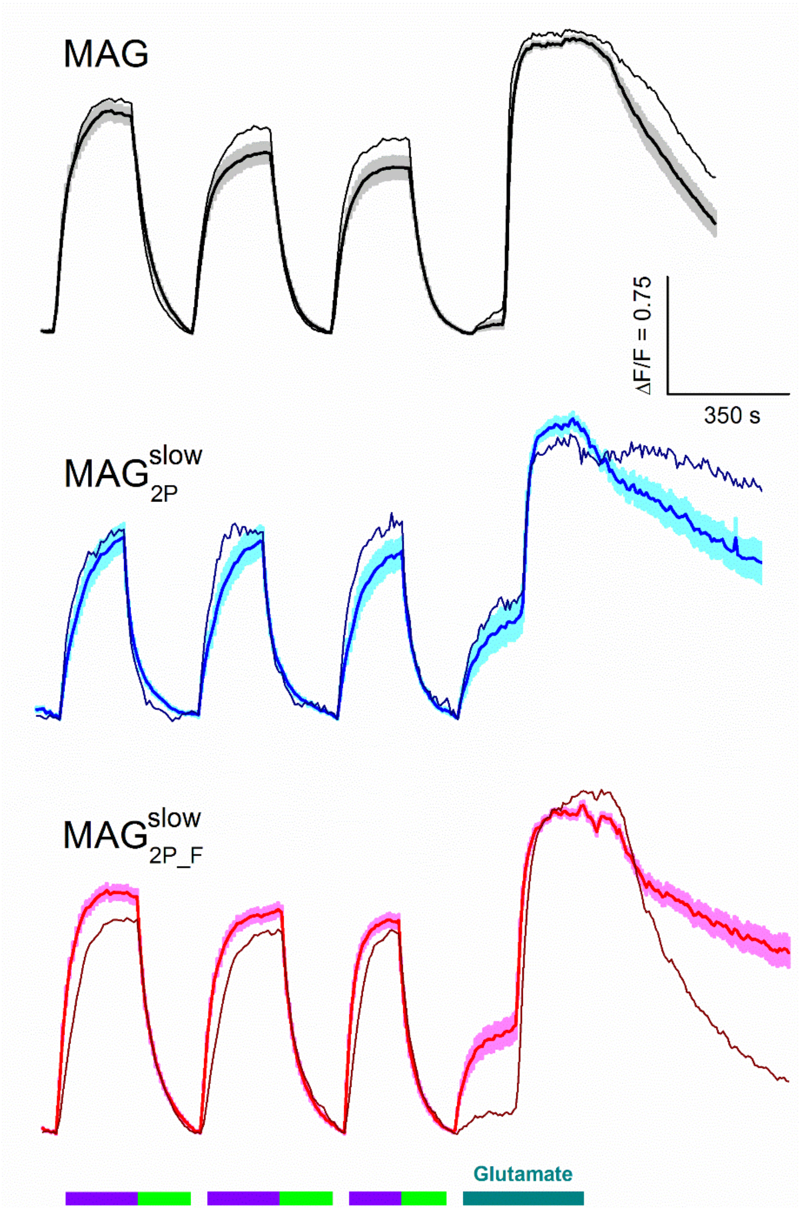

Supplementary Figure 12 1P calcium imaging traces of LiGluR-expressing HEK293 cells. Individual (thin lines) and average (thick lines) calcium imaging fluorescence traces registered for HEK293 cells co-expressing GluK2-L439C and GCaMP6s after conjugation to **MAG** (*n* = 31 biologically independent cells), **MAG$^{slow}_{2P}$** (*n* = 20 biologically independent cells) and **MAG$^{slow}_{2P_F}$** (*n* = 45 biologically independent cells) (50 μM each photoswitch). The bands around average traces plot the corresponding SEM. 1P excitation at 360 nm (violet) was applied to open LiGluR channels and trigger calcium-induced GCaMP6s fluorescence enhancement, while 1P excitation at 500 nm (green) was applied to revert back the process. After three photoinduced opening-closing cycles, LiGluR activation was achieved by addition of 300 μM free glutamate. Source data are provided as a soured Data file.

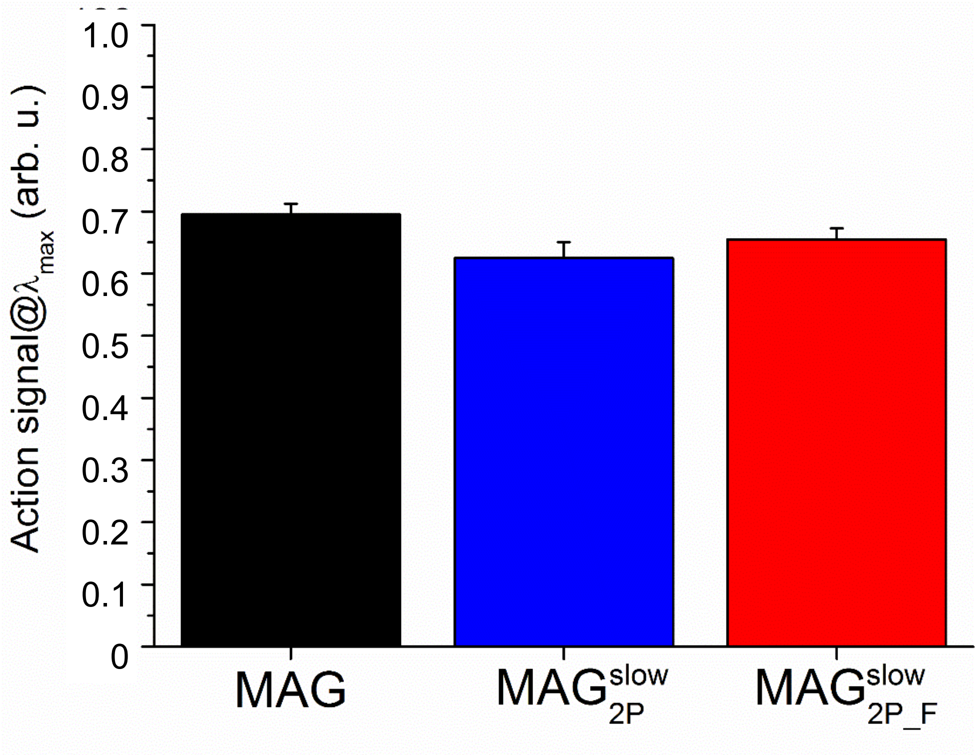

Supplementary Figure 13 1P calcium imaging responses.1P responses of **MAG**, $\mathbf{MAG_{2P}^{slow}}$ and $\mathbf{MAG_{2P\_F}^{slow}}$ after conjugation to GluK2-L439C-expressing HEK293 cells (50 µM each photoswitch). Photoresponses were measured by means of calcium imaging measurements using GCaMP6s as fluorescence indicator. Before averaging over different cells, the 1P responses of each cell were normalized with respect to the free glutamate response (300µM) (*n* = 81, 63 and 76 biologically independent cells, respectively). Errors are SEM. Source data are provided as a soured Data file.

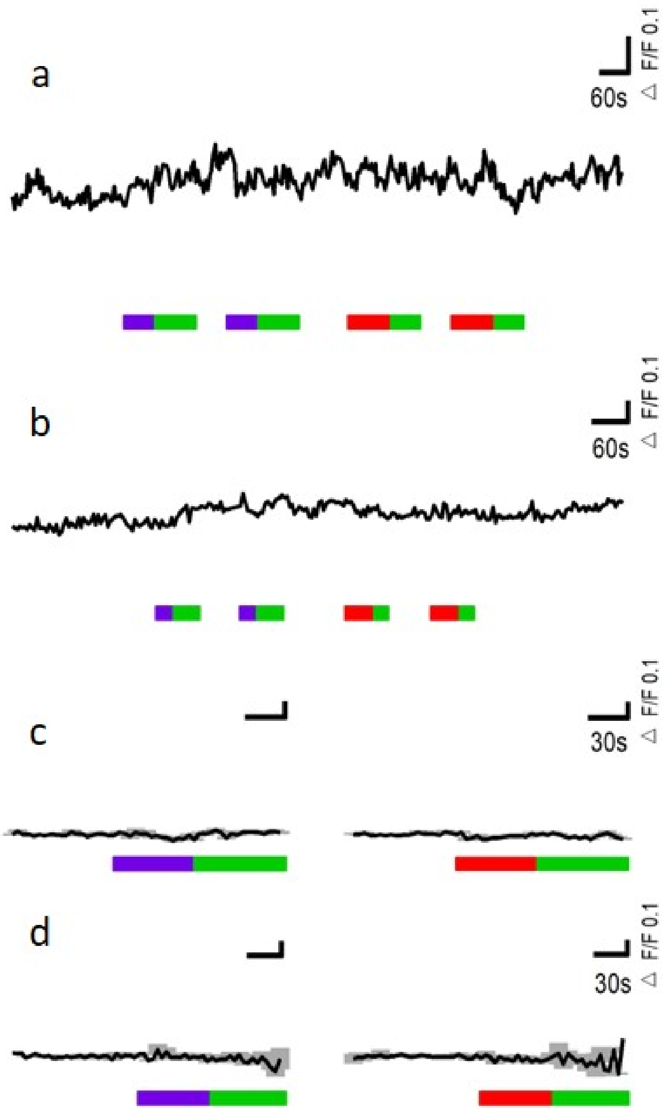

Supplementary Figure 14 Calcium imaging traces for control samples of hippocampal slices. Cells in rat hippocampal organotypic slices expressing RCaMP2 but not LiGluR-eGFP did not respond to **MAG** or $\mathbf{MAG_{2P\_F}^{slow}}$ photostimulation. (**a-b**) Real time traces of a single-cell neuronal activity of slices incubated with (**a**) **MAG** or (b) $\mathbf{MAG_{2P\_F}^{slow}}$. (**c-d**) Average 1P and 2P responses of neurons incubated with (**c**) **MAG** (*n* = 3 cells from different animals experiments) or (d) $\mathbf{MAG_{2P\_F}^{slow}}$ (*n* = 4 cells from different animals experiments). Error bars are SEM. 1P stimulation was performed at 405 nm (purple bar) and 514 nm (green bar), and 2P stimulation at 780 nm (red bar). Source data fare provided as a soured Data file.

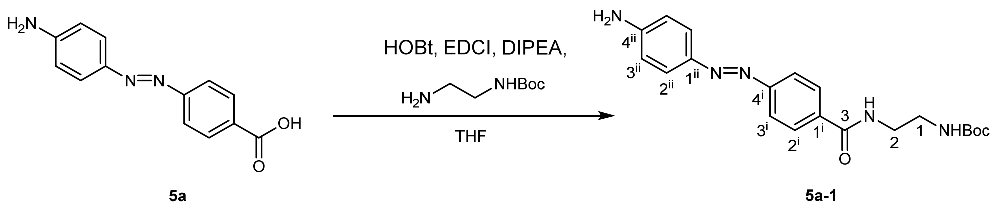

Supplementary Figure 15 Synthesis of compound **5a-1.**

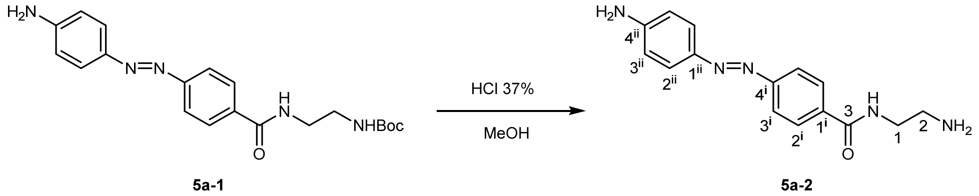

Supplementary Figure 16 Synthesis of compound **5a-2.**

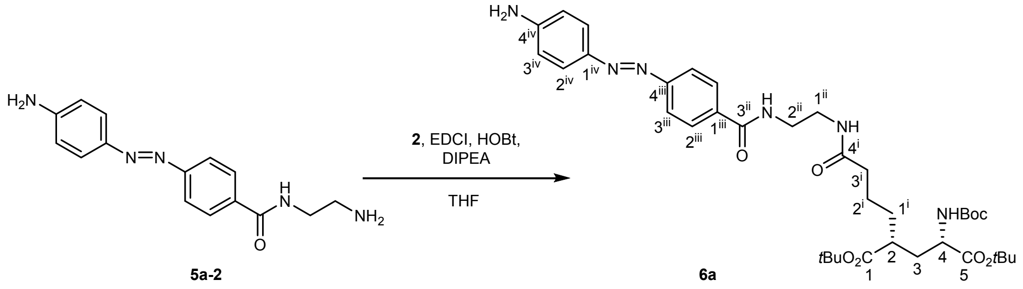

Supplementary Figure 17 Synthesis of compound **6a.**

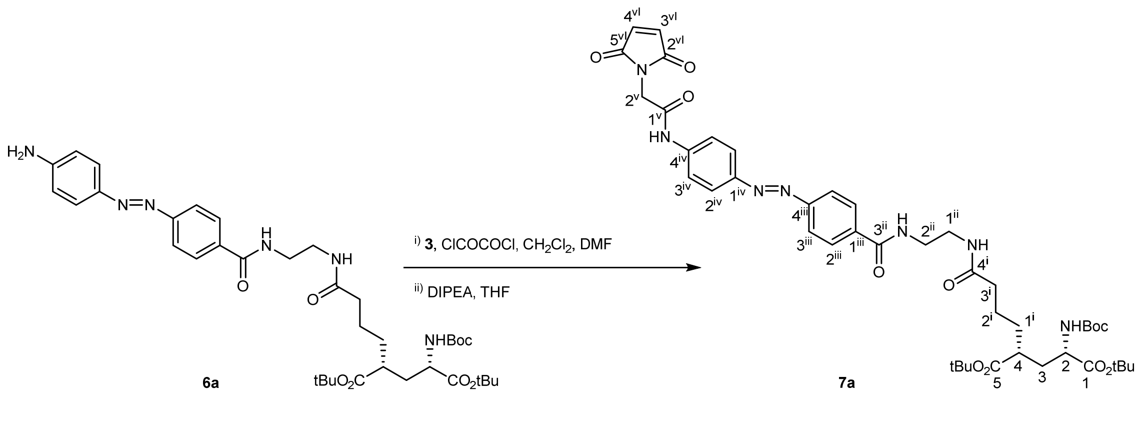

Supplementary Figure 18 Synthesis of compound **7a.**

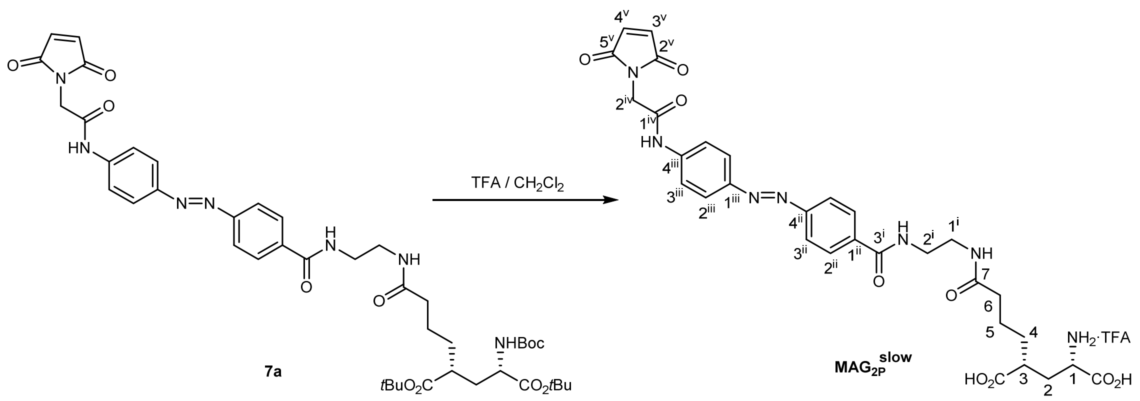

Supplementary Figure 19 Synthesis of compound **MAG$_{\mathbf{2P}}^{\mathbf{slow}}$.**

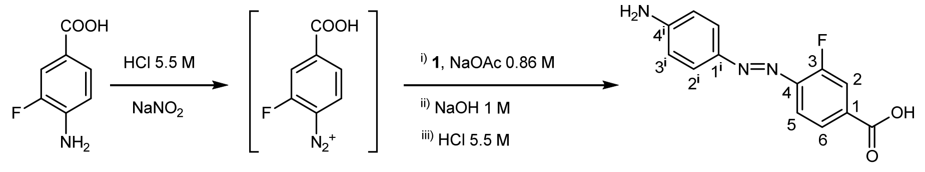

4b **4b-1** **5b**

Supplementary Figure 20 Synthesis of compound **5b.**

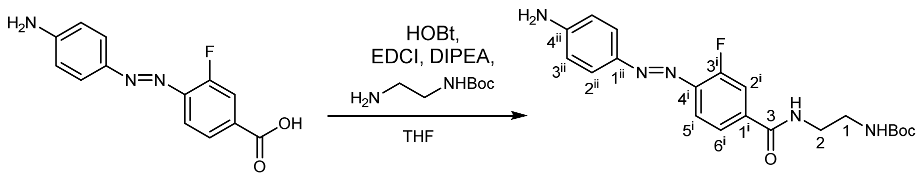

5b **5b-1**

Supplementary Figure 21 Synthesis of compound **5b-1.**

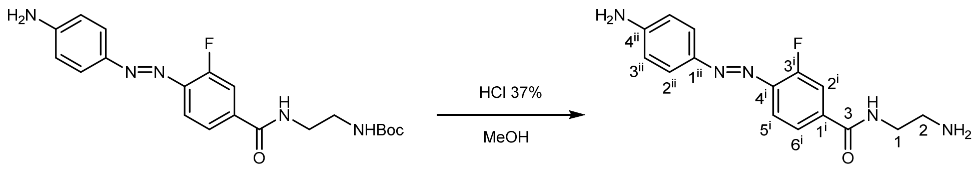

5b-1 **5b-2**

Supplementary Figure 22 Synthesis of compound **5b-2.**

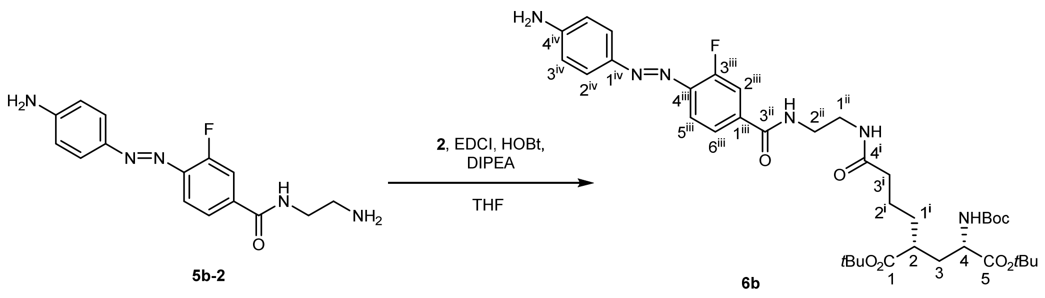

5b-2 **6b**

Supplementary Figure 23 Synthesis of compound **6b.**

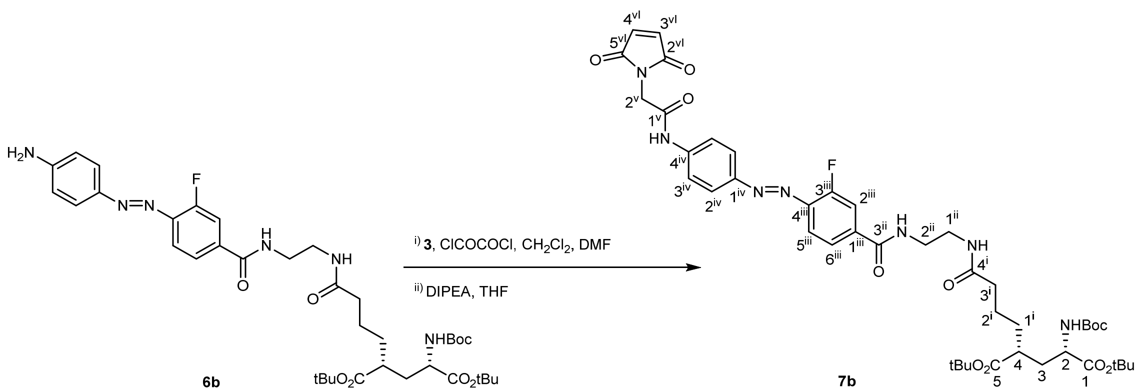

6b **7b**

Supplementary Figure 24 Synthesis of compound **7b.**

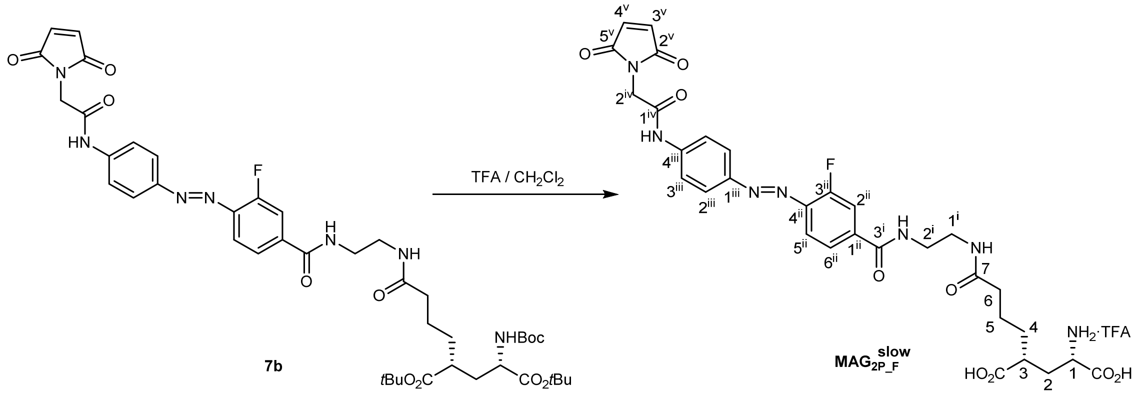

Supplementary Figure 25 Synthesis of compound **MAG$_{2P_F}^{slow}$.**

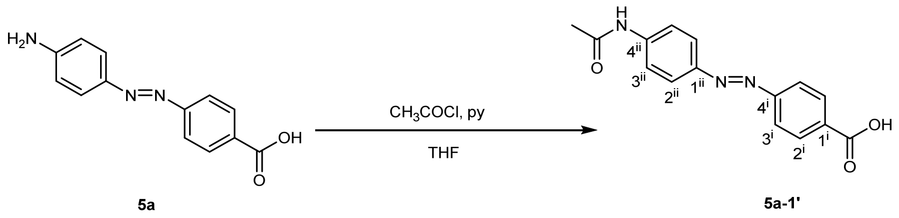

Supplementary Figure 26 Synthesis of compound **5a-1’.**

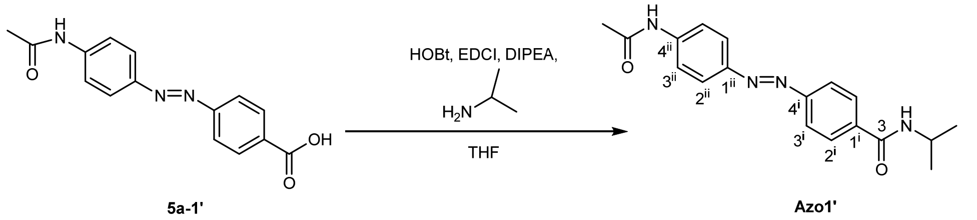

Supplementary Figure 27 Synthesis of compound **Azo1’.**

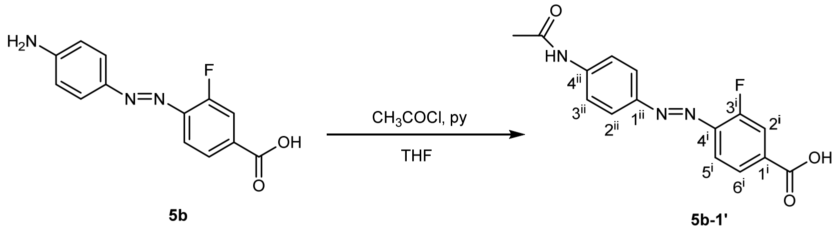

Supplementary Figure 28 Synthesis of compound **5b-1’.**

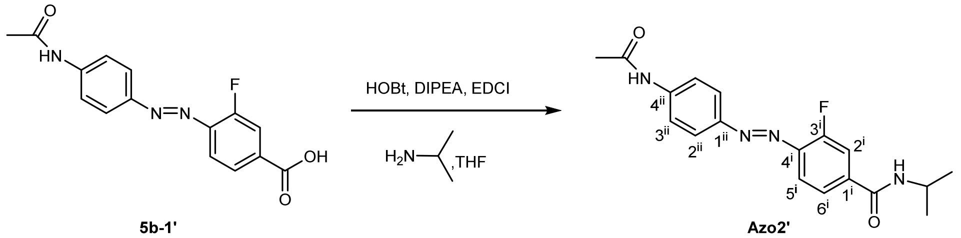

Supplementary Figure 29 Synthesis of compound **azo2’’.**

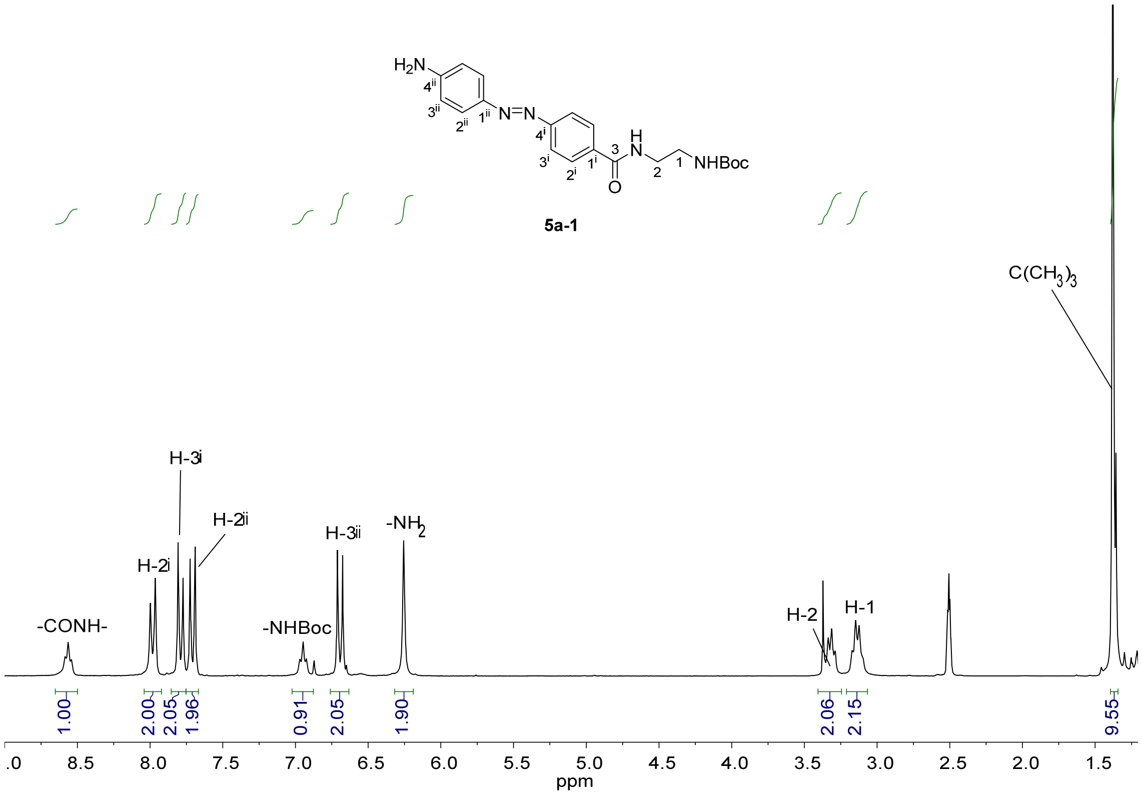

^{1}H NMR (250 MHz, DMSO-d_6)

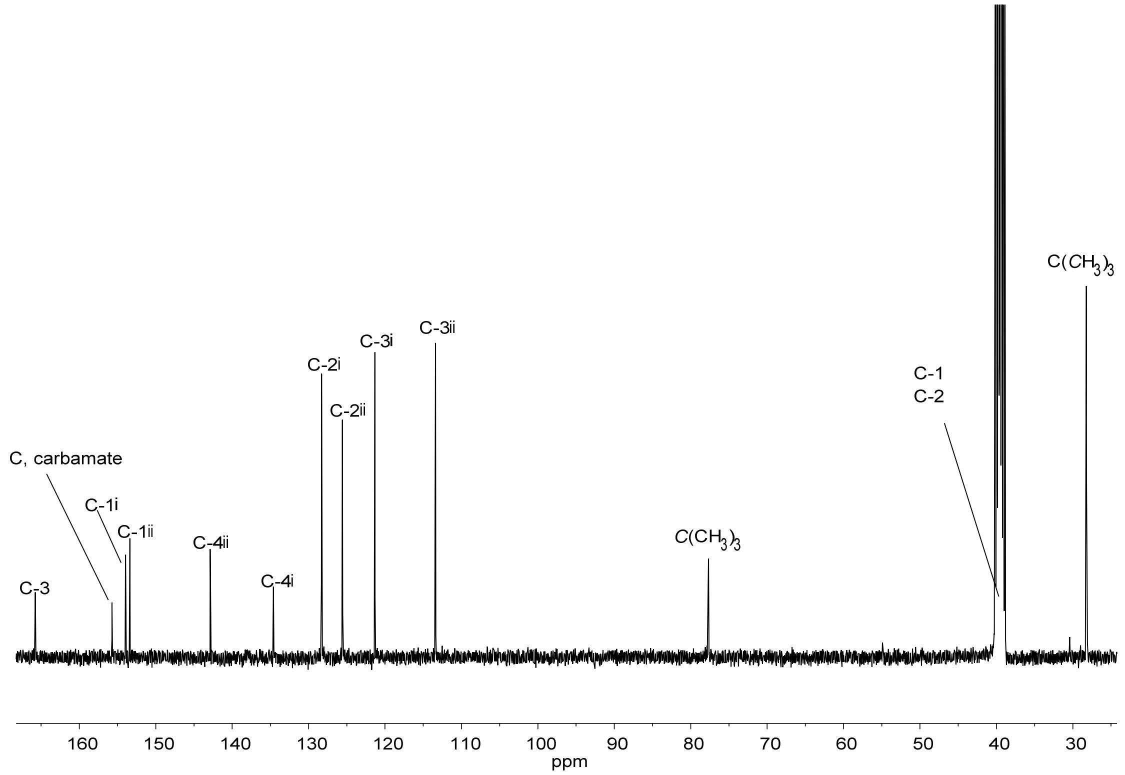

^{13}C NMR (100.6 MHz, DMSO-d_6)

Supplementary Figure 30 ^{1}H and ^{13}C NMR spectra of compound **5a-1.**

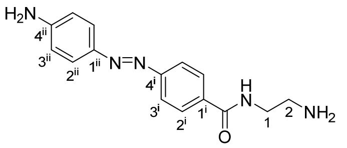

5a-2

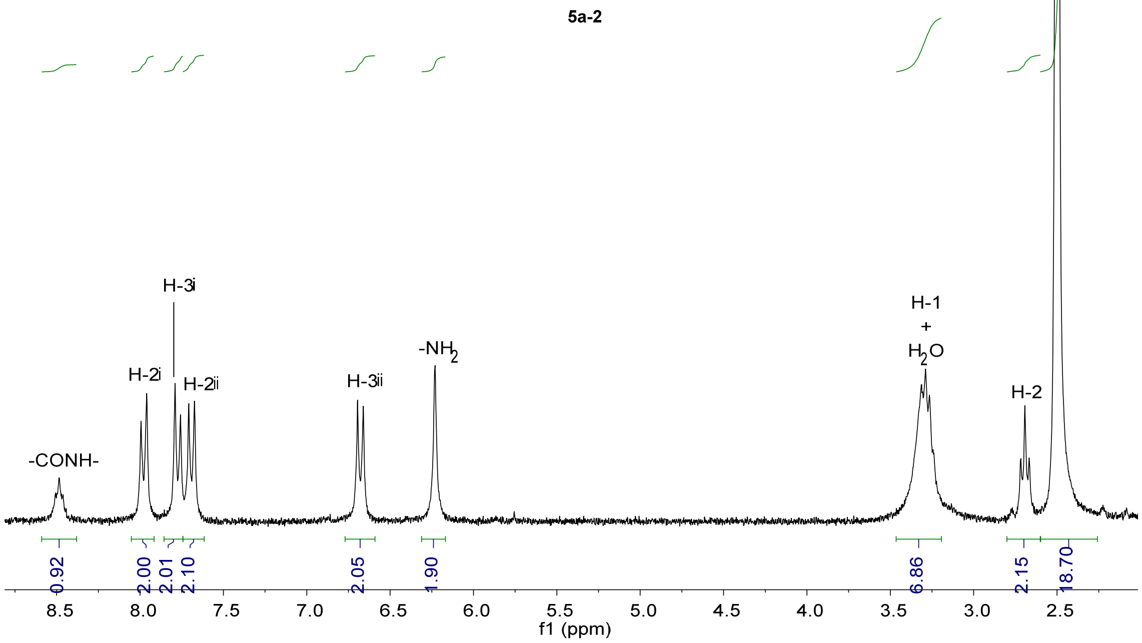

^{1}H NMR (250 MHz, DMSO-d$_6$)

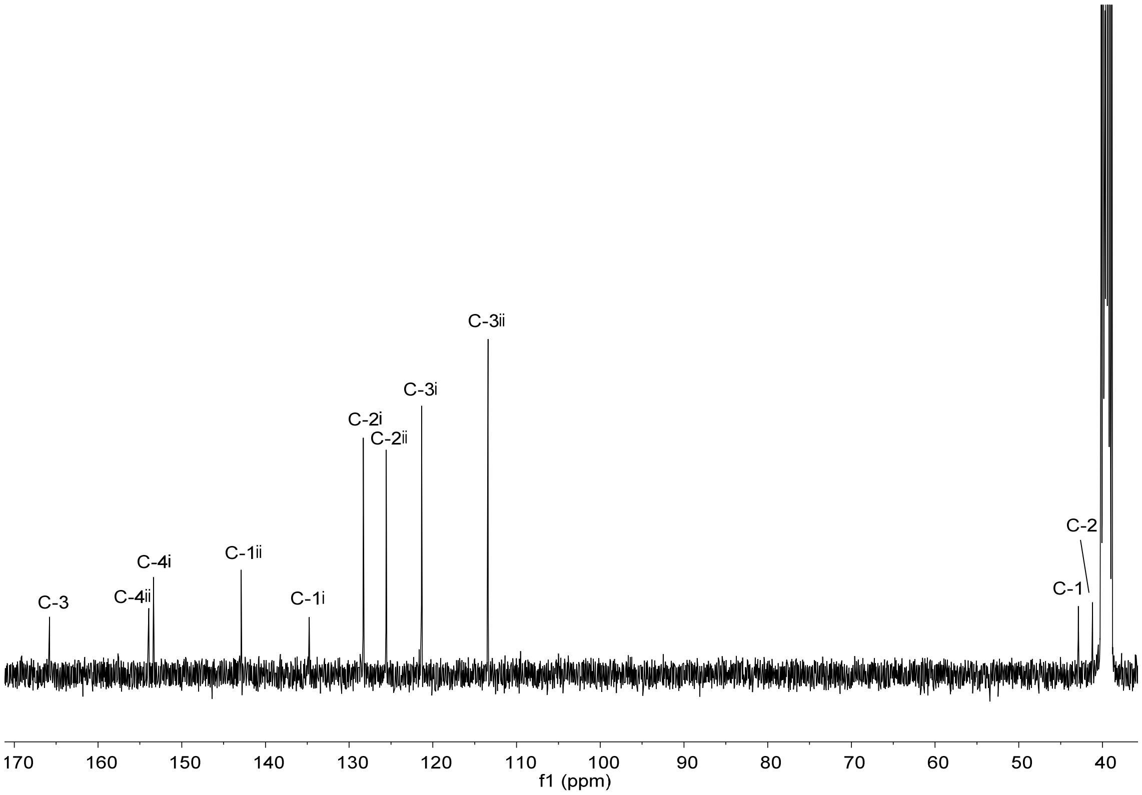

^{13}C NMR (100.6 MHz, DMSO-d$_6$)

Supplementary Figure 31 ^{1}H and ^{13}C NMR spectra of compound **5a-2.**

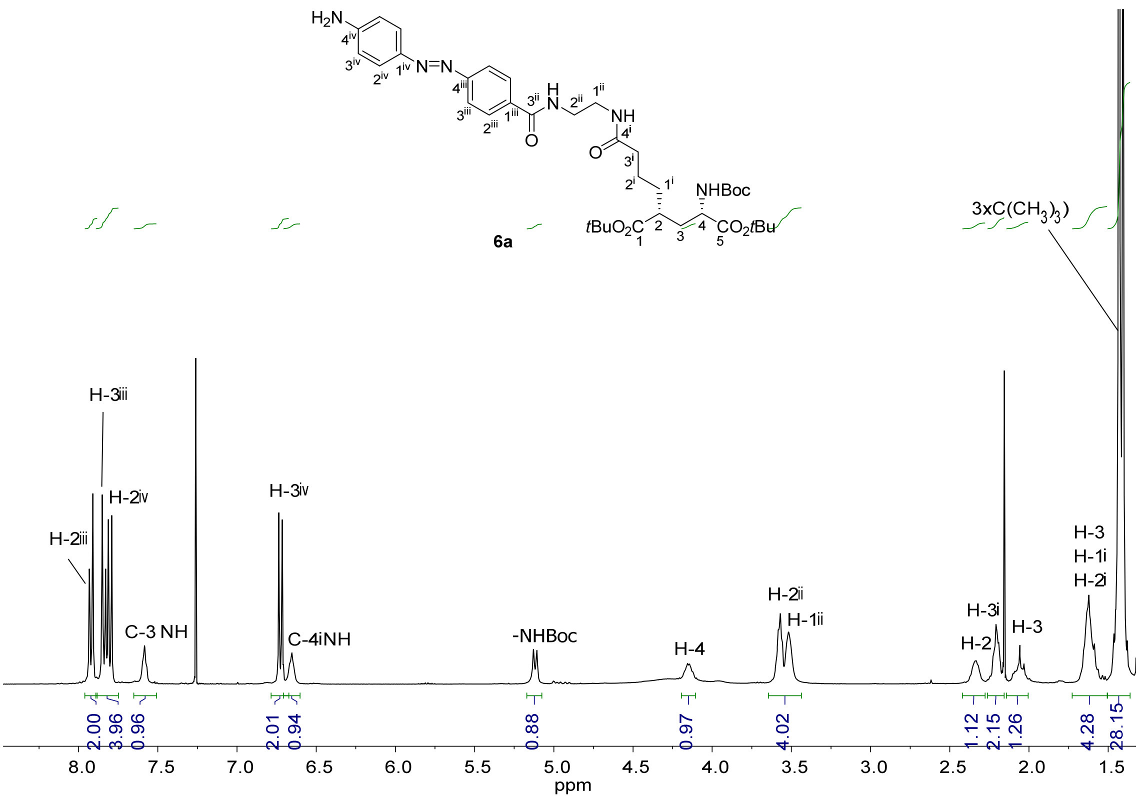

^{1}H NMR (400 MHz, $CDCl_3$)

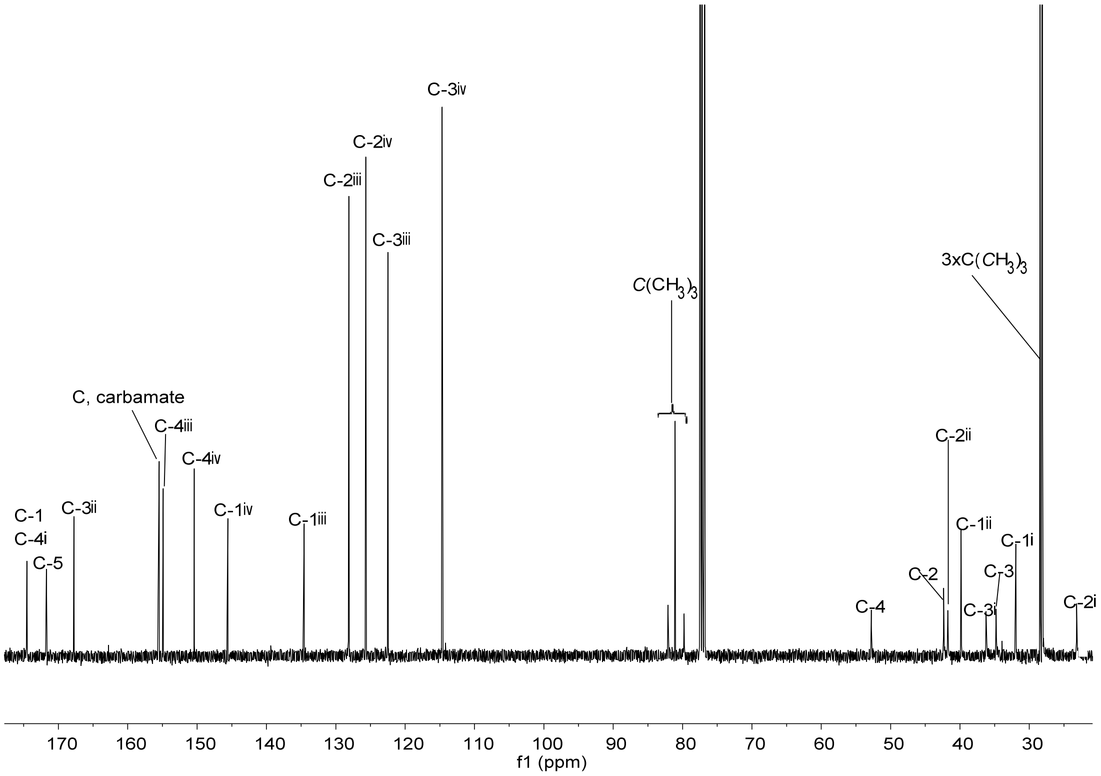

^{13}C NMR (100.6 MHz,$CDCl_3$)

Supplementary Figure 32 ^{1}H and ^{13}C NMR spectra of compound **6a.**

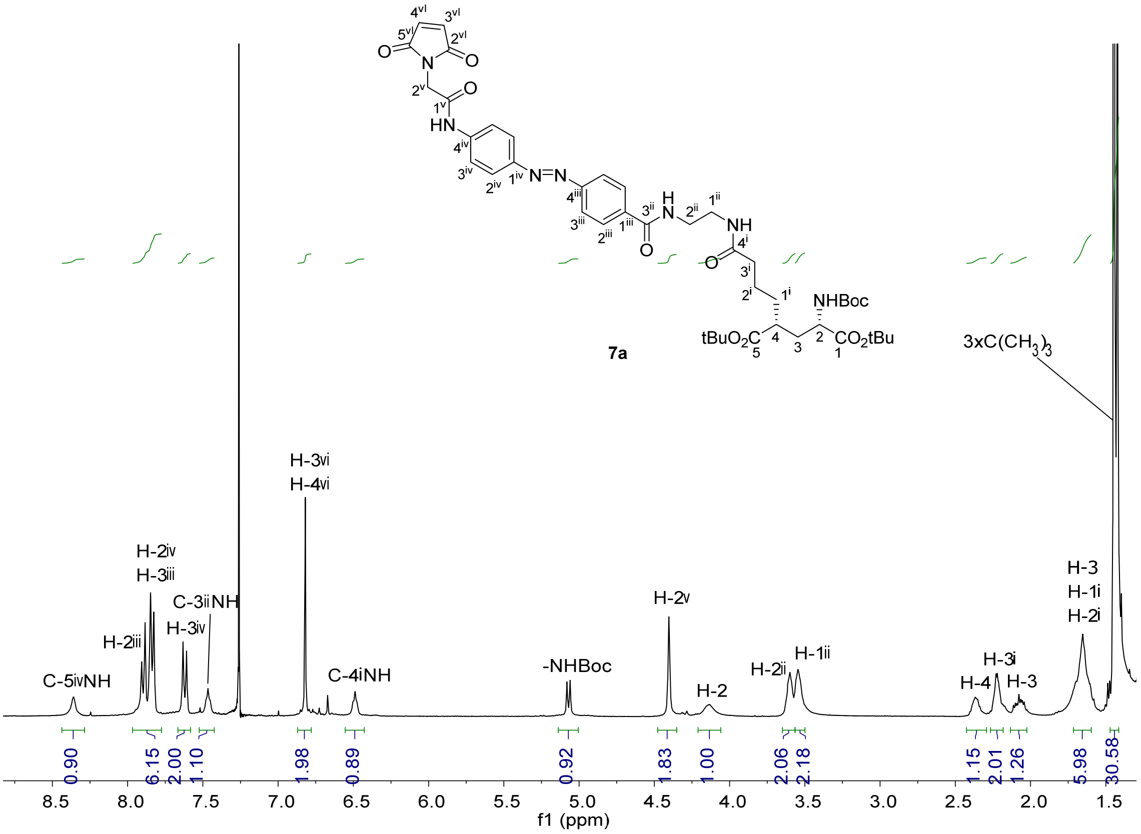

^{1}H NMR (400 MHz, $CDCl_3$, 320 K)

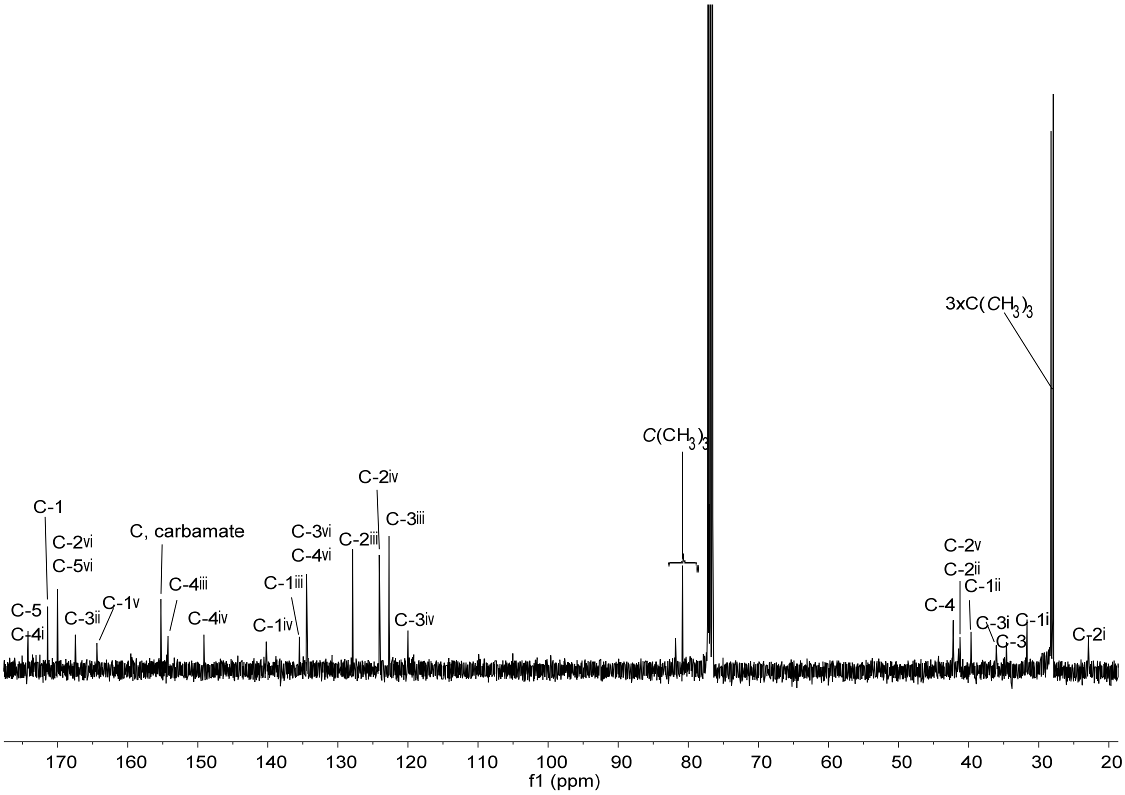

^{13}C NMR (100.6 MHz, $CDCl_3$, 320 K)

Supplementary Figure 33 ^{1}H and ^{13}C NMR spectra of compound **7a.**

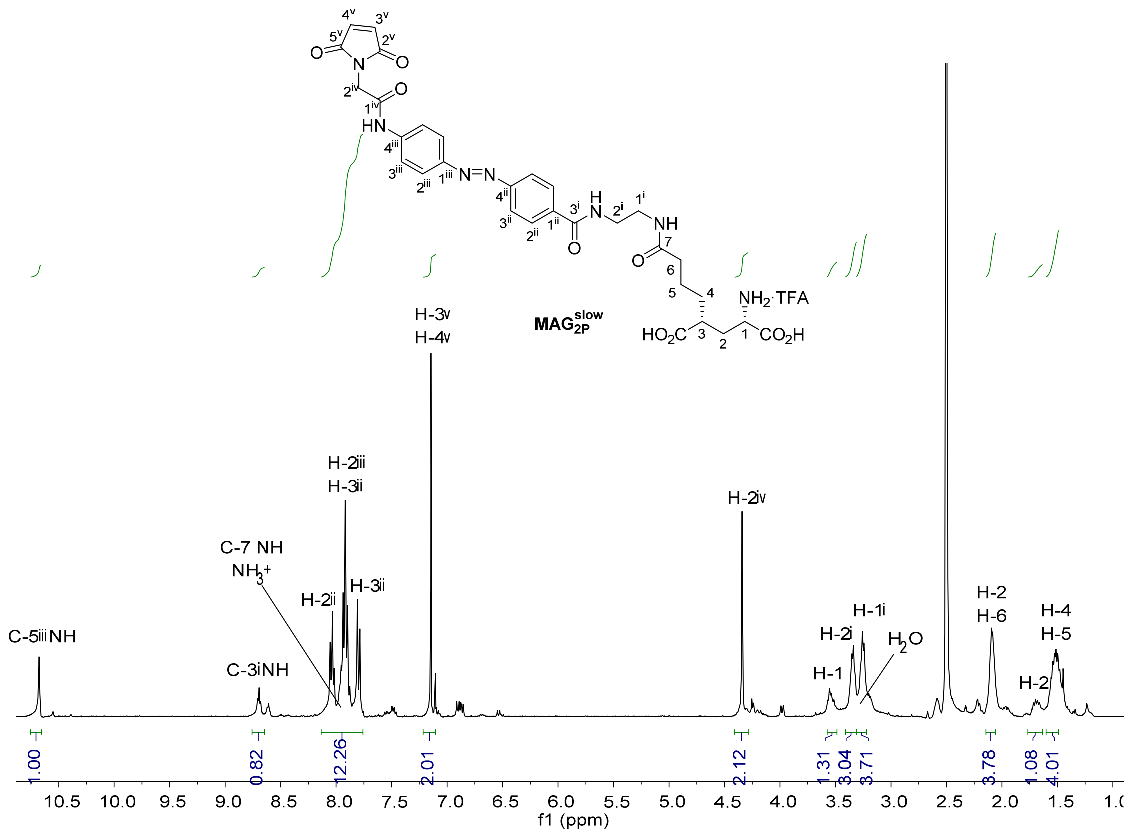

^{1}**H NMR** (400 MHz, DMSO-d$_6$, 310 K)

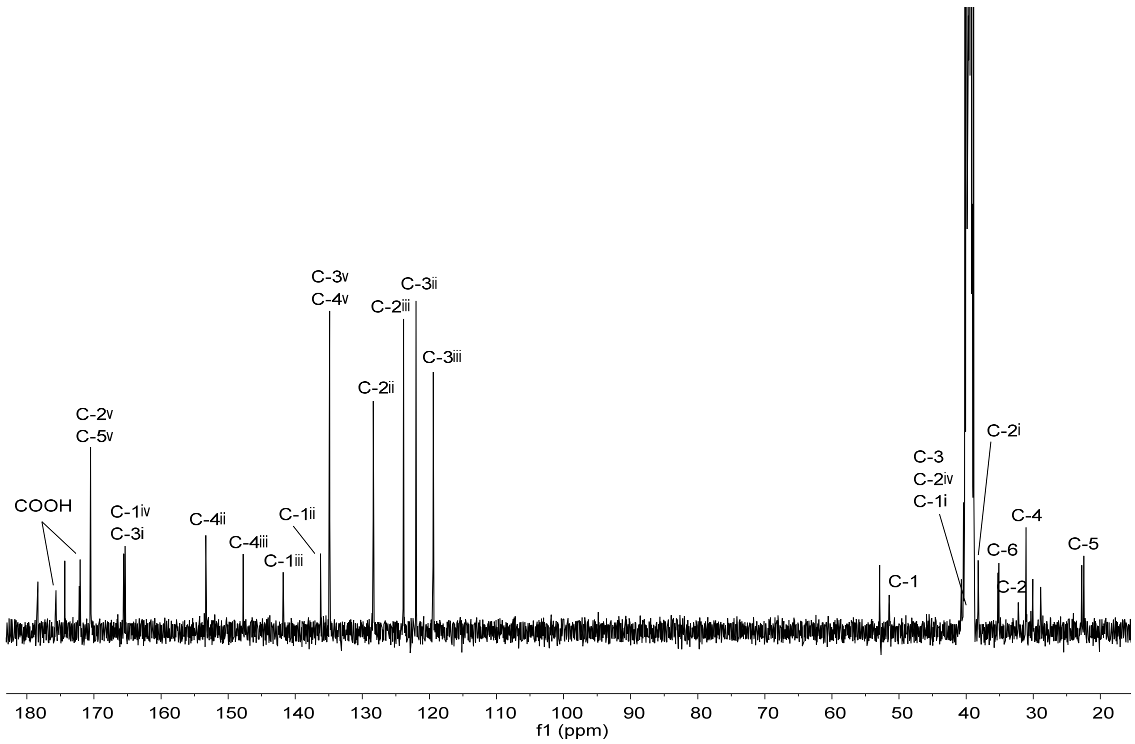

^{13}**C NMR** (100.6 MHz,DMSO-d$_6$, 310 K)

Supplementary Figure 34 ^{1}H and ^{13}C NMR spectra of compound **MAG$_{2P}^{slow}$.** In the presence of water, we observed two sets of signals in the ^{13}**C NMR** corresponding to the glutamate moiety. This is due to an acid-base equilibrium of the amino and carboxylic groups.

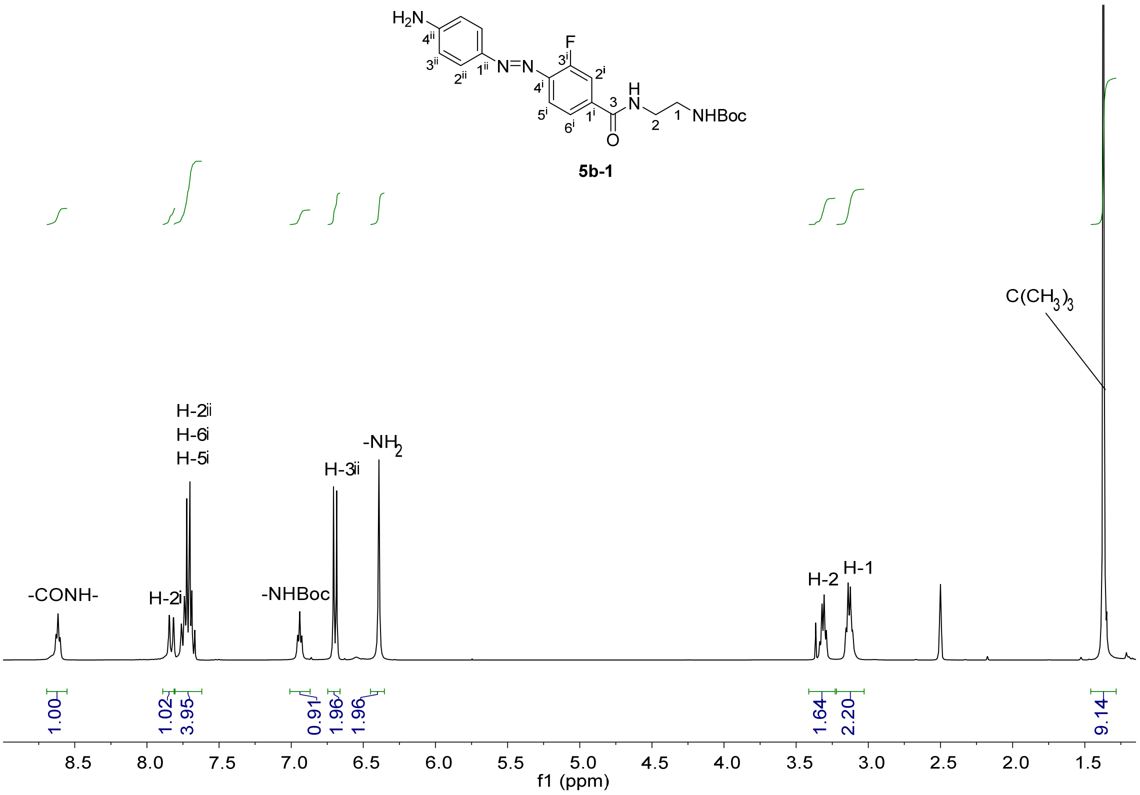

^{1}**H NMR** (400 MHz, DMSO-d$_6$)

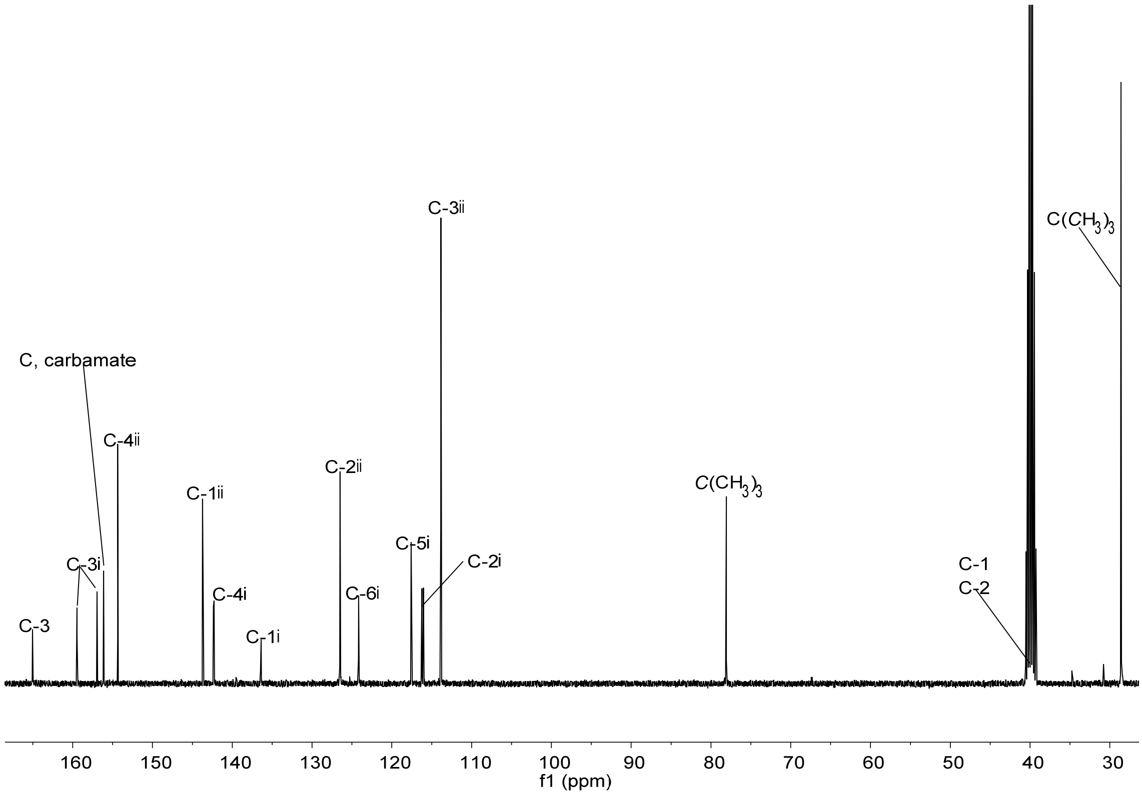

^{13}**C NMR** (100.6 MHz, DMSO-d$_6$)

Supplementary Figure 35 ^{1}H and ^{13}C NMR spectra of compound **5b-1.**

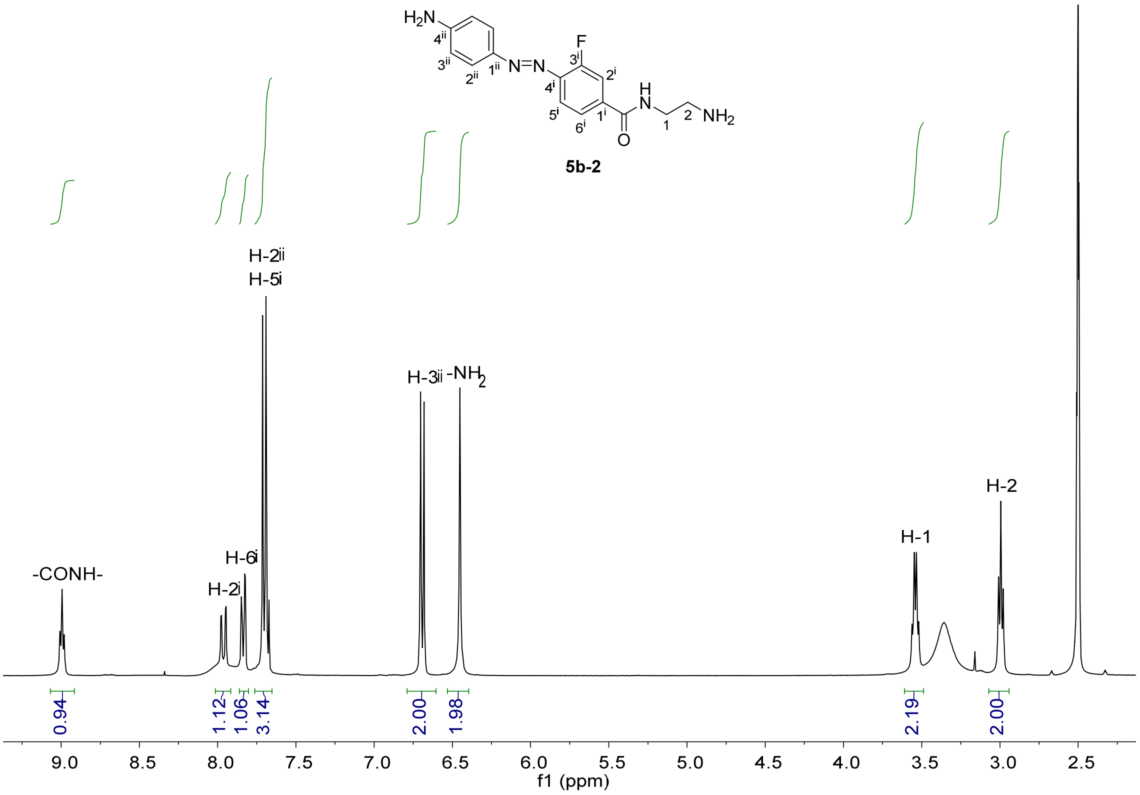

^{1}**H NMR** (400 MHz, DMSO-d$_6$)

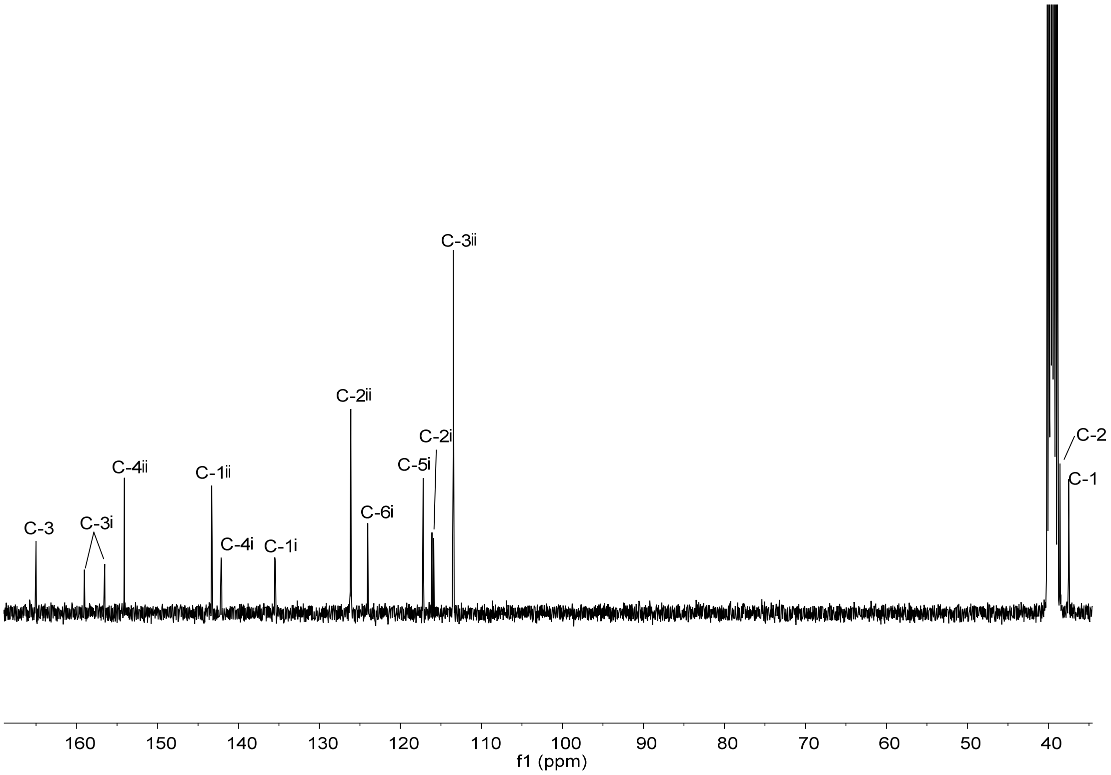

^{13}**C NMR** (100.6 MHz, DMSO-d$_6$)

Supplementary Figure 36 ^{1}H and ^{13}C NMR spectra of compound **5b-2.**

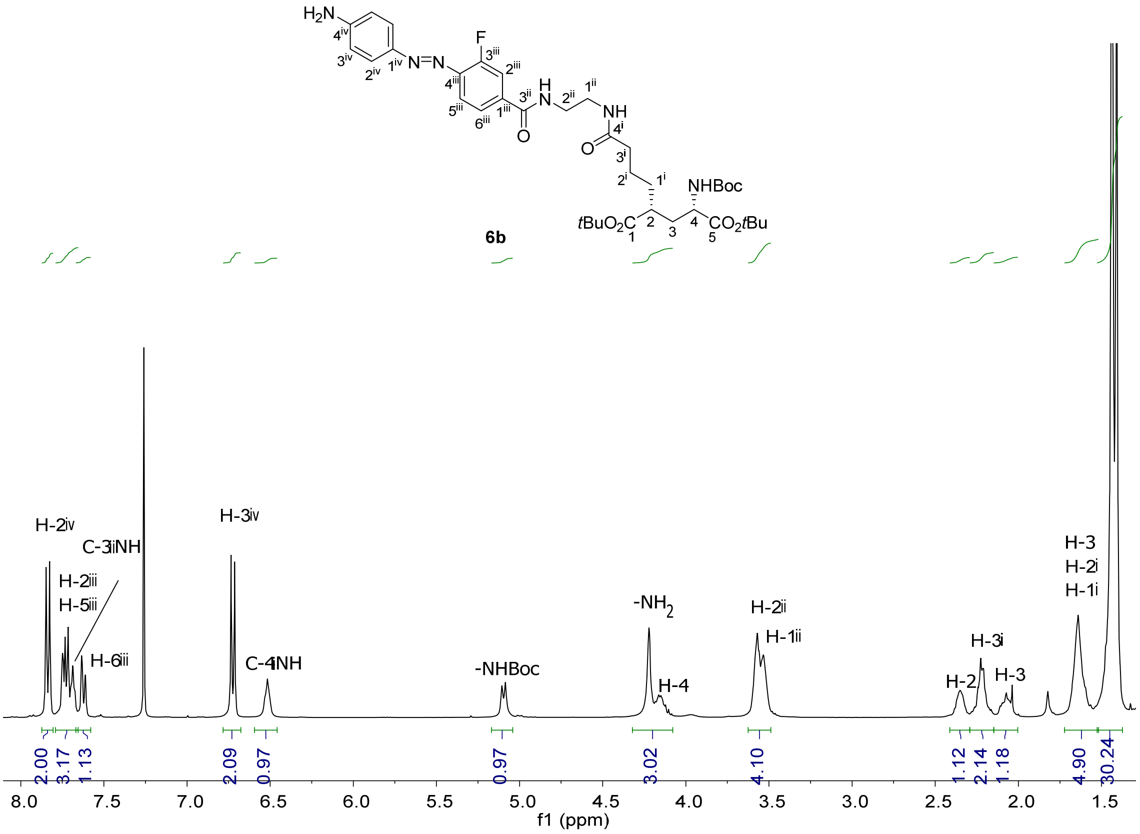

^{1}H NMR (400 MHz, $CDCl_3$)

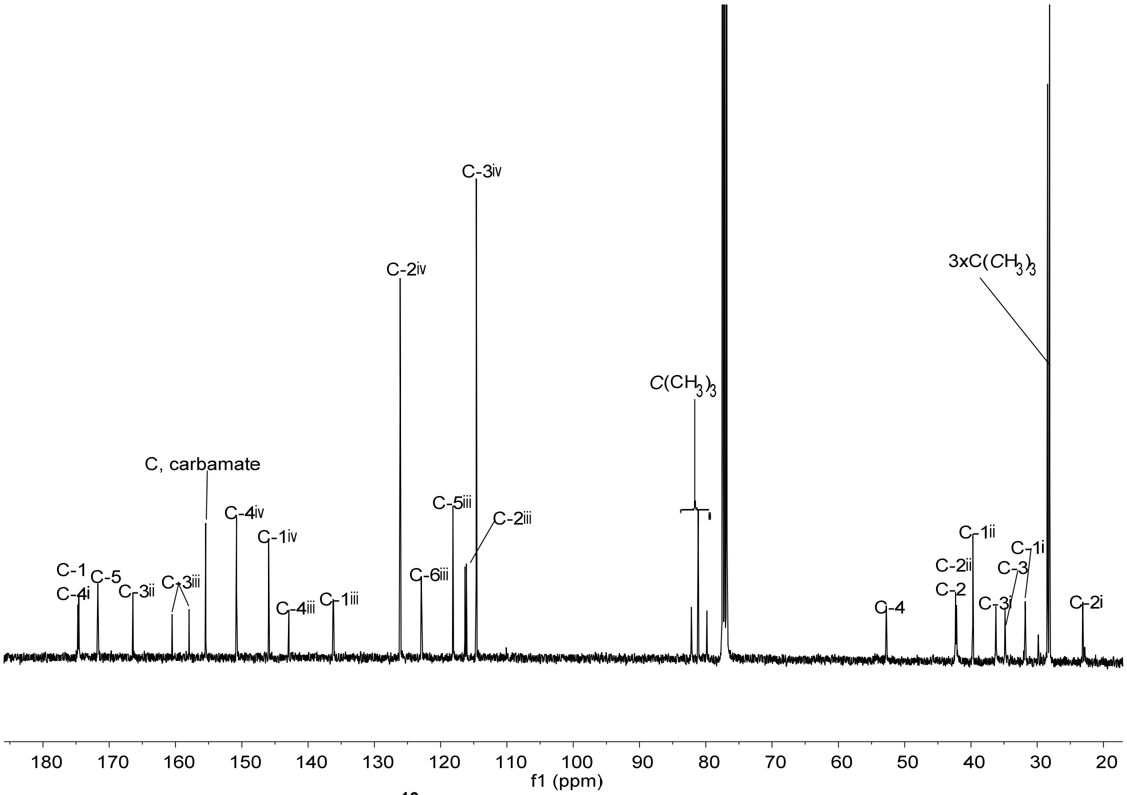

^{13}C NMR (100.6 MHz,$CDCl_3$)

Supplementary Figure 37 ^{1}H and ^{13}C NMR spectra of compound **6b.**

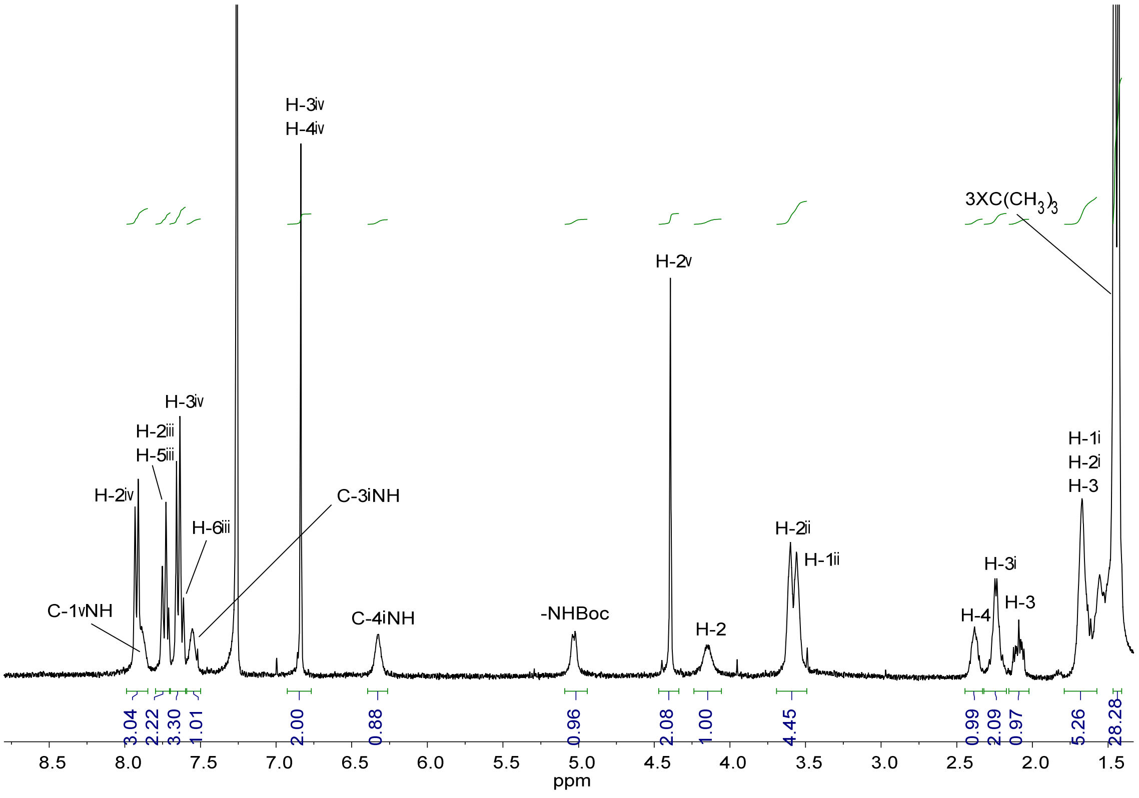

^{1}H NMR (400 MHz, $CDCl_3$)

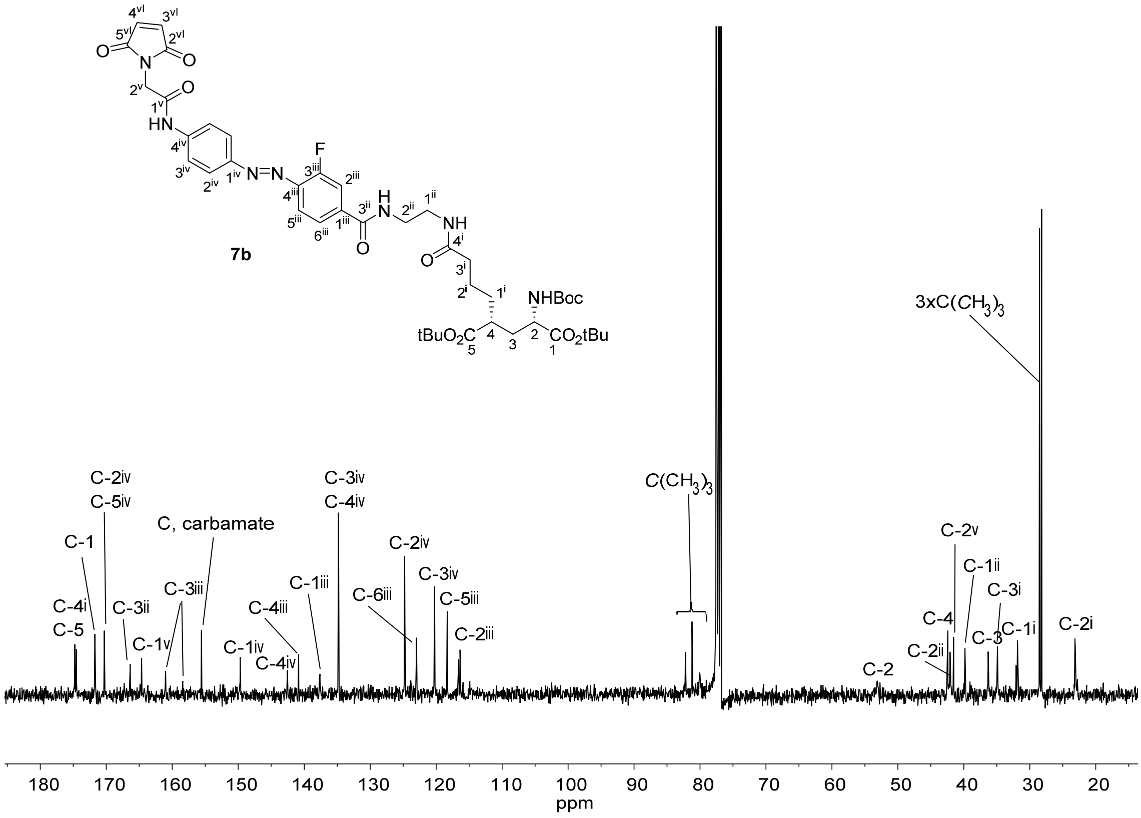

^{13}C NMR (100.6 MHz, $CDCl_3$)

Supplementary Figure 38 ^{1}H and ^{13}C NMR spectra of compound **7b.**

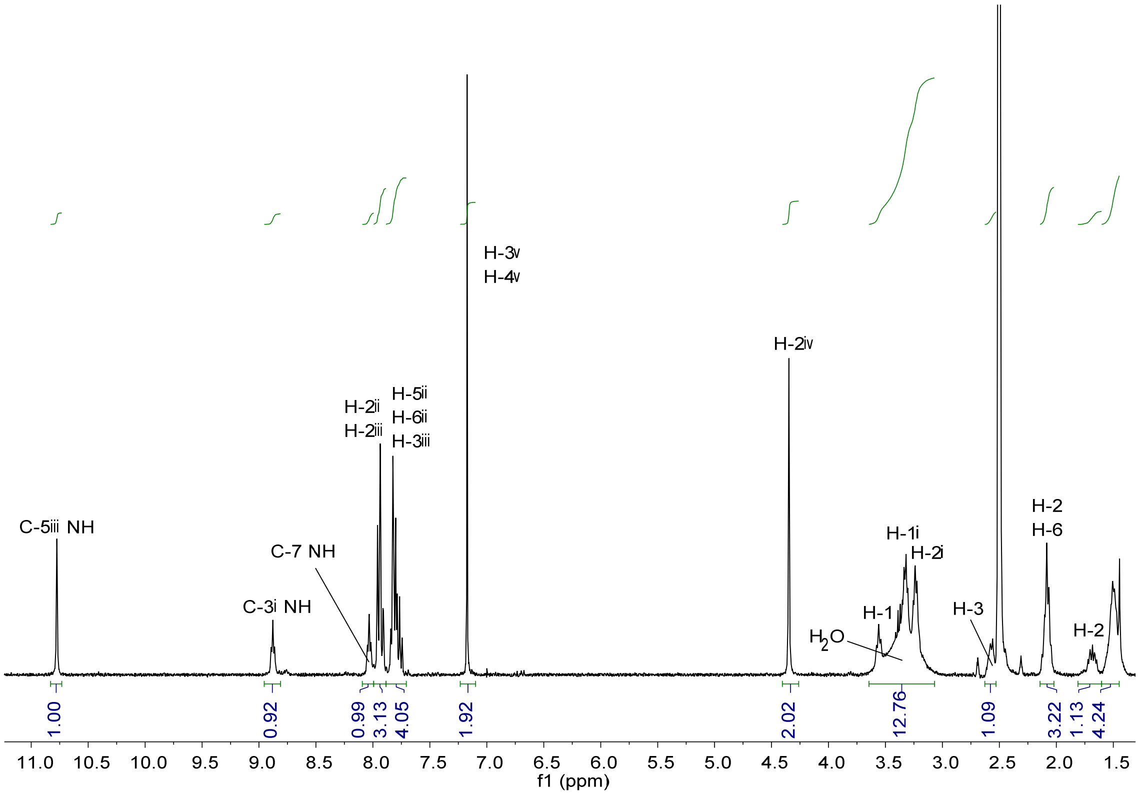

^{1}H NMR (400 MHz, DMSO-d$_6$, 310 K)

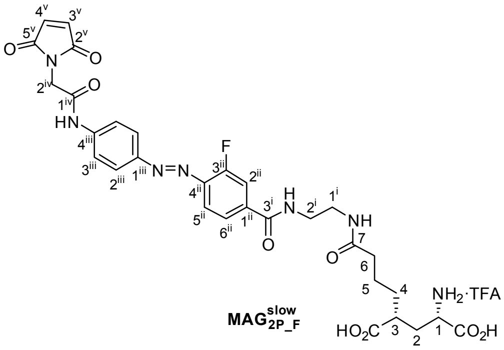

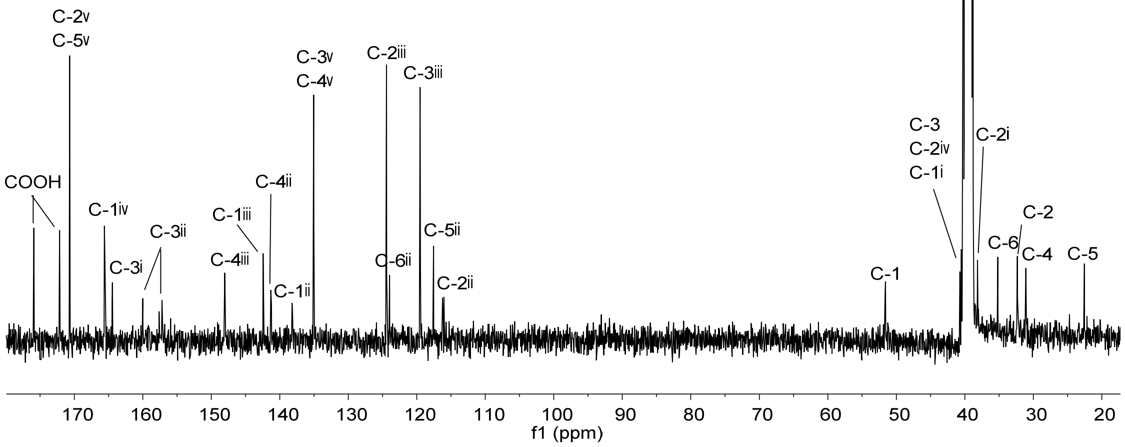

^{13}C NMR (100.6 MHz,DMSO-d$_6$, 310 K)

Supplementary Figure 39 ^{1}H and ^{13}C NMR spectra of compound $\mathbf{MAG^{slow}_{2P\_F}}$.

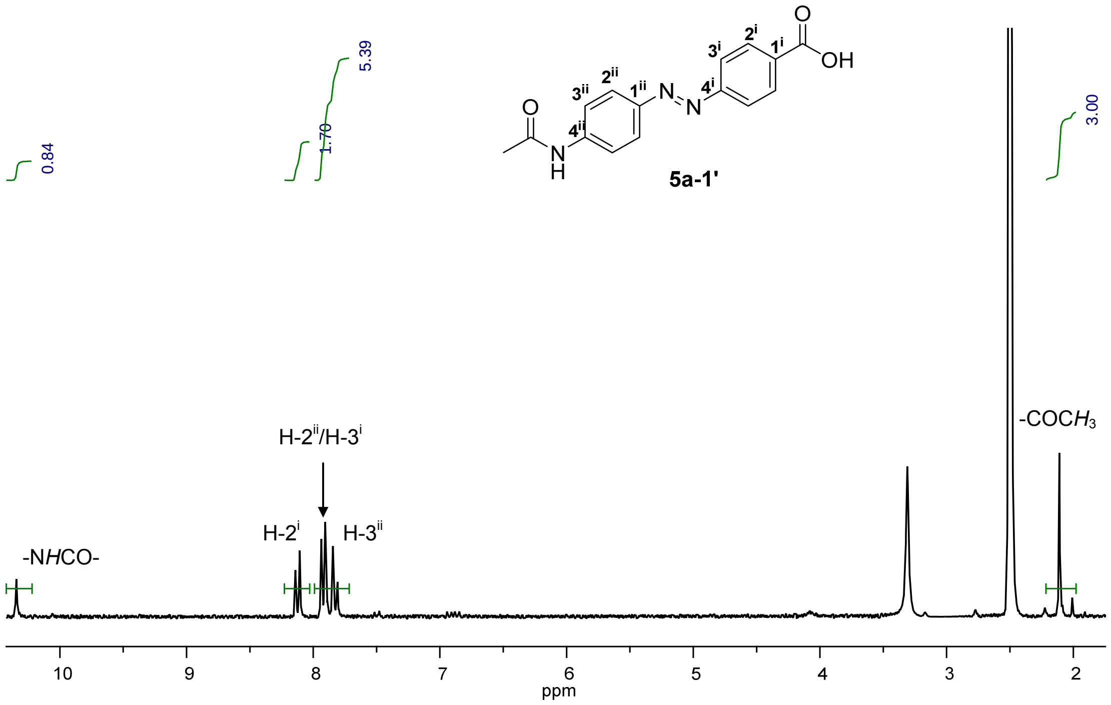

^{1}H NMR (250 MHz, DMSO-d$_6$)

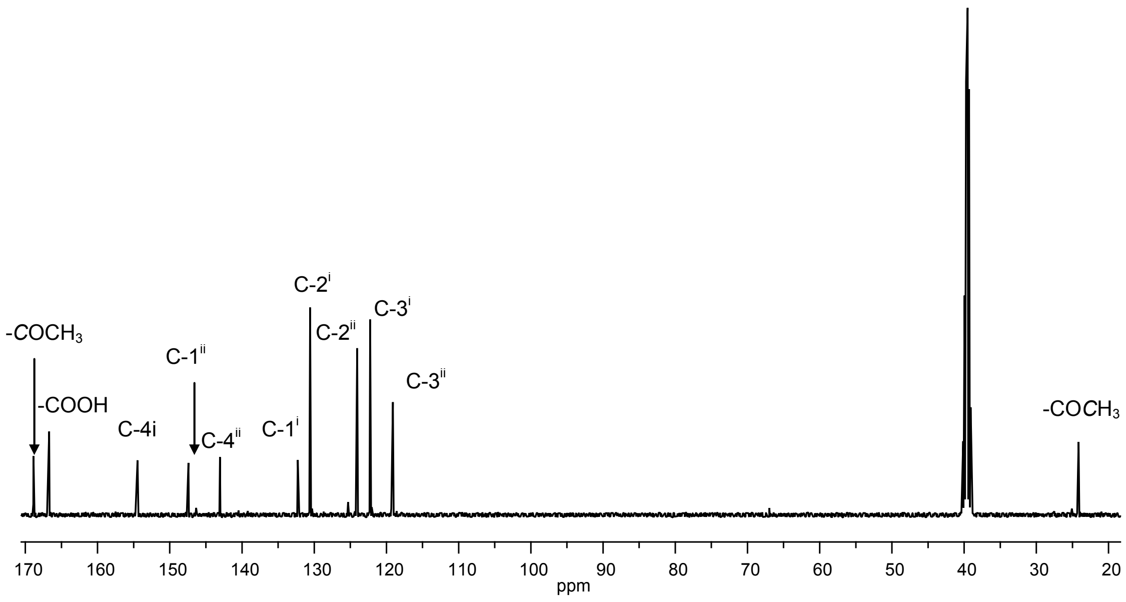

^{13}C NMR (100.6 MHz, DMSO-d$_6$)

Supplementary Figure 40 ^{1}H and ^{13}C NMR spectra of compound **5a-1’.**

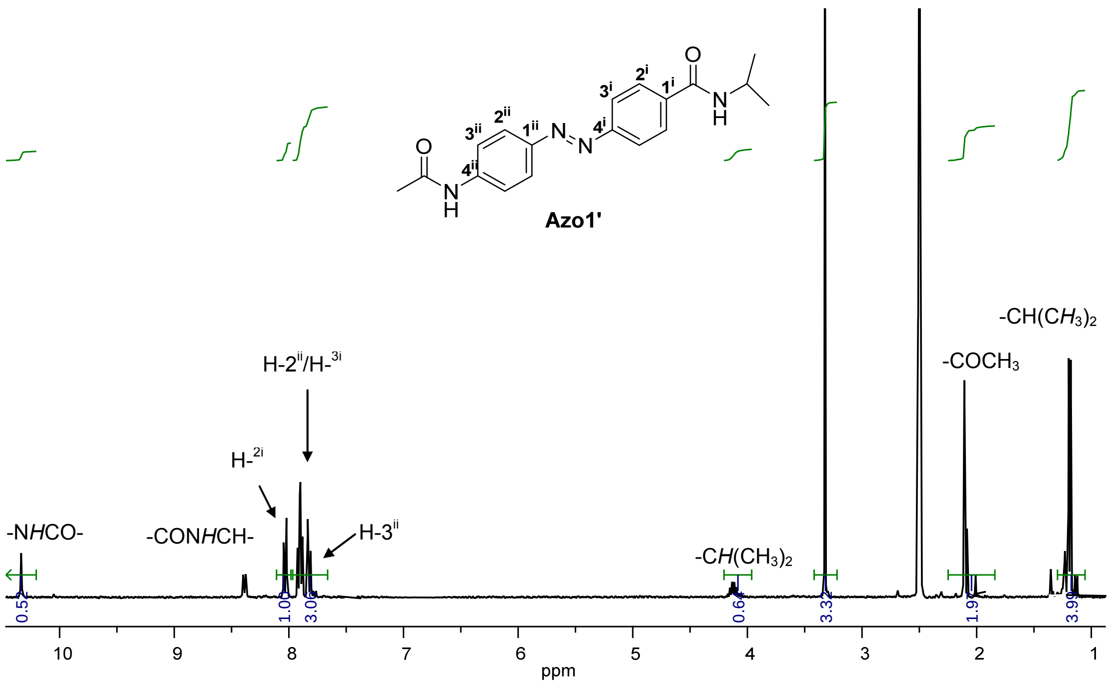

^{1}H RMN (360 MHz, DMSO-d_6)

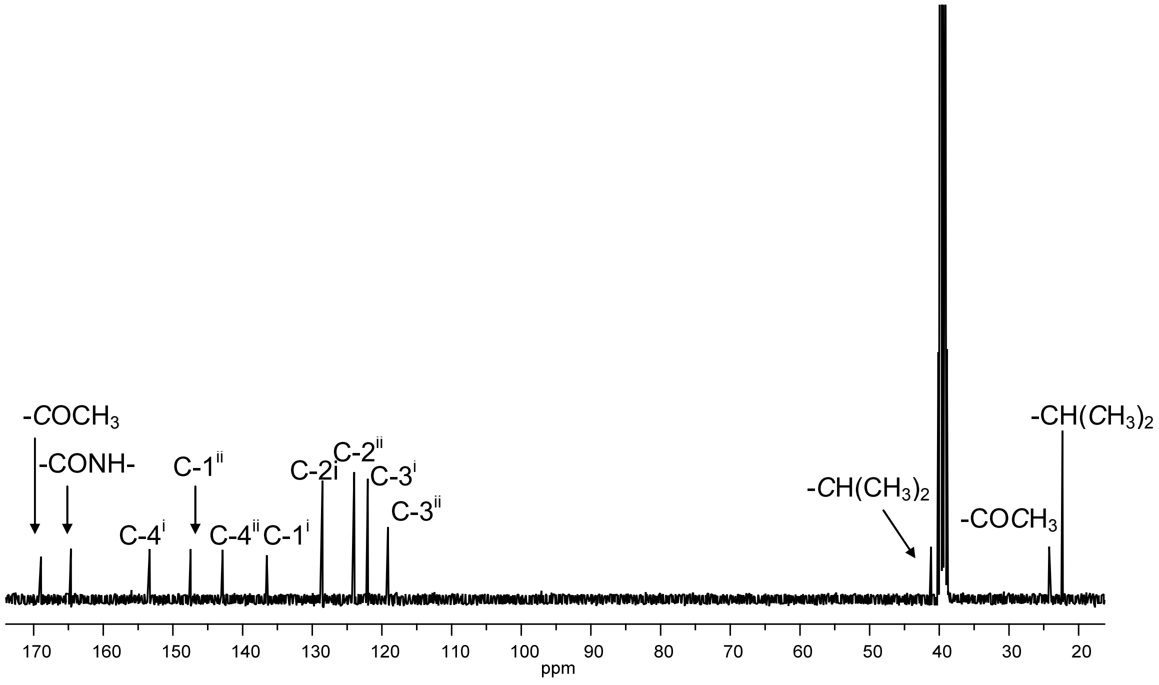

^{13}C NMR (100.6 MHz, DMSO-d_6)

Supplementary Figure 41 ^{1}H and ^{13}C NMR spectra of compound **Azo1'.**

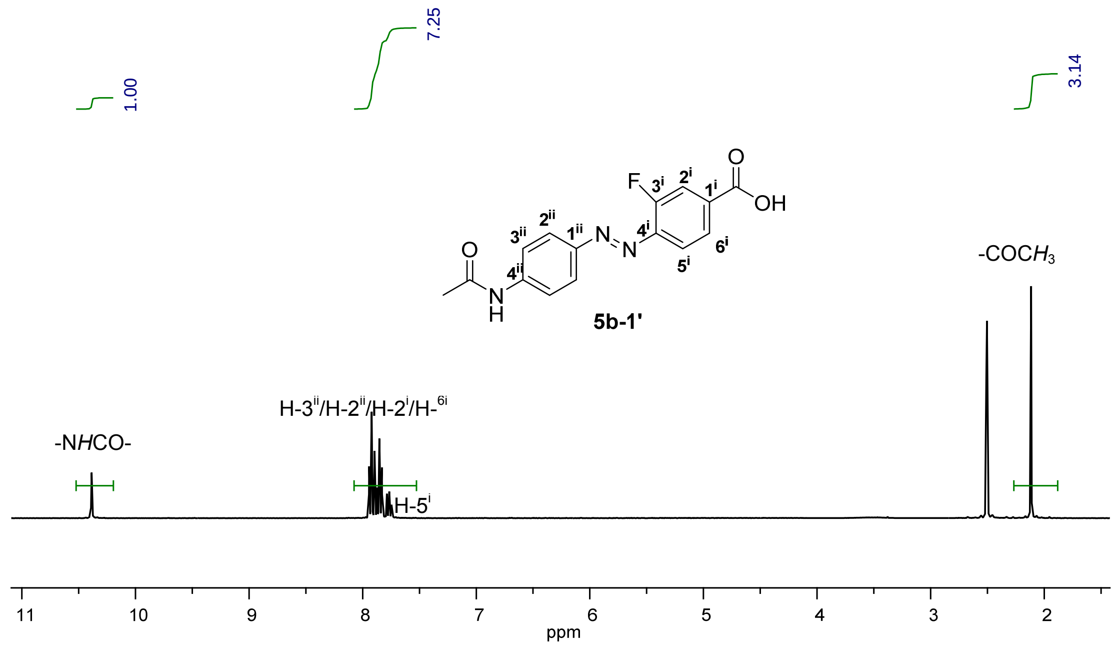

^{1}H NMR (400 MHz, DMSO-d_6)

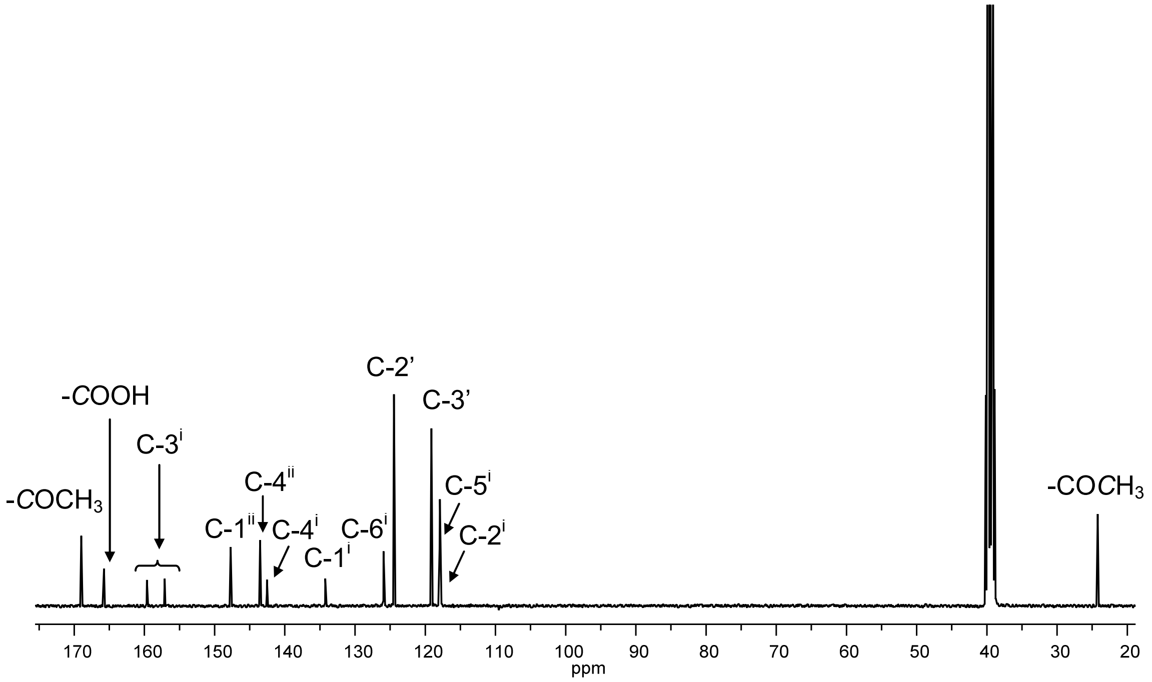

^{13}C NMR (100.6 MHz, DMSO-d_6)

Supplementary Figure 42 ^{1}H and ^{13}C NMR spectra of compound **5b-1’.**

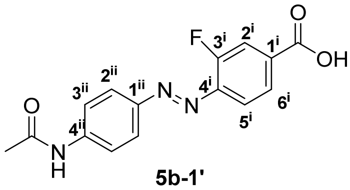

5b-1'

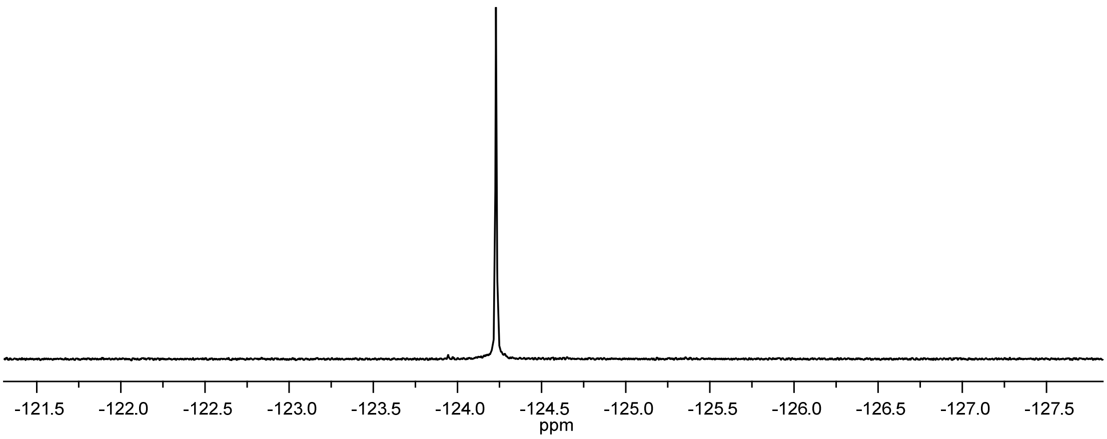

^{19}F NMR (400 MHz, DMSO-d_6)

Supplementary Figure 43 ^{19}F NMR spectrum of compound **5b-1'.**

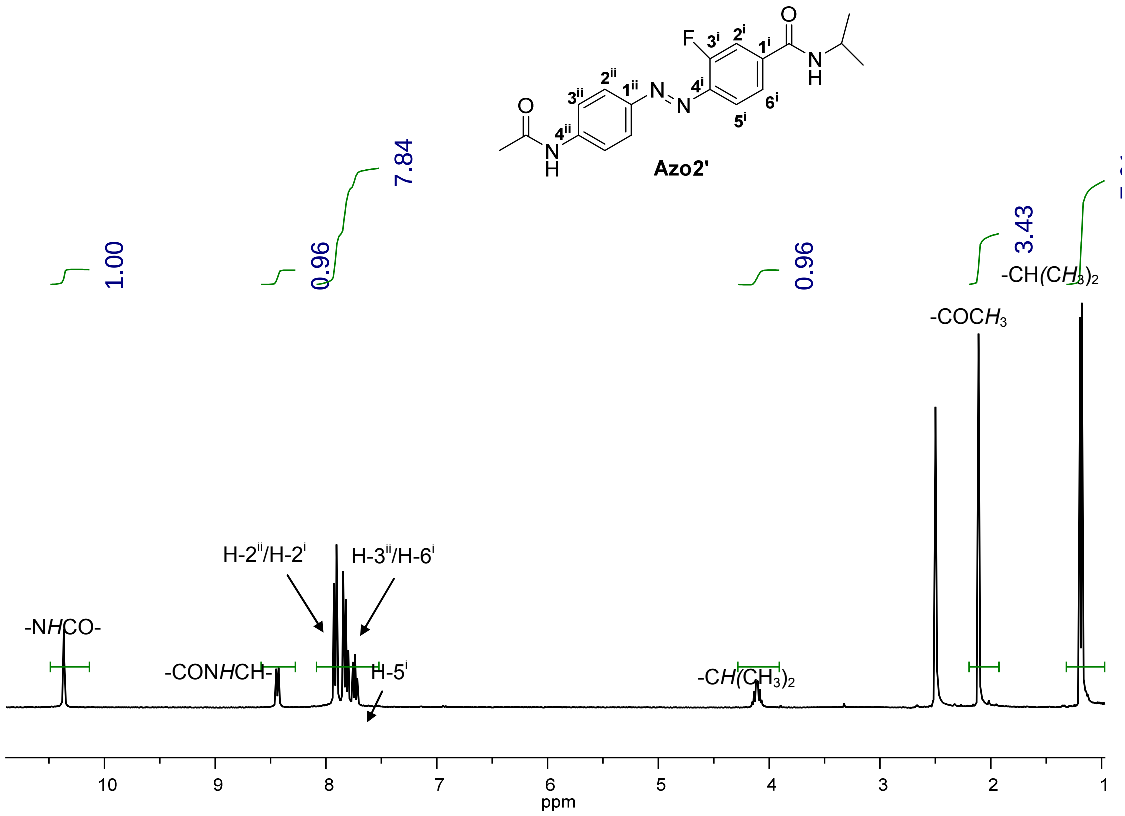

¹H NMR (400 MHz, DMSO-d$_6$)

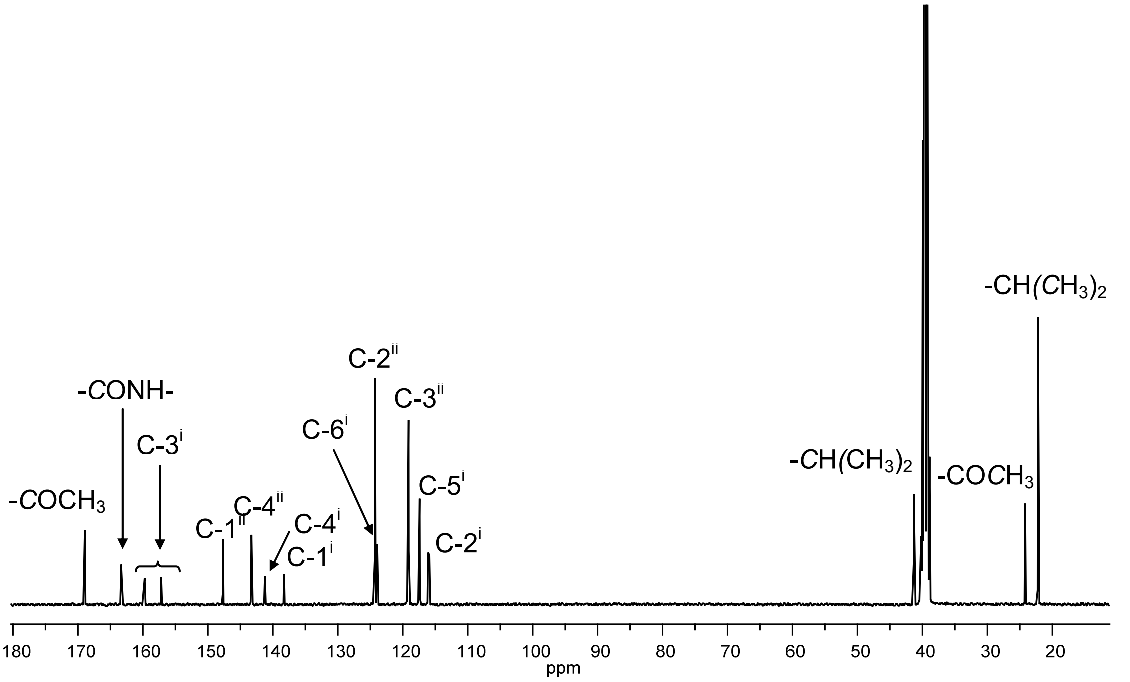

¹³C NMR (100.6 MHz, DMSO-d$_6$)

Supplementary Figure 44 ^{1}H and ^{13}C NMR spectra of compound **Azo2'.**

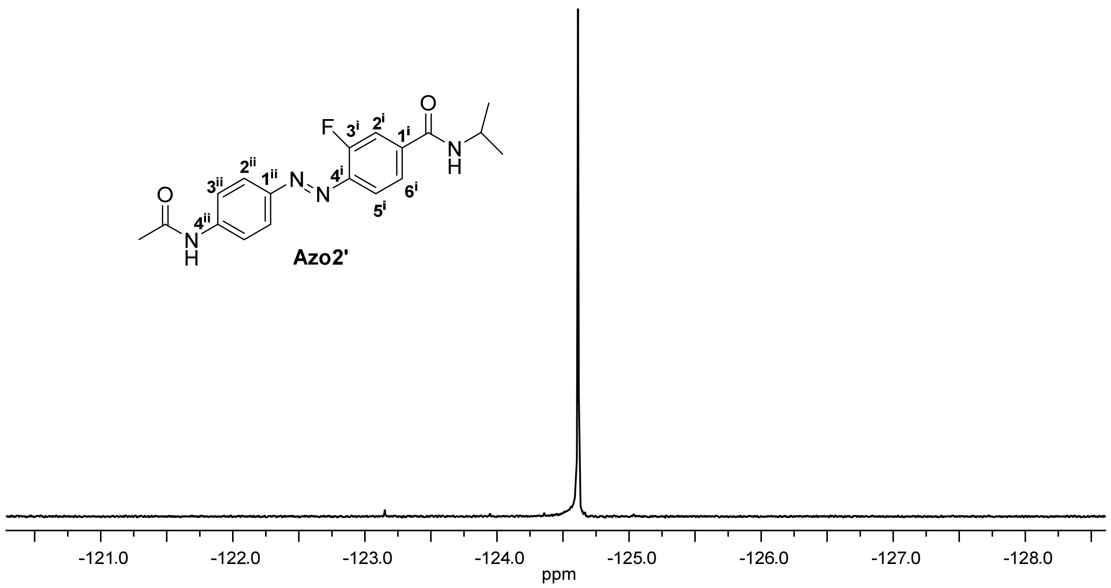

^{19}F RMN (400 MHz, DMSO-d_6)

Supplementary Figure 45 ^{19}F NMR spectrum of compound **Azo2'.**

Supplementary Tables

Supplementary Table 1. Theoretical results in gas phase at the CAM-B3LYP/6-31G(d) level.

	DFT [a]		TDDFT [b]					
	$\Delta E_{trans\text{-}cis}$	$\Delta E^{\ddagger}_{cis\text{-}trans}$ [c]	$S_0 \rightarrow S_1$ transition			$S_0 \rightarrow S_2$ transition		
	(kJ mol^{-1})		E_{exc} (eV)	f	σ_2	E_{exc} (eV)	f	σ_2
***trans*-Azo^{MAG}**	67.3	113.1	2.77	0.0	0.0	3.79	1.2	0
***cis*-Azo^{MAG}**			2.69	0.0	0.1	4.43	0.3	10
***trans*-Azo^{MAG2p}**	69.9	108.2	2.83	0.0	0.0	3.62	1.2	19
***cis*-Azo^{MAG2p}**			2.66	0.01	0.1	4.17	0.4	40
***trans*-Azo1**	64.9	95.4	2.71	0.0	0.0	3.87	1.2	19
***cis*-Azo1**			2.72	0.0	0.1	4.43	0.4	38
***trans*-Azo2**	53.1	94.7	2.71	0.0	0.0	3.98	1.0	21
***cis*-Azo2**			2.75	0.0	0.1	4.42	0.2	42
***trans*-Azo3**	49.2	97.3	2.73	0.0	0.0	4.00	1.1	27
***cis*-Azo3**			2.82	0.0	0.1	4.38	0.4	39
***trans*-Azo4**	64.7	46.8	2.78	0.0	0.0	3.89	0.9	32
***cis*-Azo4**			2.84	0.0	0.6	3.85	0.1	14
***trans*-Azo5**	33.9	111.5	2.64	0.1	0.0	4.22	1.2	18
***cis*-Azo5**			2.87	0.0	0.1	4.41	0.2	34

[a] The difference in energy between the *cis* and *trans* isomers ($\Delta E_{trans\text{-}cis}$) and the minimum barrier height for the thermal *cis*→*trans* isomerization are given ($\Delta E^{\ddagger}_{cis\text{-}trans}$). [b] The excitation energy (E_{exc}), the oscillator strength of the 1P absorption process (f), and the absorption cross-section of the 2P absorption process (σ_2, in GM units) are given for the both isomers of each compound. [c] In all the cases, the lowest-energy barrier height for the thermal *cis*→*trans* isomerization was found to correspond to an inversion mechanism.

Supplementary Table 2. Theoretical results in water at the CAM-B3LYP/6-31G(d) level.

	DFT [a]		TDDFT [b]					
	$\Delta E_{trans\text{-}cis}$	$\Delta E^{\ddagger}_{cis\text{-}trans}$ [c]	$S_0 \rightarrow S_1$ transition			$S_0 \rightarrow S_2$ transition		
	(kJ mol^{-1})		E_{exc} (eV)	f	σ_2	E_{exc} (eV)	f	σ_2
***trans*-Azo^{MAG}**	58.3	117.4	2.77	0.0	0.0	3.44	1.4	0
***cis*-Azo^{MAG}**			2.71	0.0	0.3	4.20	0.4	23
***trans*-Azo^{MAG2p}**	61.4	nd [d]	2.85	0.0	0.0	3.15	1.4	56
***cis*-Azo^{MAG2p}**			2.67	0.1	- [e]	3.82	0.5	- [f]
***trans*-Azo1**	56.0	97.2	2.71	0.0	0.0	3.54	1.4	58
***cis*-Azo1**			2.75	0.0	0.4	4.22	0.5	81
***trans*-Azo2**	46.1	98.1	2.73	0.0	0.0	3.68	1.2	69
***cis*-Azo2**			2.77	0.0	0.5	4.19	0.3	91
***trans*-Azo3**	44.1	101.0	2.74	0.1	0.1	3.67	1.4	82
***cis*-Azo3**			2.78	0.0	0.5	4.11	0.6	97
***trans*-Azo4**	57.7	46.0	2.81	0.1	0.5	3.58	1.3	110
***cis*-Azo4**			2.85	0.1	1.8	3.78	0.1	32
***trans*-Azo5**	34.4	112.0	2.65	0.1	0.0	3.74	1.5	51
***cis*-Azo5**			2.85	0.0	0.8	4.16	0.3	85

[a] The difference in energy between the *cis* and *trans* isomers ($\Delta E_{trans\text{-}cis}$) and the barrier height for the thermal *cis*→*trans* isomerization are given ($\Delta E^{\ddagger}_{cis\text{-}trans}$). [b] The excitation energy (E_{exc}), the oscillator strength of the 1P absorption process (f), and the absorption cross-section of the 2P absorption process (σ_2, in GM units) are given for both isomers of each compound. [c] In all the cases, the lowest-energy barrier height for the thermal *cis*→*trans* isomerization was found to correspond to an inversion mechanism. [d] Accurate description of the thermal isomerization barrier height would require inclusion of explicit water molecules in the calculation. [e] Calculation did not converge. [f] Calculation did not converge. From the σ_2 value computed in the gas phase and the equations given in reference 4 for the solvent dependence of σ_2, an estimate of the 2P absorption cross-section in water was made (σ_2 = 100 GM).

Supplementary Table 3. *Trans-cis* photoisomerization of $\mathbf{MAG_{2P}^{slow}}$ and $\mathbf{MAG_{2P\_F}^{slow}}$.

	DMSO			99% PBS : 1% DMSO		
	$\lambda^{abs}_{max,trans}$ (nm)	%*cis* PSS	$\Phi_{trans\text{-}cis}$	$\lambda^{abs}_{max,trans}$ (nm)	%*cis* PSS	$\Phi_{trans\text{-}cis}$
$\mathbf{MAG_{2P}^{slow}}$	367	72	0.20	351	71	0.13
$\mathbf{MAG_{2P\_F}^{slow}}$	376	68	0.27	358	69	0.16
**Azo1'** [a]	369	75	0.25	nd	nd	nd
**Azo2'** [a]	378	66	0.29	nd	nd	nd
**MAG**	nd	nd	nd	361	70	0.18

[a] The photochemical properties of *trans*-**Azo1'** and *trans*-**Azo2'** were not determined in 99% PBS:1% DMSO because of the low solubility of these compounds in aqueous media.

Supplementary Table 4. *Cis-trans* photoisomerization of $\mathbf{MAG_{2P}^{slow}}$ and $\mathbf{MAG_{2P\_F}^{slow}}$.

	DMSO		99% PBS : 1% DMSO	
	%*trans* PSS	$\Phi_{cis\text{-}trans}$	%*trans* PSS	$\Phi_{cis\text{-}trans}$
$\mathbf{MAG_{2P}^{slow}}$	78	0.77	88	0.26
$\mathbf{MAG_{2P\_F}^{slow}}$	69	0.81	80	0.26
**Azo1'**	75	0.79	nd	nd
**Azo2'**	63	0.62	nd	nd
**MAG**	nd	nd	91	0.30

[a] The photochemical properties of *cis*-**Azo1'** and *cis*-**Azo2'** were not determined in 99% PBS:1% DMSO because of the low solubility of these compounds in aqueous media.

Supplementary Table 5. Thermal stability of *cis*-$\mathbf{MAG_{2P}^{slow}}$ and *cis*-$\mathbf{MAG_{2P\_F}^{slow}}$ at rt.

	τ_{cis} (h) in DMSO	τ_{cis} (min) in 99% PBS:1% DMSO
$\mathbf{MAG_{2P}^{slow}}$	10	10.5
$\mathbf{MAG_{2P\_F}^{slow}}$	23	11.9
**Azo1'**	22.3	nd
**Azo2'**	29.3	nd
**MAG**	nd	25.5 [b]

[a] The τ_{cis} values of **Azo1'** and **Azo2'** were not determined in 99% PBS:1% DMSO because of the low solubility of these compounds in aqueous media. [b] From reference 1.

Supplementary Table 6. Activation and deactivation lifetimes (τ_{ON}, τ_{OFF}).[a]

	τ_{ON} (s)	τ_{OFF} (s)	*N* (cells)
**MAG**	0.16 ± 0.01	0.22 ± 0.01	4
$\mathbf{MAG_{2P}^{slow}}$	0.31 ± 0.02	0.35 ± 0.038	5
$\mathbf{MAG_{2P\_F}^{slow}}$	0.26 ± 0.03	0.27 ± 0.01	3

[a] Activation and deactivation lifetimes (τ_{ON}, τ_{OFF}) of GluK2-L439C conjugated to **MAG**, $\mathbf{MAG_{2P}^{slow}}$, and $\mathbf{MAG_{2P\_F}^{slow}}$. The number of recordings from different cells used for averaging is indicated. In each recording, the calculated lifetimes were averaged from four photoswitching cycles (see Supplementary Figure 10).

Supplementary Table 7. Primers used for Gibson assembly in the construction of pNMSB18.

	Primer sequence	Anneals
**FW**	GATAACATGGCAATTATTAAAGAGTTTATG	mCherry
**RV**	GATCGAATCGTCTCACAACTGATCC	mec-17p
**FW**	AGTTGTGAGACGATTCGATCATGAAGATTATTTCCCCAGTTTTAAG	LiGluR6Q
**RV**	TTAATAATTGCCATGTTATCGGTTTCTTTACCTGGCAAC	LiGluR6Q

Supplementary Methods

General procedure for the synthesis of azobenzene-based photoswitches: The preparation of ligands $\mathbf{MAG_{2P}^{slow}}$ and $\mathbf{MAG_{2P\_F}^{slow}}$ was achieved via a multistep synthetic sequence (see Figure 2 in the manuscript). In both cases, we took the corresponding aminobenzoic acid (**4a** for $\mathbf{MAG_{2P}^{slow}}$ and **4b** for $\mathbf{MAG_{2P\_F}^{slow}}$) to form the azobenzene core (**5a** for $\mathbf{MAG_{2P}^{slow}}$ and **5b** for $\mathbf{MAG_{2P\_F}^{slow}}$), to which the different functional fragments of the target compounds were sequentially introduced: fully protected glutamate derivative **2**[5] and maleimide acetic acid **3**.[6] In addition, azobenzene model compounds **Azo1'** and **Azo2'** were prepared as references for the photochemical characterization of $\mathbf{MAG_{2P}^{slow}}$ and $\mathbf{MAG_{2P\_F}^{slow}}$, respectively.

Materials and methods for the synthesis of azobenzene-based photoswitches: Commercially available reagents were used as received. Solvents were dried by distillation over the appropriate drying agents. All reactions were monitored by analytical thin-layer chromatography (TLC) using silica gel 60 precoated aluminum plates (0.20 mm thickness). Flash column chromatography was performed using silica gel (230-400 mesh). ^{1}H NMR and ^{13}C NMR spectra were recorded at 250, 360 or 400 MHz and 90.5 or 100.6 MHz, respectively. Proton chemical shifts are reported in ppm (δ) ($CDCl_3$, δ 7.26 or DMSO-d_6, δ 2.50). Carbon chemical shifts are reported in ppm (δ) ($CDCl_3$, δ 77.16 or DMSO-d_6, δ 39.5). NMR signals were assigned with the help of COSY, HSQC, HMBC and DEPT135. Infrared peaks are reported in cm^{-1}. Melting points were determined on hot stage and are uncorrected. HRMS were recorded. Optical rotations were measured at 22 ± 2 °C.

***tert*-Butyl [2-({4-[(*E*-4-aminophenyl)diazenyl]benzoyl}amino)ethyl]carbamate, 5a-1 (Supplementary Figure 15).** To a solution of acid **5a**[7] (553 mg, 2.29 mmol) in anhydrous THF (110 mL), a solution of HOBt (493 mg, 3.54 mmol), EDCI (0.58 mL, 3.22 mmol), DIPEA (1.73 mL, 9.88 mmol) and *tert*-butyl (2-aminoethyl)carbamate (0.48 mL, 2.98 mmol) in anhydrous THF (55 mL) was added. The reaction mixture was stirred overnight at rt. The solution was washed with H_2O (2 x 80 mL) and the resulting aqueous extracts were extracted with CH_2Cl_2 (3 x 80 mL). The combined organic extracts were dried over anhydrous Na_2SO_4 and concentrated under vacuum. The resulting residue was purified by column chromatography (CH_2Cl_2/THF, 4:1) to obtain **5a-1** as an orange solid (861 mg, 2.25 mmol, 98% yield): **Mp**: 203-205 ºC (from CH_2Cl_2/THF); **^{1}H NMR** (250 MHz, DMSO-d_6) δ 8.56 (br t, $J_{CONH,2}$ = 5.4 Hz, 1H, CONH), 7.98 (d, $J_{2^i,3^i}$ = 8.5 Hz, 2H, H-2^i), 7.78 (d, $J_{3^i,2^i}$ = 8.5 Hz, 2H, H-3^i), 7.70 (d, $J_{2^{ii},3^{ii}}$ = 8.8 Hz, 2H, H-2^{ii}), 6.94 (br t, $J_{NHBoc,1}$ = 5.4 Hz, 1H, N*H*Boc), 6.69 (d, $J_{3^{ii},2^{ii}}$ = 8.8 Hz, 2H, H-

3^{ii}), 6.35 (s, 2H, N*H*$_2$), 3.31 (m, 2H, H-2), 3.14 (m, 2H, H-1), 1.38 (s, 9H, $C(CH_3)_3$); **^{13}C NMR** (100.6 MHz, DMSO-d$_6$) δ 165.8 (C-3), 155.7 (C=O, carbamate), 154.0 (C-4^{i}), 153.4 (C-4^{ii}), 142.9 (C-1^{ii}), 134.6 (C-1^{i}), 128.3 (C-2^{i}), 125.6 (C-2^{ii}), 121.3 (C-3^{i}), 113.4 (C-3^{ii}), 77.7 (*C*(CH$_3$)$_3$), 39.6 (C-1), 39.6 (C-2), 28.2 (C(*C*H$_3$)$_3$); **IR** (ATR) υ 3333, 2979, 2926, 1686, 1597, 1526, 1275, 1132 cm^{-1}. **HRMS** (EI-TOF) *m/z* calcd for $[C_{20}H_{25}N_5O_3]^+$ 383.1957; found 383.1949. **COSY**, **DEPT 135** and **^{1}H/^{13}C** correlation were recorded. See **Supplementary Figure 30** for the ^{1}H and ^{13}C NMR spectra.

(*E*)-*N*-(2-Aminoethyl)-4-((4-aminophenyl)diazenyl)benzamide, 5a-2 (Supplementary Figure 16). To a solution of carbamate **5a-1** (257 mg, 0.67 mmol) in MeOH (26 mL), HCl 37% (7.5 mL, 90.6 mmol) was added dropwise. The mixture was stirred at rt for 90 min until the complete consumption of the starting material. Afterwards, the mixture was neutralised with a saturated aqueous solution of $NaHCO_3$, extracted with CH_2Cl_2 (3 x 40 mL) and the combined organic extracts were dried over anhydrous Na_2SO_4. The solvent was evaporated under vacuum and the resulting residue was washed with Et_2O and filtered to furnish amine **5a-2** (100 mg, 0.35 mmol, 53% yield) as a dark orange solid: **Mp**: 150-156 ºC (from Et_2O); **^{1}H NMR** (250 MHz, DMSO-d$_6$) δ 8.49 (br t, $J_{CONH,1}$ = 5.3 Hz, 1H, CON*H*), 7.98 (d, $J_{2^i,3^i}$ = 8.3 Hz, 2H, H-2^{i}), 7.78 (d, $J_{3^i,2^i}$ = 8.3 Hz, 2H, H-3^{i}), 7.69 (d, $J_{2^{ii},3^{ii}}$ = 8.5 Hz, 2H, H-2^{ii}), 6.68 (d, $J_{3^{ii},2^{ii}}$ = 8.5 Hz, 2H, H-3^{ii}), 6.23 (br s, 2H, N*H*$_2$), 3.30 (m, 2H, H-1), 2.69 (t, $J_{2,1}$ = 6.4 Hz, 2H, H-2); **^{13}C NMR** (100.6 MHz, DMSO-d$_6$) δ 165.8 (C-3), 153.9 (C-4^{i}), 153.4 (C-4^{ii}), 142.9 (C-1^{ii}), 134.8 (C-1^{i}), 128.3 (C-2^{i}), 125.6 (C-2^{ii}), 121.3 (C-3^{i}), 113.4 (C-3^{ii}), 42.9 (C-1), 41.2 (C-2); **IR** (ATR) υ 3295, 2930, 1631, 1597, 1532, 1296, 1135 cm^{-1}. **HRMS** (EI-TOF) *m/z* calcd for $[C_{15}H_{17}N_5O]^+$ 283.1433; found 283.1445. **COSY**, **DEPT 135** and **^{1}H/^{13}C** correlation were recorded. See **Supplementary Figure 31** for the ^{1}H and ^{13}C NMR spectra.

di-*tert*-Butyl (2*R*,4*S*)-2-(4-{[2-({4-[(*E*)-(4-aminophenyl)diazenyl]benzoyl}amino)ethyl]amino}-4-oxobutyl)-4-[(*tert*-butoxycarbonyl)amino]pentanedioate, 6[a] (Supplementary Figure 17). To a solution of amine **5a-2** (85 mg, 0.30 mmol) in anhydrous THF (16 mL), a solution of glutamate **2** (149 mg, 0.33 mmol), HOBt (64 mg, 0.46 mmol), EDCI (72 µL, 0.40 mmol) and DIPEA (216 µL, 1.23 mmol) in anhydrous THF (11 mL) was added. The reaction mixture was stirred overnight at rt. The orange solution was washed with H_2O (2 x 25 mL) and the resulting aqueous extracts were extracted with CH_2Cl_2 (2 x 25 mL). The combined organic extracts were dried over anhydrous Na_2SO_4 and concentrated under vacuum. The resulting residue was purified by column chromatography (EtOAc) to give **6a** (192 mg, 0.27 mmol, 90% yield) as an orange solid: **Mp**: 75-78 ºC (from EtOAc); **$[\alpha]_D^{20}$** = 2.2 (*c* 0.50, $CHCl_3$); **^{1}H NMR** (400

MHz, $CDCl_3$) δ 7.92 (d, $J_{2^{iii},3^{iii}}$ = 8.6 Hz, 2H, H-2^{iii}), 7.84 (d, $J_{3^{iii},2^{iii}}$ = 8.6 Hz, 2H, H-3^{iii}), 7.80 (d, $J_{2^{iv},3^{iv}}$ = 8.7 Hz, 2H, H-2^{iv}), 7.58 (br t, $J_{NH,2^{ii}}$ = 4.4 Hz, 1H, CON*H*), 6.73 (d, $J_{3^{iv},2^{iv}}$ = 8.7 Hz, 2H, H-3^{iv}), 6.65 (t, $J_{NH,1^{ii}}$ = 5.0 Hz, 1H, CON*H*), 5.12 (d, $J_{NHBoc,4}$ = 8.6 Hz, 1H, N*H*Boc), 4.15 (m, 1H, H-4), 3.58 (m, 2H, H-2^{ii}), 3.52 (m, 2H, H-1^{ii}), 2.34 (m, 1H, H-2), 2.21 (m, 2H, H-3^{i}), 2.05 (m, 1H, H-3), 1.62 (m, 5H, H-3, H-1^{i}, H-2^{i}), 1.43 (s, 18H, 2xC(CH_3)$_3$), 1.41 (s, 9H, C(C*H*$_3$)$_3$); **^{13}C NMR** (100.6 MHz, $CDCl_3$) δ 174.6 (C-1, C-4^{i}), 171.7 (C-5), 167.8 (C-3^{ii}), 155.5 (C, carbamate), 154.9 (C-4^{iii}), 150.4 (C-4^{iv}), 145.6 (C-1^{iv}), 134.6 (C-1^{iii}), 128.1 (C-2^{iii}), 125.7 (C-2^{iv}), 122.5 (C-3^{iii}), 114.7 (C-3^{iv}), 82.1 (*C*(CH_3)$_3$), 81.1 (*C*(CH_3)$_3$), 79.8 (*C*(CH_3)$_3$), 52.8 (C-4), 42.4 (C-2), 41.8 (C-2^{ii}), 39.8 (C-1^{ii}), 36.2 (C-3^{i}), 34.8 (C-3), 32.0 (C-1^{i}), 28.4 (C(*C*H$_3$)$_3$), 28.2 (C(*C*H$_3$)$_3$), 28.1 (C(*C*H$_3$)$_3$), 23.2 (C-2^{i}); **IR** (ATR) υ 3345, 2976, 1706, 1637, 1599 cm^{-1}. **HRMS** (ESI-TOF) *m/z* calcd for $[C_{37}H_{54}N_6O_8 + H]^+$ 711.4076; found 711.4073. **COSY**, **DEPT 135** and **^{1}H/^{13}C** correlation were recorded. See **Supplementary Figure 32** for the ^{1}H and ^{13}C NMR spectra.

di-*tert*-Butyl (2*S*,4*R*)-2-[(*tert*-butoxycarbonyl)amino]-4-(4-{[2-({4-[(*E*)-(4-{[(2,5-dioxo -2,5-dihydro-1*H*-pyrrol-1-yl)acetyl]amino}phenyl)diazenyl]benzoyl}amino)ethyl] amino}-4-oxobutyl)pentanedioate, 7[a] (Supplementary Figure 18). To a stirred solution of maleimide acetic acid **3** (84 mg, 0.54 mmol) and oxalyl chloride 98% (49 μL, 0.56 mmol) in dry CH_2Cl_2 (8.4 mL) was added one drop of DMF. After stirring for 2 h at rt, the mixture was concentrated. The resulting acid chloride was taken up in dry THF (3.5 mL) and slowly added to an ice-cooled solution of **6a** (100 mg, 0.14 mmol), DIPEA (145 μL, 1.19 mmol) in dry THF (17.5 mL). After stirring for 10 min at this temperature, the mixture was warmed up to rt and stirred for further 4 h. Then, it was diluted with EtOAc (21 mL) and the resulting solution was washed with water (3 x 7 mL). The organic extract was dried over anhydrous Na_2SO4, concentrated under vacuum and purified by column chromatography (EtOAc/MeOH, 9:1) to provide **7a** (102 mg, 0.12 mmol, 86% yield) as an orange solid: **Mp**: 230 ºC (decomp. from EtOAc); **$[\alpha]_D^{20}$** = -42.2 (*c* 1.02, $CHCl_3$); **^{1}H NMR** (400 MHz, $CDCl_3$, 320 K) δ 8.36 (br s, 1H, CON*H*), 7.89 (d, $J_{2^{iii},3^{iii}}$ = 9.1 Hz, 2H, H-2^{iii}), 7.84 (m, 4H, H-3^{iii}, H-2^{iv}), 7.62 (d, $J_{3^{iv},2^{iv}}$ = 9.4 Hz, 2H, H-3^{iv}), 7.46 (t, $J_{NH,2^{ii}}$ = 4.7 Hz, 1H, CON*H*), 6.82 (s, 2H, H-3^{vi}, H-4^{vi}), 6.49 (t, $J_{NH,1^{ii}}$ = 4.9 Hz, 1H, CON*H*), 5.07 (d, $J_{NHBoc,2}$ = 8.4 Hz, 1H, N*H*Boc), 4.40 (s, 2H, H-2^{v}), 4.14 (m, 1H, H-2), 3.60 (m, 2H, H-2^{ii}), 3.55 (m, 2H, H-1^{ii}), 2.37 (m, 1H, H-4), 2.23 (m, 2H, H-3^{i}), 2.08 (m, 1H, H-3), 1.64 (m, 5H, H-3, 2xH-1^{i}, 2xH-2^{i}), 1.45 (s, 9H, C(C*H*$_3$)$_3$), 1.44 (s, 9H, C(C*H*$_3$)$_3$), 1.42 (s, 9H, C(C*H*$_3$)$_3$); **^{13}C NMR** (100.6 MHz, $CDCl_3$, 320 K) δ 174.6 (C-5, C-4^{i}), 171.7 (C-1), 170.3 (C-2^{vi}, C-5^{vi}), 167.7 (C-3^{ii}), 164.7 (C-1^{v}), 155.6 (C=O, carbamate), 154.5 (C-4^{iii}), 149.4 (C-4^{iv}), 140.5 (C-1^{iv}), 135.8 (C-1^{iii}), 134.8 (C-4^{vi}, C-3^{vi}), 128.2 (C-2^{iii}), 124.4 (C-2^{iv}), 123.0 (C-3^{iii}), 120.3 (C-3^{iv}), 82.1 (*C*(CH_3)$_3$), 81.1 (*C*(CH_3)$_3$),

79.8 (C(CH_3)$_3$), 53.1 (C-2), 42.5 (C-2), 41.8/41.5 (C-2^{ii}/C-2^{v}), 40.0 (C-1^{ii}), 36.4 (C-3^{i}), 34.9 (C-3), 32.0 (C-1^{i}), 28.5 (C(CH_3)$_3$), 28.3 (C(CH_3)$_3$), 28.2 (C(CH_3)$_3$), 23.2 (C-2^{i}); **IR** (ATR) υ 3277, 3081, 2926, 1714, 1543, 1147 cm^{-1}. **HRMS** (ESI-TOF) *m/z* calcd for [$C_{43}H_{57}N_7O_{11}$ + H]$^+$ 848.4189; found 848.4184. **DEPT 135** and **^{1}H/^{13}C** correlation were recorded. See **Supplementary Figure 33** for the ^{1}H and ^{13}C NMR spectra.

(1*S*,3*R*)-1,3-Dicarboxy-7-{[2-({4-[(*E*)-(4-{[(2,5-dioxo-2,5-dihydro-1*H*-pyrrol-1-yl)acetyl]amino}phenyl)diazenyl]benzoyl}amino)ethyl]amino}-7-oxo-1-heptanaminium trifluoroacetate, MAG$_{2P}^{slow}$ (Supplementary Figure 19). To a stirred solution of compound **7a** (85 mg, 0.10 mmol) in CH_2Cl_2 (15 mL), trifluoroacetic acid (7.5 mL, 97.9 mmol) was added. The mixture was stirred at rt until the starting material was consumed. Then, the solution was concentrated under vacuum and the resulting purple solid was washed with Et_2O to furnish **MAG$_{2P}^{slow}$** (60 mg, 0.08 mmol, 80% yield) as a lilac solid: **Mp** > 230 ºC (from Et_2O); **[α]$_D$^{20}** = -2.9 (*c* 0.51, DMSO); **^{1}H NMR** (400 MHz, DMSO-d$_6$, 310 K) δ 10.68 (s, 1H, CONH-1^{v}), 8.69 (br t, $J_{NH,2^i}$ = 5.6 Hz, 1H, CONH-3^{ii}), 8.04 (m, 2H, H-2^{ii}), 7.93 (m, 5H, H-2^{iii}, H-3^{ii}, CON*H*), 7.80 (m, 2H, H-3^{iii}), 7.15 (s, 2H, H-3^{v}, H-4^{v}), 4.34 (s, 2H, H-2^{iv}), 3.54 (m, 1H, H-1), 3.35 (m, 2H, H-2^{i}), 3.25 (m, 2H, H-1^{i}), 2.58 (m, 1H, H-3), 2.09 (m, 3H, H-2, 2xH-6), 1.69 (m, 1H, H-2), 1.51 (m, 4H, H-4, H-5); **^{13}C NMR** (100.6 MHz, DMSO-d$_6$, 310 K) δ 175.7/172.2/172.1 (2xCOOH/C-7), 170.5 (C-2^{v}, C-5^{v}), 165.6 (C-1^{iv}), 165.4 (C-3^{i}), 153.3 (C-4^{ii}), 147.8 (C-4^{iii}), 141.8 (C-1^{iii}), 136.2 (C-1^{ii}), 134.9 (C-3^{v}, C-4^{v}), 128.4 (C-2^{ii}), 123.9 (C-2^{iii}), 122.0 (C-3^{ii}), 119.4 (C-3^{iii}), 51.5 (C-1), 40.7 (C-3), 40.4 (C-2^{iv}), 38.2 (C-2^{i}), 38.1 (C-1^{i}), 35.3 (C-6), 32.2 (C-2), 31.1 (C-4), 22.5 (C-5); **IR** (ATR) υ 3280, 3089, 2922, 1711, 1545, 1431, 1143 cm^{-1}. **HRMS** (ESI-TOF) *m/z* calcd for [$C_{30}H_{34}N_7O_9$]$^+$ 636.2413; found 636.2400. **COSY**, **DEPT 135** and **^{1}H/^{13}C** correlation were recorded. See **Supplementary Figure 34** for the ^{1}H and ^{13}C NMR spectra.

4-[(*E*)-(4-Aminophenyl)diazenyl]-3-fluorobenzoic acid, 5b (Supplementary Figure 20). To an ice-cooled suspension of acid **4b** (1.50 g, 9.67 mmol) in HCl 5.5 M (6.0 mL), a solution of $NaNO_2$ (753 mg, 10.9 mmol) in H_2O (6 mL) was slowly added. The resulting mixture was added dropwise to an ice-cooled solution of anyline **1** (2.43 g, 11.6 mmol) in NaOAc 0.86 M (36 mL) and was stirred at 4 ºC overnight. The dark brown paste formed was filtered, washed with H_2O and the solid obtained was dissolved in NaOH 1 M (300 mL). The resulting solution was heated to 90 ºC for 2 h and, when cooled down, an orange precipitate appeared. It was filtered and recrystallised in NaOH 1 M. Afterwards, it was dissolved in the minimum quantity of H_2O and the solution was acidified to pH 2-3 with HCl 5.5 M. The orange precipitate obtained was filtered and washed with H_2O and Et_2O to afford **5b** (1.19 g, 4.59 mmol,

47% yield) as an orange solid: **Mp** > 265 ºC (decomp. from H_2O); **^{1}H NMR** (250 MHz, DMSO-d_6) δ 7.83 (m, 2H, H-2, H-6), 7.72 (m, 3H, 2xH-2^i, H-5), 6.69 (d, $_{3}i_{,2}i$ = 8.6 Hz, 2H, H-3^i), 6.45 (s, 2H, N*H*$_2$); **^{13}C NMR** (100.6 MHz, DMSO-d_6): δ 166.0 (d, $^4J_{C,F}$ = 2.3 Hz, COOH), 157.8 (d, $^1J_{C,F}$ = 253.5 Hz, C-3), 154.2 (C-4^i), 143.6 (C-1^i), 143.3 (d, $^2J_{C,F}$ = 7.2 Hz, C-4), 132.2 (d, $^3J_{C,F}$ = 7.2 Hz, C-1), 126.3 (C-2^i), 125.8 (d, $^4J_{C,F}$ = 3.3 Hz, C-6), 117.6 (C-5), 117.5 (d, $^2J_{C,F}$ = 21.1 Hz, C-2), 113.6 (C-3^i); **^{19}F{^{1}H} NMR** (400 MHz, DMSO-d_6) δ -125.3 (s, F); **IR** (ATR) υ 3391, 1678, 1595, 1387, 1295, 1141 cm^{-1}. **HRMS** (ESI-TOF) *m/z* calcd for $[C_{13}H_{10}FN_3O_2 + H]^+$ 258.0673; found 258.0675. **^{1}H/^{13}C** correlation were recorded.

***tert*-Butyl 2-({4-[(*E*)-(4-aminophenyl)diazenyl]-3-fluorobenzoyl}amino)ethyl carbamate, 5b-1 (Supplementary Figure 21).** To a solution of acid **5b** (200 mg, 0.77 mmol) in anhydrous THF (37 mL), a solution of HOBt (166 mg, 1.19 mmol), EDCI (0.20 mL, 1.11 mmol), DIPEA (0.58 mL, 3.31 mmol) and *tert*-butyl (2-aminoethyl)carbamate (0.16 mL, 0.99 mmol) in anhydrous THF (20 mL) was added. The reaction mixture was stirred overnight at rt. The solution was washed with H_2O (2 x 50 mL) and the resulting aqueous extracts were extracted with CH_2Cl_2 (2 x 30 mL). The combined organic extracts were dried over anhydrous Na_2SO_4 and concentrated under vacuum. The resulting residue was purified by column chromatography (CH_2Cl_2/THF, 4:1) to obtain **5b-1** (290 mg, 0.72 mmol, 94% yield) as an orange solid: **Mp**: 170-175 ºC (from CH_2Cl_2/THF); **^{1}H NMR** (400 MHz, DMSO-d_6) δ 8.62 (t, $J_{CONH,2}$ = 5.6 Hz, 1H, CON*H*), 7.83 (d, $J_{2^i,F}$ = 12.5 Hz, 1H, H-2^i), 7.72 (m, 4H, H-5^i, H-6^i, 2xH-2^{ii}), 6.94 (br t, $J_{NHBoc,1}$ = 5.8 Hz, 1H, N*H*Boc), 6.70 (d, $J_{3^{ii},2^{ii}}$ = 8.9 Hz, 2H, H-3^{ii}), 6.39 (s, 2H, N*H*$_2$), 3.31 (m, 2H, H-2), 3.13 (m, 2H, H-1), 1.37 (s, 9H, C(C*H*$_3$)$_3$); **^{13}C NMR** (100.6 MHz, DMSO-d_6) δ 164.7 (C-3), 157.8 (d, $^1J_{C,F}$ = 253.0 Hz, C-3^i), 155.8 (C, carbamate), 154.0 (C-4^{ii}), 143.4 (C-1^{ii}), 142.0 (d, $^2J_{C,F}$ = 7.3 Hz, C-4^i), 136.1 (d, $^3J_{C,F}$ = 6.7 Hz, C-1^i), 126.1 (C-2^{ii}), 123.8 (d, $^4J_{C,F}$ = 3.3 Hz, C-6^i), 117.2 (C-5^i), 115.8 (d, $^2J_{C,F}$ = 21.4 Hz, C-2^i), 113.5 (C-3^{ii}), 77.7 (*C*(CH$_3$)$_3$), 39.8 (C-1), 39.5 (C-2), 28.3 (C(*C*H$_3$)$_3$); **IR** (ATR) υ 3425, 3342, 3307, 3214, 2938, 1686, 1527, 1279, 1143 cm^{-1}. **HRMS** (ESI-TOF) *m/z* calcd for $[C_{20}H_{24}FN_5O_3 + H]^+$ 402.1936; found 402.1937. **COSY**, **DEPT 135** and **^{1}H/^{13}C** correlation were recorded. See **Supplementary Figure 35** for the ^{1}H and ^{13}C NMR spectra.

***N*-(2-Aminoethyl)-4-[(*E*)-(4-aminophenyl)diazenyl]-3-fluorobenzamide, 5b-2 (Supplementary Figure 22).** To a solution of carbamate **5b-1** (1.09 g, 2.72 mmol) in MeOH (109 mL), HCl 37% (31 mL, 374 mmol) was added dropwise. The mixture was stirred at rt for 90 min until the complete consumption of the starting material. Afterwards, the mixture was neutralised with a saturated aqueous solution of $NaHCO_3$ and an orange precipitate appeared. The suspension was cooled to 0 ºC, filtered and

washed with Et_2O to furnish **5b-2** (770 mg, 2.56 mmol, 94% yield) as an orange solid: **Mp** = 230 ºC (decomp. from Et_2O); **^{1}H NMR** (400 MHz, DMSO-d$_6$) δ 8.99 (t, $J_{CONH,2}$ = 5.5 Hz, 1H, CONH), 7.96 (dd, $J_{2^i,F}$ = 11.8 Hz, $J_{2^i,6^i}$ = 1.8 Hz, 1H, H-2^i), 7.84 (dd, $J_{6^i,5^i}$ = 8.4 Hz, $J_{6^i,2^i}$ = 1.8 Hz, 2H, H-6^i), 7.69 (m, 3H, H-5^i, 2xH-2^{ii}), 6.69 (d, $J_{3^{ii},2^{ii}}$ = 8.9 Hz, 2H, H-3^{ii}), 6.45 (s, 2H, NH_2), 3.54 (m, 2H, H-1), 2.99 (t, $J_{2,1}$ = 5.9 Hz, 2H, H-2); **^{13}C NMR** (100.6 MHz, DMSO-d$_6$) δ 165.0 (C-3), 157.8 (d, $^1J_{C,F}$ = 253.0 Hz, C-3^i), 154.1 (C-4^{ii}), 143.3 (C-1^{ii}), 142.1 (d, $^2J_{C,F}$ = 7.3 Hz, C-4^i), 135.5 (d, $^3J_{C,F}$ = 6.8 Hz, C-1^i), 126.1 (C-2^{ii}), 124.0 (d, $^4J_{C,F}$ = 3.1 Hz, C-6^i), 117.2 (C-5^i), 116.0 (d, $^2J_{C,F}$ = 21.7 Hz, C-2^i), 113.5 (C-3^{ii}), 38.6 (C-2), 37.5 (C-1); **IR** (ATR) υ 3394, 3321, 3052, 2860, 2748, 2083, 1641, 1561, 1168 cm^{-1}. **HRMS** (ESI-TOF) *m/z* calcd for $[C_{15}H_{16}FN_5O + H]^+$ 302.1412; found 302.1410. **^{1}H/^{13}C** correlation was recorded. See **Supplementary Figure 36** for the ^{1}H and ^{13}C NMR spectra.

di-*tert*-Butyl (2*R*,4*S*)-2-(4-{[2-({4-[(*E*)-(4-aminophenyl)diazenyl]-3-fluorobenzoyl}amino)ethyl]amino}-4-oxobutyl)-4-[(*tert*-butoxycarbonyl)amino] pentanedioate, 6b (Supplementary Figure 23). To a solution of amine **5b-2** (200 mg, 0.66 mmol) in anhydrous THF (35 mL), a solution of glutamate **2** (324 mg, 0.72 mmol), HOBt (141 mg, 1.02 mmol), EDCI (0.16 mL, 0.89 mmol) and DIPEA (0.48 mL, 2.74 mmol) in anhydrous THF (24 mL) was added. The reaction mixture was stirred overnight at rt. The orange solution was washed with H_2O (2 x 50 mL) and the resulting aqueous extracts were extracted with CH_2Cl_2 (2 x 30 mL). The combined organic extracts were dried over anhydrous Na_2SO_4 and concentrated under vacuum. The resulting residue was purified by column chromatography (EtOAc) to give **6b** (270 mg, 0.37 mmol, 53% yield) as an orange solid: **Mp**: 85-90 ºC (from EtOAc); **$[\alpha]_D^{20}$** = 2.2 (*c* 0.99, $CHCl_3$); **^{1}H NMR** (400 MHz, $CDCl_3$) δ 7.86 (d, $J_{2iv,3}$iv = 8.7 Hz, 2H, H-2^{iv}), 7.76 (m, 2H, H-2^{iii}, H-5^{iii}), 7.71 (br t, $J_{NH,2}$ii = 4.2 Hz, 1H, CON*H*), 7.65 (dd, $J_{6^{iii},5^{iii}}$ = 8.5 Hz, $J_{6^{iii},2^{iii}}$ = 1.8 Hz, 1H, H-6^{iii}), 6.75 (d, $J_{3^{iv},2^{iv}}$ = 8.7 Hz, 2H, H-3^{iv}), 6.54 (br t, $J_{NH,1^{ii}}$ = 5.0 Hz, 1H, CON*H*), 5.12 (d, $J_{NHBoc,4}$ = 8.6 Hz, 1H, N*H*Boc), 4.25 (s, 2H, N*H*$_2$), 4.17 (m, 1H, H-4), 3.60-3.50 (m, 4H, H-1^{ii}, H-2^{ii}), 2.37 (m, 1H, H-2), 2.25 (m, 2H, H-3^i), 2.11 (m, 1H, H-3), 1.66 (m, 5H, H-3, 2xH-1^i, 2xH-2^i), 1.46 (s, 18H, 2xC(C*H*$_3$)$_3$), 1.43 (s, 9H, C(C*H*$_3$)$_3$); **^{13}C NMR** (100.6 MHz, $CDCl_3$) δ 174.7/174.6 (C-1/C-4^i), 171.7 (C-5), 166.4 (d, $^4J_{C,F}$ = 1.9 Hz, C-3^{ii}), 159.2 (d, $^1J_{C,F}$ = 256.6 Hz, C-3^{iii}), 155.5 (C=O, carbamate), 150.8 (C-4^{iv}), 146.0 (C-1^{iv}), 142.9 (d, $^2J_{C,F}$ = 7.2 Hz, C-4^{iii}), 136.2 (d, $^3J_{C,F}$ = 6.8 Hz, C-1^{iii}), 126.1 (C-2^{iv}), 122.9 (d, $^4J_{C,F}$ = 3.2 Hz, C-6^{iii}), 118.2 (C-5^{iii}), 116.2 (d, $^2J_{C,F}$ = 21.7 Hz, C-2^{iii}), 114.6 (C-3^{iv}), 82.2 (*C*(CH$_3$)$_3$), 81.2 (*C*(CH$_3$)$_3$), 79.9 (*C*(CH$_3$)$_3$), 52.8 (C-4), 42.3 (C-2), 42.2 (C-2^{ii}), 39.7 (C-1^{ii}), 36.2 (C-3^i), 34.9 (C-3), 31.8 (C-1^i), 28.4 (C(*C*H$_3$)$_3$), 28.2 (C(*C*H$_3$)$_3$), 28.1 (C(*C*H$_3$)$_3$), 23.1 (C-2^i); **IR** (ATR) υ 3340, 2978, 1707, 1638, 1600, 1534, 1366, 1142 cm^{-1}. **HRMS** (ESI-TOF) *m/z* calcd for $[C_{37}H_{53}FN_6O_8 + H]^+$ 729.3982; found: 729.3976. **COSY**, **DEPT 135** and

$^1H/^{13}C$ correlation were recorded. See **Supplementary Figure 37** for the ^{1}H and ^{13}C NMR spectra.

di-*tert*-Butyl (2*S*,4*R*)-2-[(*tert*-butoxycarbonyl)amino]-4-(4-{[2-({4-[(*E*)-(4-{[(2,5-dioxo -2,5-dihydro-1*H*-pyrrol-1-yl)acetyl]amino}phenyl)diazenyl]-3-fluorobenzoyl} amino)ethyl]amino}-4-oxobutyl)pentanedioate, 7b (Supplementary Figure 24). To a stirred solution of maleimide acetic acid **3** (73 mg, 0.47 mmol) and oxalyl chloride 98% (43 µL, 0.50 mmol) in dry CH_2Cl_2 (7.0 mL) was added one drop of DMF. After stirring for 2 h at rt, the mixture was concentrated. The resulting acid chloride was taken up in dry THF (3.2 mL) and slowly added to an ice-cooled solution of **6b** (90 mg, 0.12 mmol), DIPEA (0.13 mL, 0.74 mmol) in dry THF (15.5 mL). After stirring for 10 min at this temperature, the mixture was warmed up to rt and stirred overnight. Then, it was diluted with EtOAc (21 mL) and the resulting solution was washed with water (3 x 7 mL). The organic extract was dried over anhydrous Na_2SO_4, concentrated under vacuum and purified by column chromatography (EtOAc) to provide **7b** (90 mg, 0.10 mmol, 84% yield) as an orange solid: **Mp**: 200-205 ºC (from Et_2O); **$[\alpha]_D^{20}$** = -3.1 (*c* 0.99, $CHCl_3$); **^{1}H NMR** (400 MHz, $CDCl_3$, 320 K) δ 7.94 (d, $J_{2^{iv},3^{iv}}$ = 8.2 Hz, 2H, H-2^{iv}), 7.92 (br s, 1H, CON*H*), 7.75 (m, 2H, H-2^{iii}, H-5^{iii}), 7.66 (m, 3H, 2xH-3^{iv}, H-6^{iii}), 7.58 (br s, 1H, CON*H*), 6.86 (s, 2H, H-3^{vi}, H-4^{vi}), 6.35 (br s, 1H, CON*H*), 5.05 (br d, $J_{NHBoc,2}$ = 6.8 Hz, 1H, NHBoc), 4.42 (s, 2H, H-2^{v}), 4.17 (m, 1H, H-2), 3.62 (m, 2H, H-2^{ii}), 3.58 (m, 2H, H-1^{ii}), 2.40 (m, 1H, H-4), 2.26 (m, 2H, H-3^{i}), 2.11 (m, 1H, H-3), 1.70 (m, 5H, H-3, 2xH-1^{i}, 2xH-2^{i}), 1.48 (s, 18H, 2xC(CH_3)$_3$), 1.45 (s, 9H, C(CH_3)$_3$); **^{13}C NMR** (100.6 MHz, $CDCl_3$, 320 K) δ 174.8/174.5 (C-5/C-4^{i}), 171.7 (C-1), 170.3 (C-2^{vi}, C-5^{vi}), 166.4 (C-3^{ii}), 164.6 (C-1^{v}), 159.7 (d, $^1J_{C,F}$ = 259.9 Hz, C-3^{iii}), 155.6 (C=O, carbamate), 149.7 (C-1^{iv}), 142.5 (d, $^2J_{C,F}$ = 7.2 Hz, C-4^{iii}), 140.8 (C-4^{iv}), 137.6 (d, $^3J_{C,F}$ = 7.2 Hz, C-1^{iii}), 134.8 (C-3^{vi}, C-4^{vi}), 124.8 (C-2^{iv}), 123.0 (d, $^4J_{C,F}$ = 3.7 Hz, C-6^{iii}), 120.2 (C-3^{iv}), 118.3 (C-5^{iii}), 116.5 (d, $^2J_{C,F}$ = 21.5 Hz, C-2^{iii}), 82.2 (*C*(CH_3)$_3$), 81.2 (*C*(CH_3)$_3$), 80.0 (*C*(CH_3)$_3$), 53.1 (C-2), 42.5 (C-4), 42.1 (C-2^{ii}), 41.6 (C-2^{v}), 39.8 (C-1^{ii}), 36.3 (C-3^{i}), 34.9 (C-3), 31.9 (C-1^{i}), 28.5 (C(*C*H_3)$_3$), 28.3 (C(*C*H_3)$_3$), 28.2 (C(*C*H_3)$_3$), 23.1 (C-2^{i}); **IR** (ATR) υ 3273, 3088, 2977, 2933, 1715, 1540, 1147 cm^{-1}. **HRMS** (ESI-TOF) *m/z* calcd for $[C_{43}H_{56}FN_7O_{11} + H]^+$ 866.4095; found 866.4087. **COSY**, **DEPT 135** and **$^1H/^{13}C$** correlation were recorded. See **Supplementary Figure 38** for the ^{1}H and ^{13}C NMR spectra.

(1*S*,3*R*)-1,3-Dicarboxy-7-{[2-({4-[(*E*)-(4-{[(2,5-dioxo-2,5-dihydro-1*H*-pyrrol-1-yl) acetyl]amino}phenyl)diazenyl]-3-fluorobenzoyl}amino)ethyl]amino}-7-oxoheptan-1-aminium trifluoroacetate, $MAG_{2P\_F}^{slow}$ (Supplementary Figure 25). To a stirred solution of compound **7b** (90 mg, 0.10 mmol) in CH_2Cl_2 (20 mL), trifluoroacetic acid (7.5 mL, 97.9 mmol) was added. The mixture was stirred at rt until the starting material

was consumed. Then, the solution was concentrated under vacuum and the resulting orange solid was washed with Et_2O to furnish $\mathbf{MAG^{slow}_{2P\_F}}$ (77 mg, 0.10 mmol, quantitative yield): **Mp** > 230 ºC (from Et_2O); $[\alpha]_D^{20}$ = 3.5 (*c* 0.50, DMSO); **^{1}H NMR** (360 MHz, DMSO-d_6) δ 10.77 (s, 1H, CON*H*), 8.88 (t, $J_{NH,2^i}$ = 5.6 Hz, 1H, CON*H*), 8.03 (t, $J_{NH,1^i}$ = 5.7 Hz, 1H, CONH-7), 7.94 (m, 3H, H-2^{ii}, 2xH-2^{iii}), 7.81 (m, 4H, 2xH-3^{iii}, H-5^{ii}, H-6^{ii}), 7.17 (s, 2H, H-3^{v}, H-4^{v}), 4.35 (s, 2H, H-2^{iv}), 3.56 (m, 1H, H-1), 3.33 (m, 2H, H-2^{i}), 3.24 (m, 2H, H-1^{i}), 2.57 (m, 1H, H-3), 2.08 (m, 3H, H-2, 2xH-6), 1.68 (m, 1H, H-2), 1.48 (m, 4H, H-4, H-5); **^{13}C NMR** (90.5 MHz, DMSO-d_6) δ 175.9/172.1 (2xCOOH/C-7), 170.7 (C-2^{v}, C-5^{v}), 165.6 (C-1^{iv}), 164.4 (C-3^{i}), 158.6 (d, $^1J_{C,F}$ = 255.3 Hz, C-3^{ii}), 148.0 (C-4^{iii}), 142.4 (C-1^{iii}), 141.3 (d, $^2J_{C,F}$ = 7.2 Hz, C-4^{ii}), 138.1 (d, $^3J_{C,F}$ = 6.5 Hz, C-1^{iii}), 135.0 (C-3^{v}, C-4^{v}), 124.4 (C-2^{iii}), 124.0 (d, $^4J_{C,F}$ = 3.3 Hz, C-6^{ii}), 119.5 (C-3^{iii}), 117.6 (C-5^{iii}), 116.1 (d, $^2J_{C,F}$ = 21.7, C-2^{ii}), 51.6 (C-1), 40.7 (C-3), 40.5 (C-2^{iv}), 38.7 (C-2^{i}), 38.1 (C-1^{i}), 35.2 (C-6), 32.3 (C-2), 31.1 (C-4), 22.5 (C-5); **IR** (ATR) υ 3271, 3081, 2929, 1712, 1542, 1429, 1147, 1105 cm^{-1}. **HRMS** (ESI-TOF) *m/z* calcd for $[C_{30}H_{33}FN_7O_9]^+$ 654.2318; found 654.2311. **COSY**, **DEPT 135** and **^{1}H/^{13}C** correlation were recorded. See **Supplementary Figure 39** for the ^{1}H and ^{13}C NMR spectra.

4-[(4-Acetylaminophenyl)azo]benzoic acid, 5a-1' (Supplementary Figure 26). To an ice-cooled solution of acid **5a** (500 mg, 2.08 mmol) and pyridine (839 µL, 10.4 mmol) in anhydrous THF (125 mL), acetyl chloride (221 µL, 3.11 mmol) was added dropwise. The mixture was stirred for 2 h at rt. Then, the solution was filtered and the solvent was evaporated under vacuum to give a solid which was recrystallyzed in MeOH to afford **5a-1'** (553 mg, 1.95 mmol, 93% yield) as an orange solid: **Mp** > 300 °C (from MeOH); **^{1}H NMR** (250 MHz, DMSO-d_6) δ 13.20 (s, H, COO*H*), 10.34 (s, H, N*H*CO), 8.12 (d, $J_{2^i,3^i}$ = 8.6 Hz, 2H, H-2^{i}), 7.92 (d, $J_{3^i,2^i}$ = 8.6 Hz, 2H, H-3^{i}), 7.92 (d, $J_{2^{ii},3^{ii}}$ = 9.1 Hz, 2H, H-2^{ii}), 7.83 (d, $J_{3^{ii},2^{ii}}$ = 9.1 Hz, 2H, H-3^{ii}), 2.11 (s, 3H, COC*H*$_3$); **^{13}C NMR** (100.6 MHz, DMSO-d_6) δ 168.9 (*C*OCH$_3$), 165.0 (COOH), 154.5 (C-4), 147.4 (C-1^{ii}), 143.0 (C-4^{ii}), 132.3 (C-1^{i}), 130.6 (C-2^{i}), 124.0 (C-2^{ii}), 122.3 (C-3^{i}), 119.1 (C-3^{ii}), 24.2 (CO*C*H$_3$); **IR** (ATR) υ 3330, 1666, 1592, 1518, 1293 cm^{-1}. **HRMS** (ESI-TOF) *m/z* calcd for $[C_{15}H_{13}N_3O_3 + H]^+$ 282.0884; found 282.0878. See **Supplementary Figure 40** for the ^{1}H and ^{13}C NMR spectra.

***N*-Isopropyl-4-[(4-acetylaminophenyl)azo]benzamide, Azo1' (Supplementary Figure 27).** To a solution of acid **5a-1'** (250 mg, 0.83 mmol) in anhydrous THF (40 mL), a solution of HOBt (171 mg, 1.27 mmol), EDCI (220 mg, 1.15 mmol), DIPEA (615 µL, 3.53 mmol) and isopropylamine (72 µL, 1.06 mmol) in anhydrous THF (20 mL) was added. The reaction mixture was stirred overnight at rt. Evaporation of the solvent under vacuum gave a residue which was purified by column chromatography

(CH_2Cl_2/THF, 4:1) to obtain **Azo1'** as an orange solid (145 mg, 0.45 mmol, 71% yield): **Mp** > 300 ºC (decomp. from CH_2Cl_2/THF); **^{1}H NMR** (360 MHz, DMSO-d_6) δ 10.34 (s, 1H, N*H*CO), 8.38 (d, $J_{NH,CH}$ = 7.8 Hz, 1H, CON*H*CH), 8.03 (d, $J_{2^i,3^i}$ = 8.6 Hz, 2H, H-2^i), 7.90 (d, $J_{3^i,2^i}$ = 8.6 Hz, 2H, H-3^i), 7.90 (d, $J_{2^{ii},3^{ii}}$ = 8.9 Hz, 2H, H-2^{ii}), 7.82 (d, $J_{3^{ii},2^{ii}}$ = 8.9 Hz, 2H, H-3^{ii}), 4.12 (m, $J_{CH,N}$ = 7.8 Hz, $J_{CH,CH3}$ = 6.6 Hz, 1H, C*H*(CH_3)$_2$), 2.10 (s, 3H, COC*H*$_3$), 1.19 (d, $J_{CH3,CH}$ = 6.6 Hz, 6H, CH(C*H*$_3$)$_2$); **^{13}C NMR** (100.6 MHz, DMSO-d_6) δ 169.0 (*C*OCH$_3$), 164.6 (*C*ONH), 153.3 (C-4^i), 147.5 (C-1^{ii}), 142.9 (C-4^{ii}), 136.5 (C-1^i), 128.5 (C-2^i), 124.0 (C-2^{ii}), 122.0 (C-3^i), 119.1 (C-3^{ii}), 41.2 (*C*H(CH_3)$_2$), 24.2 (CO*C*H$_3$), 22.3 (CH(*C*H$_3$)$_2$); **IR** (ATR) ν 3290, 2920, 2851, 1658, 1626, 1523 cm^{-1}. **HRMS** (ESI-TOF) *m/z* calcd for [$C_{18}H_{20}N_4O_2$ + Na]$^+$ 347.1478; found 347.1466. **HMBC** experiment was recorded. See **Supplementary Figure 41** for the ^{1}H and ^{13}C NMR spectra.

4-[(4-Acetylaminophenyl)azo]-3-fluorobenzoic acid, 5b-1' (Supplementary Figure 28).

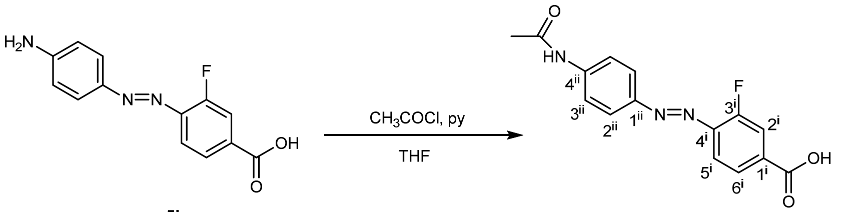

5b **5b-1'**

To an ice-cooled solution of acid **5b** (200 mg, 0.77 mmol) and pyridine (312 µL, 3.86 mmol) in anhydrous THF (50 mL), acetyl chloride (82 µL, 1.16 mmol) was added dropwise. The mixture was stirred for 2 h at rt. Then, the solution was filtered and the solvent was evaporated under vacuum to give a residue that was taken up in a mixture of THF:AcOEt (4:1) (30 mL). The organic lawyer was washed with HCl 1M (2 x 30 mL), dried over anhydrous Na_2SO_4 and concentrated under vacuum. The resulting solid was recrystallyzed using MeOH to afford **5b-1'** (168 mg, 0.56 mmol, 69% yield) as an orange solid: **Mp** > 290 ºC (decomp. from MeOH); ^{1}**H NMR** (400 MHz, DMSO-d_6) δ 13.44 (s, 1H, COO*H*), 10.38 (s, H, N*H*CO), 7.88 (m, 6H, H-3^{ii}/H-2^{ii}/H-2^i/H-6^i), 7.76 (m, 1H, H-5^i), 2.11 (s, 3H, COC*H*$_3$); **^{13}C NMR** (100.6 MHz, DMSO-d_6) δ 169.0 (s, *C*OCH$_3$), 165.8 (d, $^4J_{C-F}$ = 2.3 Hz, *C*OOH), 158.4 (d, $^1J_{C-F}$ = 256.7 Hz, C-3^i), 147.7 (s, C-1^{ii}), 143.5 (s, C-4^{ii}), 142.5 (d, $^2J_{C-F}$ = 7.3 Hz, C-4^i), 134.2 (d, $^3J_{C-F}$ = 7.2 Hz, C-1^i), 125.9 (d, $^4J_{C-F}$ = 3.4 Hz, C-6^i), 124.5 (s, C-2^{ii}), 119.1 (s, C-3^{ii}), 117.9 (s, C-5^i), 117.9 (d, $^2J_{C-F}$ = 21.1 Hz, C-2^i), 22.24 (s, CO*C*H$_3$); **^{19}F{^{1}H }-RMN** (400 MHz, DMSO-d_6) δ -124.3 (s, F); **IR** (ATR) ν 3291, 1662, 1585, 1408, 1293, 1147 cm^{-1}. **HRMS** (ESI-TOF) *m/z* calcd. for [$C_{15}H_{12}N_3O_3F$ + H]$^+$ 300.0790; found 300.0792. **HSQC** and **HMBC** experiments were recorded. See **Supplementary Figures 42** and **43** for the ^{1}H, ^{19}F and ^{13}C NMR spectra.

***N*-Isopropyl-4-[(4-acetylaminophenil)azo]-3-fluoro benzamide, Azo2' (Supplementary Figure 29).** To a solution of acid **5b-1'** (100 mg, 0.33 mmol) in anhydrous THF (10 mL), a solution of HOBt (67 mg, 0.50 mmols), EDCI (83 mg, 0.43 mmol), DIPEA (231 µL, 1.33 mmol) and isopropylamine (34 µL, 0.40 mmol) in anhydrous THF (10 mL) was added under N_2 atmosphere. The reaction mixture was stirred overnight at rt. Then, the solution was filtered and the solvent was evaporated under vacuum to give a solid residue that was washed with CH_2Cl_2 (5 mL). After filtration, the solid was recrystallyzed using MeOH to afford **Azo2'** (89 mg, 0.26 mmol, 75% yield) as an orange solid: **Mp**: 278-280 ºC (from MeOH); **^{1}H NMR** (360 MHz, DMSO-d_6) δ 10.37 (s, 1H, N*H*CO), 8.44 (d, $J_{NH,CH}$ = 7.6 Hz, 1H, CON*H*CH), 7.92 (m, 3H, H-2^{ii}/H-2^{i}), 7.82 (m, 3H, H-3^{ii}/H-6^{i}), 7.74 (m, 1H, H-5^{i}), 4.11 (m, 1H, C*H*$(CH_3)_2$), 2.11 (s, 3H, COC*H*$_3$), 1.19 (d, $J_{CH3,CH}$ = 6.6 Hz, 6H, CH(C*H*$_3$)$_2$); **^{13}C NMR** (100.6 MHz, DMSO-d_6) δ 168.9 (s, *C*OCH$_3$), 163.3 (d, $^4J_{C\text{-}F}$ = 1.8 Hz, *C*ONHCH), 158.5 (d, $^1J_{C\text{-}F}$ = 255.2 Hz, C-3^{i}), 147.7 (s, C-1^{ii}), 143.5 (s, C-4^{ii}), 141.2 (d, $^2J_{C\text{-}F}$ = 7.2 Hz, C-4^{i}), 138.2 (d, $^3J_{C\text{-}F}$ = 6.8 Hz, C-1^{i}), 124.3 (s, C-2^{ii}), 123.9 (d, $^4J_{C\text{-}F}$ = 3.3 Hz, C-6^{i}), 119.1 (s, C-3^{ii}), 117.5 (s, C-5^{i}), 116.5 (d, $^2J_{C\text{-}F}$ = 21.3 Hz, C-2^{i}), 41.3 (s, *C*H(CH$_3$)$_2$), 24.2 (s, CO*C*H$_3$), 22.2 (s, CH(*C*H$_3$)$_2$); **^{19}F{^{1}H}-RMN** (400 MHz, DMSO-d_6) δ -124.6 (s, F); **IR** (ATR) υ 3293, 2964, 1672, 1637, 1533 cm^{-1}. **HRMS** (HR-ESI+) *m/z* calcd for $[C_{18}H_{19}N_4O_2F + Na]^+$ 365.1384; found 365.1389. See **Supplementary Figure 44** and **45** for the ^{1}H, ^{19}F and ^{13}C NMR spectra.

Photochemical characterization of MAG^{slow}_{2P} and $MAG^{slow}_{2P\_F}$: All spectroscopic and photochemical experiments were carried out in HPLC- or spectroscopy-quality solvents and in Ar-degassed samples. Steady-state UV-vis absorption measurements were recorded on a HP 8453 spectrophotometer with temperature control. Isomerization quantum yields were determined relative to azobenzene in acetonitrile ($\Phi^{\pi\rightarrow\pi^*}_{trans\rightarrow cis,\ \mathrm{azobenzene}}$ = 0.15[8] and $\Phi^{n\rightarrow\pi^*}_{cis\rightarrow trans,\ \mathrm{azobenzene}}$= 0.46[8]). Different excitation sources were used in the photochemical experiments depending on the spectral requirements: a Xe lamp coupled to a spectrograph, the third harmonic of a ns-pulsed Nd:YAG laser (λ_{exc} = 355 nm, Brilliant, Quantel), and cw DPSS lasers (λ_{exc} = 405 nm, SciTec; λ_{exc} = 473 nm, SciTec).

Cell line and transfection: HEK293 tsA201 cell line (SV40-transformed, Human Embryonic Kidney 293 cells) was maintained at 37 ºC in a 5% CO_2 humid incubator with Dulbecco's Modified Eagle Medium/Nutrient Mixture F-12 media (DMEM) (1:1, Invitrogen) supplemented with 10% Fetal Bovine Serum (FBS) and 1% Penicillin/Streptomycin. The expression plasmids for GluK2-L439C and GluK2-L439C-eGFP were kindly provided by Ehud Y. Isacoff (University of California) and subcloned

as previously described.[9] Cells were transfected with pcDNA3-GluK2-L439C-eGFP for electrophysiology, co-transfected with pcDNA3-GluK2-L439C:GCaMP6s (1:1) for 1P calcium imaging, and with pcDNA3-GluK2-L439C-eGFP::RGECO1 (2:1) for 2P calcium imaging.

DNA–X-tremGENE 9 Transfection Reagent (Roche) mix was used following manufacturer's instructions with a Reagent:DNA ratio of 3:1. The mix was incubated for 20 min at room temperature, meanwhile cells were placed into a 6-multiwell plate at a density of $7.5 \cdot 10^5$ cells per well. After incubation, the mix was added dropwise into each well. The day after, cells were harvested with accutase (Sigma-Aldrich) and seeded onto 16-mm glass coverslips (Fisher Scientific) pretreated with poly-L-lysine (Sigma-Aldrich) to allow cell adhesion. Electrophysiologcal experiments were performed 24–48 h after transfection, with cells plated at low density. One-photon calcium imaging experiments were performed 48–72 h after transfection with cells plated on PLL-coverslips at high density. Two-photon calcium imaging experiments were performed 48 h after transfection with cells plated at high density on 25 mm diameter glass coverslips (Fisher Scientific) pretreated with PLL into a 6-multiwell plate.

Conjugation of LiGluR with photoswitched tethered ligands: All MAG-type compounds were stored at 10 mM in DMSO at –20 ºC. Before each experiment cells were incubated with one of the compounds for 10 min in the absence of light in an extracellular solution (ES) composed of (in mM): 140 NaCl, 1 $MgCl_2$, 2.5 KCl, 10 HEPES, 0.5 $CaCl_2$ and 10–20 glucose to fix osmolarity to 300 mOsm·kg^{-1}, while pH 7.4 was adjusted with NaOH. For HEK cell line, **MAG**, $\mathbf{MAG_{2P}^{slow}}$, $\mathbf{MAG_{2P\_F}^{slow}}$ or $\mathbf{MAG_{2p}}$ were added at a concentration of 50 μM. After incubation, cells were washed three times with ES and incubated 10 min with 0.3 mg mL^{-1} Concanavalin A (Sigma) -to block GluK2 desensitization- on an ES based on NMDG$^+$ (to avoid depolarization due to open LiGluRs, in mM): 110 NMDG+, 2.5 KCl, 1 $MgCl_2$, 10 HEPES, 10–20 glucose to fix osmolarity to 300 mOsm·kg^{-1}, while pH 7.4 was adjusted with HCl. Before placing the coverslip to the recording chamber, cells were washed again three times with ES.

Electrophysiology: For one-photon stimulation, voltage-clamp recordings under whole-cell configuration were done using an EPC-10 amplifier and data at 10 kHz was acquired with amplifier's software Patch Master (HEKA). Bath solution was composed of (in mM): 140 NaCl, 1 $MgCl_2$, 2.5 KCl, 10 HEPES, 2.5 $CaCl_2$ and 10–20 glucose to fix osmolarity to 310 mOsm·kg^{-1}, while pH 7.42 was adjusted with NaOH. Borosilicate glass pipettes were pulled with a typical resistance of 4–6 MΩ for HEK293 cells. Pipette solution contained (in mM): 120 cesium methanosulfonate, 10 TEA-Cl, 5 $MgCl_2$,

3 Na_2ATP, 1 Na_2GTP, 20 HEPES, 0.5 EGTA; osmolarity was 290 mOsm·kg^{-1} and pH 7.2 was adjusted with CsOH.
One-photon action spectrum characterization was done by illumination of the entire focused field using a Polychrome V monochromator (TILL Photonics) connected through the back port of an IX71 inverted microscope (Olympus) with a XLUMPLFLN 20XW x20/1 water immersion objective (Olympus). For automatically controlling wavelength, the monochromator was connected to the EPC-10 amplifier via Photochromic Manual Control (TILL Photonics) and controlled with the photometry module of Patch Master. During voltage-clamp recordings we applied a train of 1 s light-pulses at different wavelengths (for the whole action spectrum, we ranged wavelengths from 300 to 600 nm, with 10 nm steps) with 5 s delay between pulses in which light was switched to 500 nm for **MAG**, **MAG_{2P}^{slow}** and **$MAG_{2P\_F}^{slow}$** (to close LiGluR) or 690 nm for **MAG_{2p}**. Light power density measured with a Newport 1916-C light meter after the objective was 22.0 μW mm^{-2} for 380 nm, 45.9 μW mm^{-2} for 460 nm and 47.4 μW mm^{-2} for 500 nm.

Calcium imaging with 1P stimulation: Cells were imaged on an inverted fully motorized digital microscope (iMic 2000, Till Photonics) controlled with the Live Acquisition 2.1 software (Till Photonics). Images were acquired at room temperature with a UV Apochromat 40× oil objective lens (Olympus) with an imaging interval of 2 s. GCaMP6s was excited during 10 ms at 490 nm by using a Polychrome V light source (Till Photonics) equipped with a Xenon Short Arc lamp (Ushio) and a 505-nm dichroic beam splitter (Chroma Technology). Emission was filtered by a D535/40nm emission filter (Chroma Technology) and finally collected by a cooled CCD camera (Interline Transfer IMAGO QE, Till Photonics).
Addition of agonists was carried out by carefully pipetting 100 μl of a 3 mM glutamate stock solution directly into the accessory pool of the recording chamber (final concentration 300 μM). Cells with no response to free glutamate were excluded from the analysis.
Photoisomerization was achieved by illuminating the focused sample with flashes of violet (380 nm, 0.5 s duration) light for activation, and flashes of green (500 nm, 0.5 s duration) light for deactivation, using the same light source as for the dye excitation. Calcium imaging activation spectra at one-photon ranged from 280 to 480 increasing 20 nm at a time. Light flashes were nested in between GCaMP6s fluorescence measures keeping the frame rate. Photostimulation intervals lasted a total of 3.2 min for activation and 2.4 min for deactivation.

Calcium imaging with 2P stimulation: All two-photon experiments were performed in the Advanced Digital Microscopy Core Facility of IRB Barcelona with a SP5 spectral confocal multiphoton microscope (Leica) equipped with: a 405 nm cw diode laser, an Ar laser (514nm), and a pulsed broadband Ti:Sapphire laser (Mai Tai, Spectra-Physics, Santa-Clara, CA-USA) which can be tuned from 710-990 nm (80 MHz repetition rate, 80 fs pulse). We used a 40x/1.25-0.75-NA Oil objective (HCX PL APO, Leica).
In HEK cell experiments, R-GECO1 was used as a Ca^{2+} fluorescent indicator instead of gcamp6 because it does not absorb at 405 nm, the excitation wavelength used to test the 1P activity in the confocal multiphoton microscope employed. Imaging of R-GECO1 was done at was done at 561 nm (6.37 uW μm^{-2}) with a frame rate of 4 s, a minimal exposition time of 343 ms, and using bidirectional laser scanning at 400 Hz. Images were recorded with a HyD detector with a detection range from 569 to 648 nm, at 512x512 pixel resolution. Pinhole aperture was set at maximum (600 µm).
Photostimulation flashes were fit to keep imaging interval, and periods lasted in total 1 min. One-photon photostimulation was done at 405 nm (0.37 mW μm^{-2}), and two-photon stimulation was done at 780 nm (2.8 mW μm^{-2}). Back-photoisomerization was achieved at 514 nm (0.35 mW μm^{-2}). Inter-stimulus imaging periods lasted 1 min. Photostimulation was done at 256x256 resolution with bidirectional laser scan (400 Hz) by zooming in (x3) in the center of the image.
To characterize the wavelength dependence of two-photon LiGluR activation, we measured photoresponses at different wavelengths: from 720 nm to 840 nm for $\mathbf{MAG^{slow}_{2P\_F}}$, and from 740 nm to 820 nm for **MAG** and $\mathbf{MAG^{slow}_{2P}}$.
Imaging conditions were adjusted to obtain the best signal to noise ratio and minimal photobleaching of the sample during long temporal recordings. Wavelength, intensity and duration of the photostimulation intervals were adjusted to obtain optimal photoresponses with high reproducibility while keeping cell integrity

Hippocampal slice culture and gene transfection: All procedures were conducted in accordance with the European guidelines for animal care and use in research, and were approved by the Animal Experimentation Ethics Committee at the University of Barcelona (Spain). Hippocampal organotypic slice cultures were prepared from postnatal day 6-7 rats as described.[10] Slices were cultured at 35 ºC on interface membranes (Millipore) and fed with MEM media containing 20% horse serum, 27 mM D-glucose, 6 mM $NaHCO_3$, 2 mM $CaCl_2$, 2 mM $MgSO_4$, 30 mM HEPES, 0.01 % ascorbic acid and 1 µg/ml insulin. pH was adjusted to 7.3 and osmolality to 300-320 mOsm kg^{-1}. Slices were biolistically transfected (BioRad) after 5-7 DIV with GluK2-L439C-eGFP and RCaMP2a (Addgene) under CAG promoter as described.[11-12]

PTL conjugation in hippocampal slice culture: For hippocampal organotypic slice cultures, MAG or $MAG^{slow}_{2P\_F}$ were incubated at 250 µM in a 12-multiwell plate in artificial cerebrospinal fluid (ACSF) containing: 119 mM NaCl, 2.5 mM KCl, 3 mM $CaCl_2$, 0.2 mM $MgCl_2$, 26.2 mM $NaHCO_3$, 1 mM NaH_2PO_4 and 11 mM glucose, equilibrated with 5% CO_2/95% O_2. Three different steps of washout with fresh ACSF were done in different wells of the same plate, lasting 1 min, 5 min and 5 min. Afterwards, slices were placed on the recording chamber. Slices were maintained at room temperature (r.t., 25-27 °C) in a continuous perfusion of ACSF.

Calcium imaging with 2P stimulation: Time-lapse fluorescence imaging was carried out in the Advanced Digital Microscopy Core Facility of IRB Barcelona with a SP5 spectral confocal multiphoton microscope (Leica) equipped with a 405 nm cw diode laser, an Argon laser (488 and 514 nm), and a pulsed broadband Ti:Sapphire laser (Mai Tai, Spectra-Physics, Santa-Clara, CA-USA) which can be tuned from 710-990 nm (80 MHz repetition rate, 80 fs pulse). We used a 40x/1.25-0.75-NA Oil objective (HCX PL APO, Leica).

Imaging was performed at 8-15 DIV in pyramidal neurons co-expressing GluK2-L439C-eGFP and RCaMP2 in a single focused plane. Selected neurons presented healthy morphology and no signs of fluorescent aggregates.

Green and red fluorescent proteins were simultaneously excited at 488 nm for 343 ms, using bidirectional laser scanning at 400 Hz. Images were recorded with a resolution of 512x512, and with an imaging interval of 4 s. Green and red fluorescence were recorded with two different HyD detectors with a detection range from 500 to 550 nm and from 569 to 648 nm, respectively. Pinhole aperture was set at maximum (600 µm).

Whole field photostimulation flashes were fit to keep imaging interval, and periods lasted in total for 1 min. Photostimulation was done at 256x256 resolution with bidirectional laser scan. One-photon photostimulation was done at 405 nm (0.81 mW μm^{-2}), and two-photon stimulation at 780 nm (2.8 mW μm^{-2}). Back-photoisomerization was achieved at 514 nm (0.35 mW μm^{-2}). Inter-stimulus imaging periods lasted 1.5 min. Intensity and duration of the photostimulation intervals were adjusted to obtain the optimal photoresponse and reproducibility. At the end of each experiment we reconfirmed that the neuron kept its healthy morphology.

***C.elegans* nematodes and calcium imaging with 2P stimulation in vivo:** KG1180 [lite-1(ce314)X] worms were obtained from CGC and GN692 [IjSi123;lite-1(ce314)X] was a kind gift of Dr Miriam Goodman.[13] We generated strains MSB104 [mirEx22(mec-17p::iGluR6::mCherry;myo-2p::mCherry);IjSi123(mec-7p:GCaMP6s: :SL2::tagRFP);lite-

1(ce314)X]. Standard nematode growth medium and conditions were used for *C. elegans* growth and maintenance.[14]

Molecular constructs pNMSB18 (mec-17p::LiGluR::mCherry::unc-54 3'UTR) were generated by Gibson assembly with the primers indicated in the table below. Transgenesis was performed according to standard methods for microinjection.[11] To generate MSB104 strain a DNA mix containing 50 ng/ul pNMSB18, 1.5 ng/ul myo-2p:mCherry and 50 ng/ul Plus DNA ladder as carrier was injected into the gonad of GN692 young adult worms. The Primers used for Gibson assembly in the construction of pNMSB18 are shown in Supplementary Table 7

$\mathbf{MAG^{slow}_{2P\_F}}$ was administered to the worms by microinjection into the body cavity. A 10 mM solution in 10% DMSO and M9 (22 mM KH2PO4, 42 mM Na_2HPO_4, 86 mM NaCl, 1 mM $MgSO_4$) was freshly prepared. Control group was performed microinjecting with vehicle (10% DMSO in M9). In the case of MSB104, Concanavalin A was also added to the injection mix at a final concentration of 0.3 mg/ml. After compound administration worms were allowed to recover for a minimum of one hour.

Calcium imaging with 2P stimulation: As mentioned above, time-lapse fluorescence imaging was carried out in the Advanced Digital Microscopy Core Facility of IRB Barcelona. We used a 63x/1.4-NA Oil objective (HCX PL APO, Leica).

Imaging was performed 4 h after compound injection in TRN neurons co-expressing GluK2-L439C-mCherry and GCaMP6s in a single focused plane. Neurons with healthy morphology and no signs of fluorescent aggregates were selected for photostimulation.

Green and red fluorescent proteins were simultaneously excited at 488 nm and 561 nm for 343 ms, using bidirectional laser scanning at 400 Hz. Images were recorded with a resolution of 512x512 and a digital zoom of 4, with an imaging interval of 660 ms. Green and red fluorescence were recorded with two different HyD detectors with a detection range from 500 to 550 nm and from 569 to 648 nm, respectively. Pinhole aperture was set at ~500 µm.

Whole field photostimulation flashes were fit to keep imaging interval. Photostimulation was done at 256x256 resolution with bidirectional laser scan, with a digital zoom of 4. One-photon photostimulation was done at 405 nm (15 µW μm^{-2}), and two-photon stimulation at 780 nm (2.8 mW μm^{-2}). Back-photoisomerization was achieved at 514 nm (1.2 µW μm^{-2}). Intensity and duration of the photostimulation intervals were adjusted to obtain the optimal photoresponse and reproducibility. At the end of each experiment we reconfirmed that the neuron kept its healthy morphology.

Data analysis and statistics: Amplitude of LiGluR photocurrents were analyzed using IgorPro (Wavemetrics). Displayed whole-cell current traces have been filtered using the

infinite impulse response digital filter from IgorPro (low-pass filter with cutoff of 50 Hz). The drift in current observed during light spectra recordings was corrected where appropriate with the IgorPro (WaveMetrics) software using a custom-made macro for drift correction.

1P and 2P calcium images were acquired with the Live Acquisition 2.1 software (Till Photonics) and stored by the Arivis Browser 2.5.5 (Arivis AG). These images were analyzed with ImageJ and the mean fluorescence value for each cell profile was calculated using the same software. The fluorescence signals were treated to obtain $\Delta F/F$ values according to:

$$\frac{\Delta F}{F} = \frac{F\text{-}F_0}{F_0} \quad (1)$$

where F_0 is each cell's average signal for the experiment's baseline and F is the fluorescence signal upon stimulation. The resulting fluorescence ratios were analyzed in OriginLab. To obtain cell-averaged 1P action spectra, $\Delta F/F$ values were first normalized with respect to the maximum photoresponse obtained for each cell after perfusion of free glutamate at the end of the experiment. To obtain cell-averaged 2P action spectra, 2P $\Delta F/F$ responses were first normalized with respect to the 1P $\Delta F/F$ response at 405 nm for the same cell.

Supplementary References

[1] Gorostiza, P., Volgraf, M., Numano, R., Szobota, S., Trauner, D. & Isacoff, E. Y. Mechanisms of photoswitch conjugation and light activation of an ionotropic glutamate receptor. *Proc. Natl. Acad. Sci. USA*, **104**, 10865-10870 (2007).

[2] Numano, R. et al. Nanosculpting reversed wavelength sensitivity into a photoswitchable iGluR. *Proc. Natl. Acad. Sci. U. S. A.* **106**, 6814–9 (2009).

[3] Reiner, A. & Isacoff, E. Y. Tethered ligands reveal glutamate receptor desensitization depends on subunit occupancy. *Nat. Chem. Biol.* **10**, 273-280 (2014).

[4] Murugan, N.A., Kongsted, J., Rinkevicius, Z., Aidas, K., Mikkelsen, K. V. & Agren, H. Hybrid density functional theory/molecular mechanics calculations of two-photon absorption of dimethylamino nitro stilbene in solution. *Phys. Chem. Chem. Phys.* **13**, 12506-12516 (2011).

[5] Gascón-Moya, M. et al. An optimized glutamate receptor photoswitch with sensitized azobenzene isomerization. *J. Org. Chem.* **80**, 9915-9925 (2015).

[6] Pearson, R. J., Kassianidis, E., Slawin, A. M. Z. & Philp, D. Self-replication vs. reactive binary complexes—manipulating recognition-mediated cycloadditions by simple structural modifications. *Org. Biomol. Chem.* **2**, 3434–3441 (2004).

[7] Carta, F., Maresca, A., Scozzafava, A. Vullo, D. & Supuran, C. Carbonic anhydrase inhibitors. Diazenylbenzenesulfonamides are potent and selective inhibitors of the tumor-associated isozymes IX and XII over the cytosolic isoforms I and II. *Bioorg. Med. Chem.* **17**, 7093-7099 (2009).

[8] Bortolus, P. & Monti, S. Cis-trans photoisomerization of azobenzene. Solvent and triplet donors effects. *J. Phys. Chem.* **83**, 648–652 (1979).

[9] Izquierdo-Serra M. et al. Two-photon neuronal and astrocytic stimulation with azobenzene-based photoswitches. *J. Am. Chem. Soc.* **136**, 8693-8701 (2014).

[10] Okamoto, K., Nagai, T., Miyawaki, A. & Hayashi, Y. Rapid and persistent modulation of actin dynamics regulates postsynaptic reorganization underlying bidirectional plasticity. *Nat. Neurosci.* **7**, 1104-1112 (2004).

[11] Bosch, M., Castro, J., Sur, M. & Hayashi, Y. Photomarking Relocalization Technique for Correlated Two-Photon and Electron Microcopy Imaging of Single Stimulated Synapses. *Methods Mol. Biol.* **1538**, 185-214 (2017).

[12] Bosch, M., Castro, J., Saneyoshi, T., Matsuno, H., Sur. M. & Hayashi, Y. Structural and molecular remodeling of dendritic spine substructures during long-term potentiation. *Neuron* **16**, 444-459 (2014).

[13] Nekimken, A. L., Mazzochette, E. A., Goodman, M. B. & Pruitt, B. L. Forces applied during classical touch assays for Caenorhabditis elegans. *PLoS One* **12**, 1–11 (2017).

[14] Stiernagle, T. Maintenance of C. elegans. *WormBook* 1–11 (2006). doi:10.1895/wormbook.1.101.1 .

[15] Evans, T. Transformation and microinjection. *WormBook* 1–15 (2006). doi:10.1895/wormbook.1.108.1 .
