# Supplementary material for: Rationally designed azobenzene photoswitches for efficient two-photon neuronal excitation
Source: Nat Commun. 2019 Feb 22;10:907. doi: 10.1038/s41467-019-08796-9 (PMC6385291; doi:10.1038/s41467-019-08796-9)
Supplement: Supplementary file 3 — Description of Additional Supplementary Files [file 41467_2019_8796_MOESM3_ESM.docx]

Description of Additional Supplementary Files

File Name: Supplementary Movie 1

Description: Movie of the light-induced calcium imaging response of cultured HEK293 co-expressing GluK2-L439C and R-GECO1 after conjugation to **MAG**. Four consecutive 1P (x2, 405 nm, power density = 0.37 mW μm^-2^) and 2P excitation scans (x2, 780 nm, power density = 2.8 mW μm^-2^) were applied to open LiGluR channels and trigger calcium-induced R-GECO1 fluorescence enhancement, while 1P excitation scans (x4, 514 nm, power density = 0.35 mW μm^-2^) were applied to revert back the process in all the cases. Imaging of R-GECO1 fluorescence was performed at 514 nm with a frame rate of 3 s and an exposition time of 343 ms. The movie is sped up by a factor of 45 (15 frames per second).

File Name: Supplementary Movie 2

Description: Movie of the light-induced calcium imaging response of cultured HEK293 co-expressing GluK2-L439C and R-GECO1 after conjugation to $\text{MAG}_{\text{2P}}^{\text{slow}}$. Four consecutive 1P (x2, 405 nm, power density = 0.37 mW μm^-2^) and 2P excitation scans (x2, 780 nm, power density = 2.8 mW μm^-2^) were applied to open LiGluR channels and trigger calcium-induced R-GECO1 fluorescence enhancement, while 1P excitation scans (x4, 514 nm, power density = 0.35 mW μm^-2^) were applied to revert back the process in all the cases. Imaging of R-GECO1 fluorescence was performed at 514 nm with a frame rate of 3 s and an exposition time of 343 ms. The movie is sped up by a factor of 45 (15 frames per second).

File Name: Supplementary Movie 3

Description: Movie of the light-induced calcium imaging response of a rat hippocampal neuron co-expressing GluK2-L439C-eGFP and RCaMP2 after conjugation to **MAG**. Two consecutive 1P (x1, 405 nm, power density = 0.81 mW μm^-2^) and 2P excitation scans (x1, 780 nm, power density = 2.8 mW μm^-2^) were applied to open LiGluR channels and trigger calcium-induced RCaMP2 fluorescence increase, while 1P excitation scans (x4, 514 nm, power density = 0.35 mW μm^-2^) were applied to revert back the process in all the cases. Imaging of RCaMP2 fluorescence was performed at 488 nm with a frame rate of 4 s and an exposition time of 343 ms. The movie is sped up by a factor of 60 (15 frames per second).

File Name: Supplementary Movie 4

Description: Movie of the light-induced calcium imaging response of a rat hippocampal neuron co-expressing GluK2-L439C-eGFP and RCaMP2 after conjugation to $\text{MAG}_{\text{2P\_F}}^{\text{slow}}$. Two consecutive 1P (x1, 405 nm, power density = 0.81 mW μm^-2^) and 2P excitation scans (x1, 780 nm, power density = 2.8 mW μm^-2^) were applied to open LiGluR channels and trigger calcium-induced RCaMP2 fluorescence increase, while 1P excitation scans (x4, 514 nm, power density = 0.35 mW μm^-2^) were applied to revert back the process in all the cases. Imaging of RCaMP2 fluorescence was performed at 488 nm with a frame rate of 4 s and an exposition time of 343 ms. The movie is sped up by a factor of 60 (15 frames per second).

File Name: Supplementary Movie 5

Description: Animation of a z-stack rotating around the X axis. Z stack was done at 512x512 of resolution, imaging GCaMP6s and mCherry fluorescence at 488 nm and 561 nm, respectively. Step size was 0.5 μm. The movie is sped up by a factor of 60 (15 frames per second).

File Name: Supplementary Movie 6

Description: Movie of the light-induced calcium imaging response in vivo in *C.elegans* nematodes expressing GCaMP6s and LiGluR-mCherry after conjugation to $\text{MAG}_{\text{2P\_F}}^{\text{slow}}$. 1P excitation (x4, 405 nm, power density = 15 μW μm^-2^) was applied to open LiGluR channels and trigger calcium-induced GCaMP6s fluorescence increase, while 1P excitation scans (x4, 514 nm, power density = 1.2 μW μm^-2^) were applied to revert back the process in all the cases. Imaging of GCaMP6s fluorescence was performed at 488 nm with a frame rate of 660 ms and an exposition time of 343 ms. The movie is sped up by a factor of 60 (15 frames per second).

File Name: Supplementary Movie 7

Description: Movie of the light-induced calcium imaging response in vivo in *C.elegans* nematodes expressing GCaMP6s and LiGluR-mCherry after conjugation to $\text{MAG}_{\text{2P\_F}}^{\text{slow}}$. 2P excitation (x4, 780 nm, power density = 2.8 mW μm^-2^) was applied to open LiGluR channels and trigger calcium-induced GCaMP6s fluorescence increase, while 1P excitation scans (x4, 514 nm, power density = 1.2 μW μm^-2^) were applied to revert back the process in all the cases. Imaging of GCaMP6s fluorescence was performed at 488 nm with a frame rate of 660 ms and an exposition time of 343 ms. The movie is sped up by a factor of 60 (15 frames per second).
